# New role of phenothiazine derivatives as peripherally acting CB1 receptor antagonizing anti-obesity agents

**DOI:** 10.1038/s41598-018-20078-w

**Published:** 2018-01-26

**Authors:** Mayank Kumar Sharma, Jatin Machhi, Prashant Murumkar, Mange Ram Yadav

**Affiliations:** Faculty of Pharmacy, Kalabhavan Campus, The Maharaja Sayajirao University of Baroda, Vadodara, 390 001 Gujarat India

## Abstract

Developing peripherally active cannabinoid 1 (CB1) receptor antagonists is a novel therapeutic approach for the management of obesity. An unusual phenothiazine scaffold containing CB1R antagonizing hit was identified by adopting virtual screening work flow. The hit so identified was further modified by introducing polar functional groups into it to enhance the polar surface area and decrease the hydrophobicity of the resulting molecules. CB1 receptor antagonistic activity for the designed compounds was computed by the previously established pharmacophore and three dimensional quantitative structure–activity relationship models. Docking studies of these designed compounds confirmed the existence of favourable interactions within the active site of the CB1 receptor. The designed compounds were synthesized and evaluated for their CB1 receptor antagonistic activity. Parallel artificial membrane permeability assay was performed to evaluate their potential to permeate into the central nervous system wherein it was observed that the compounds did not possess the propensity to cross the blood brain barrier and would be devoid of central nervous system side effects. In pharmacological evaluation, the synthesized compounds (**23, 25, 27** and **34**) showed significant decrease in food intake suggesting their potential application in the management of obesity through CB1 receptor antagonist activity.

## Introduction

Obesity is an outcome of sustained energy imbalance between calorie intake and energy expenditure. This energy imbalance may be caused due to physical inactivity and/or sedentary life style^1^. The overweight and obese population is increasing with an alarming rate day by day. According to World Health Organization report in 2014, more than 1.9 billion adult population was overweight, of which over 600 million adults were obese, while 41 million children below the age of 5 years were overweight or obese. Overweight condition and obesity are measured by body mass index (BMI), a simple index of weight-for-height. BMI equal to or greater than 25 kg/m^2^ and 30 kg/m^2^ indicates overweight condition and obesity respectively^2^. Unfortunately, obesity is linked to a number of chronic diseases such as diabetes mellitus, hypertension, non-alcoholic fatty liver disease, sleep apnoea, dyslipidemia, osteoarthritis and cancer^1–3^. Therefore, obesity has become a major health problem for the entire human fraternity.

A few drugs such as orlistat, lorcaserin, qsymia, contrave, phentermine etc. have been approved by Food and Drug Administration as anti-obesity agents while some others such as sibutramine and rimonabant have been withdrawn due to their serious side effects^4^. The existing approved drugs have also showed significant side effects. Practically no single drug is available which could be called as an ideal or safe drug for the treatment of obesity. So, there is an unmet medical need to discover newer drugs for the management of this health condition that would have high efficacy and low adverse effects^4–7^.

Endocannabinoid system (ECS) offers a cue for the development of anti-obesity agents. ECS consists of endocannabinoids, some enzymes and cannabinoid receptors (CB1R and CB2R)^8^. CB1 receptors are present in central nervous system (CNS) such as brain stem, hypothalamus, cerebellum and mesolimbic region, and in peripheral tissues such as eyes, mouth and oral cavity, cardiovascular system, pancreas, liver, gastrointestinal tract (GIT), immune system, skin, bones and skeletal muscles, while CB2 receptors are present mainly in the peripheral immune system^3,9^. CB1 receptors are coupled to the G_i/o_ family of G proteins. Activation of CB1 receptors involves signal transduction pathways associated with inhibition of adenylyl cyclase, and to phosphorylation and activation of mitogen-activated protein kinases (MAPK) including p42/p44 MAPK, p38 MAPK and c-Jun N-terminal kinase and extracellular signal-regulated kinases ½ (ERK1/2)^10^. CB1 receptors can couple negatively to N- and P/Q-type voltage-operated calcium channels, and positively to A-type and inwardly rectifying potassium channels. They may induce elevation in intracellular calcium through G-protein dependent activation of phospholipase C-β (PLC-β). All put together these complex signaling cascades regulate various biological activities modulated by CB1 receptors^11^. ECS is involved in physiological functions such as regulation of appetite, energy homeostasis, pain and emotions^12,13^.

Abundant presence of CB1 receptors centrally and peripherally are believed to play an important role in controlling the eating behavior. Over-activation of CB1R leads to increased food intake^14^. Stimulation of CB1 receptors in the CNS triggers signals for enhanced feeding behaviour^15^. The hypothalamic areas play a pivotal role in central control of food intake and feeding behavior. Presence of CB1R in the areas of hypothalamic nuclei indicates that ECS is directly involved in the feeding regulation. These areas are also interconnected with the mesolimbic dopamine pathways^16^. Feeding is modulated by the hypothalamic ECS by decreasing satiety signals and enhancing orexigenic signals^17^. Centrally acting CB1R agonists increase appetite drive by multiple mechanisms involving countering of the inhibitory influence of gamma-aminobutyric acid (GABA) interneurons present in the mesolimbic pathways^16^. Administration of THC, a CB1R agonist into the nucleus accumbens increases sucrose-induced hedonic activity and dopamine release while, CB1R antagonists reduce the extracellular dopamine release in the nucleus accumbens^18^. Endocannabinoids could be regulating the food intake through a neuronal population from hippocampus having a vital role in hedonic aspect of eating^19^. The hypothalamic endocannabinoids are under negative control of leptin secreted as an anorexigenic factor^20^. The stimulating effect of ghrelin on appetite is probably mediated by central activation of ECS because ghrelin enhances levels of endocannabinoids in hypothalamus^21^.

Over-activation of the endocannabinoid/CB1R system can be blocked by antagonizing the CB1 receptors, making CB1 receptors as an attractive target to control obesity by regulating the feeding behaviour^8^. Rimonabant (**1**), the first CB1 receptor antagonist approved by the European Commission as an anti-obesity drug in the year 2006 showed both metabolic benefits and body weight reduction in overweight and obese subjects. But due to its psychiatric side effects i.e. irritability, depression, anxiety, suicidal tendency, neurological and GIT disorders caused by its entry into the CNS, it was withdrawn from the market in the year 2008^22^. Endocannabinoids control the neurochemical balance between GABA and glutamate, hence blockage of CB1 receptors may disturb this set point, resulting in imbalance of neurotransmitter activity. Blocking of CB1 receptors may increase GABA and glutamate release which may enhance anxiety as a side effect^6,23,24^. Anandamide activates two receptors i.e. CB1 receptors and vanilloid (TRPV1) receptors. When CB1 receptors are blocked, anandamide would activate TRPV1 receptors causing aversive reactions^24,25^. In brain, CB1 receptors are expressed on serotonergic neurons which play active role in both anxiety and depression. CB1 receptor antagonists stimulate the serotonin release and activate 5-HT_2C_ receptors which lead to anxiety^6^. As, CB1 receptors are present on terminals of GABA-ergic inhibitory neuronals, terminals of glutamatergic and serotonergic neurons, blocking of CB1 receptors inhibits dopaminergic neurons and activates 5-HT_2A/2C_ receptors resulting in anhedonia associated with depression^6^.

Previous reports have revealed that blockage of peripheral cannabinoid CB1 receptors can control food intake adequately. The modulation of feeding behavior by rimonabant is not attributed solely to the blockage of cannabinoid CB1 receptors present in neural circuit, but also due to the blockage of peripheral cannabinoid CB1 receptors distributed in liver, muscle, adipocytes and cells of pancreas^15,26–29^. CB1 receptors found on nerve terminals innervating the gastrointestinal tract^30,31^, are known to be involved in mediating satiety signals that originate in the gut^32^. The anorectic properties are a result of peripheral blockade of anandamine an endogenous cannabinoid mediating orexigenic signals that activate the sensory terminals of CB1R-expressing sensory neurons innervating the gut^26^. Recent studies also indicate that hypophagic effects of peripheral CB1 receptor blockade is mediated by controlling endogenous leptin that reaches to hypothalamic receptors to regulate hypothalamic anandamide levels and central endocannabinoid system^33^.

Blocking of central CB1 receptors could play an important role in the management of obesity but such a blockage produced serious psychiatric side effects. Restricting the permeability of CB1 receptor antagonists into the brain can restrict such CNS side effects^15^. After the withdrawal of rimonabant, a new strategy was adopted by the researchers to avoid adverse psychiatric effects by designing less hydrophobic more polar molecules which would fail to penetrate the blood brain barrier (BBB) and remain localized in the peripheral tissues^3^. Previous studies have revealed that peripheral cannabinoid CB1 receptor blockage is sufficient enough to suppress food intake^26^, increase energy expenditure and reduce lipogenesis in both liver and adipose tissues^27,28^. A number of scaffolds especially diaryl heterocyclic compounds such as pyrazoles^34–38^, pyrroles^39,40^, pyrazolines^41,42^, purines^43,44^, tetrahydroindazoles^45^, tetrahydropyrazolo[4,3-*c*]pyridines^46^ etc. have been tried as CB1 receptor antagonists acting peripherally with lower CNS side effects but, unfortunately none of these compounds could make it to the market till date. So, there is an urgent need to discover newer scaffolds having peripherally restricted CB1 receptor antagonistic activity with minimum or no CNS effects. The polar surface area (PSA) of a compound i.e. the total surface area of polar atoms like O and N present in the structure of the compound, along with its hydrophobicity govern the passive diffusion of a molecule through BBB^47^. Molecules with a PSA value of less than 60 Å^2^ can easily penetrate the BBB, thus a higher PSA value is the foremost requirement for peripherally restricted activity^42,47,48^. This strategy gathered enormous attention for the designing of peripherally active compounds. Compounds with relatively higher PSA and lower hydrophobicity are expected to fail penetration into the CNS through BBB, so the CNS side effects could be completely avoided^36,42^. Peripherally restricted CB1 receptor antagonists can be designed by introducing polar functional groups in the molecules thereby increasing the PSA of the resulting compounds.

**Figure 1:**
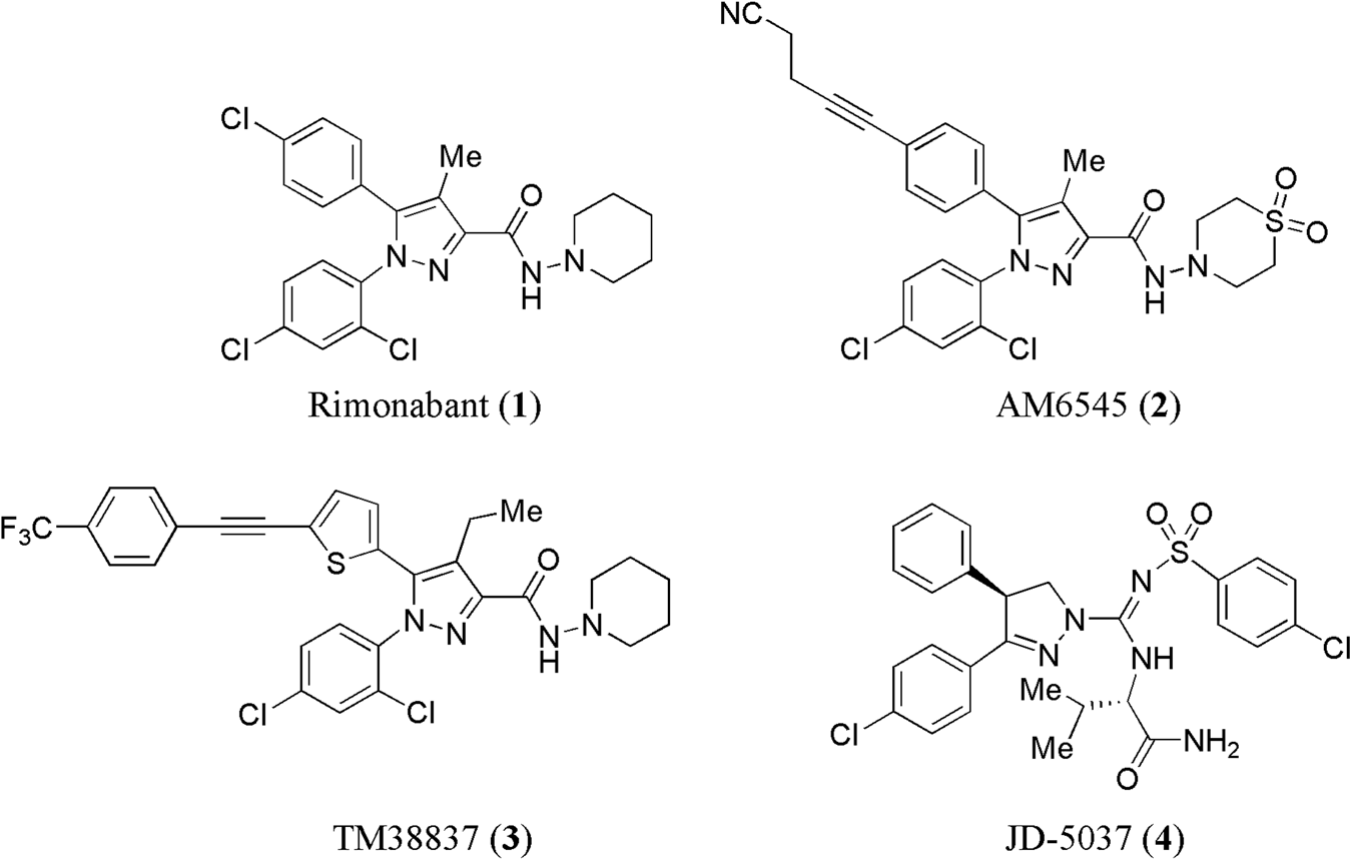
Chemical structures of some known centrally and peripherally acting CB1 receptor antagonists (**1**–**4**).

Some peripherally acting selective CB1 antagonists such as AM-6545 (**2**)^49^ synthesized at Center for Drug Discovery, Northeastern University, in the Alexander Makriyannis lab showed limited brain penetration with less neuropsychiatric side effects. 7TM Pharma reported TM38837 (**3**)^50^ having low brain-to-plasma ratio (1:33) and potent CB1R antagonist activity (EC_50_ = 8.5 nM). Another compound JD-5037 (**4**)^42^ from Jenrin Discovery has successfully completed preclinical evaluation and the company is planning to file an investigational new drug (IND) application for it in 2017 as a peripherally acting CB1 receptor antagonist (Fig. 1).

**Figure 2:**
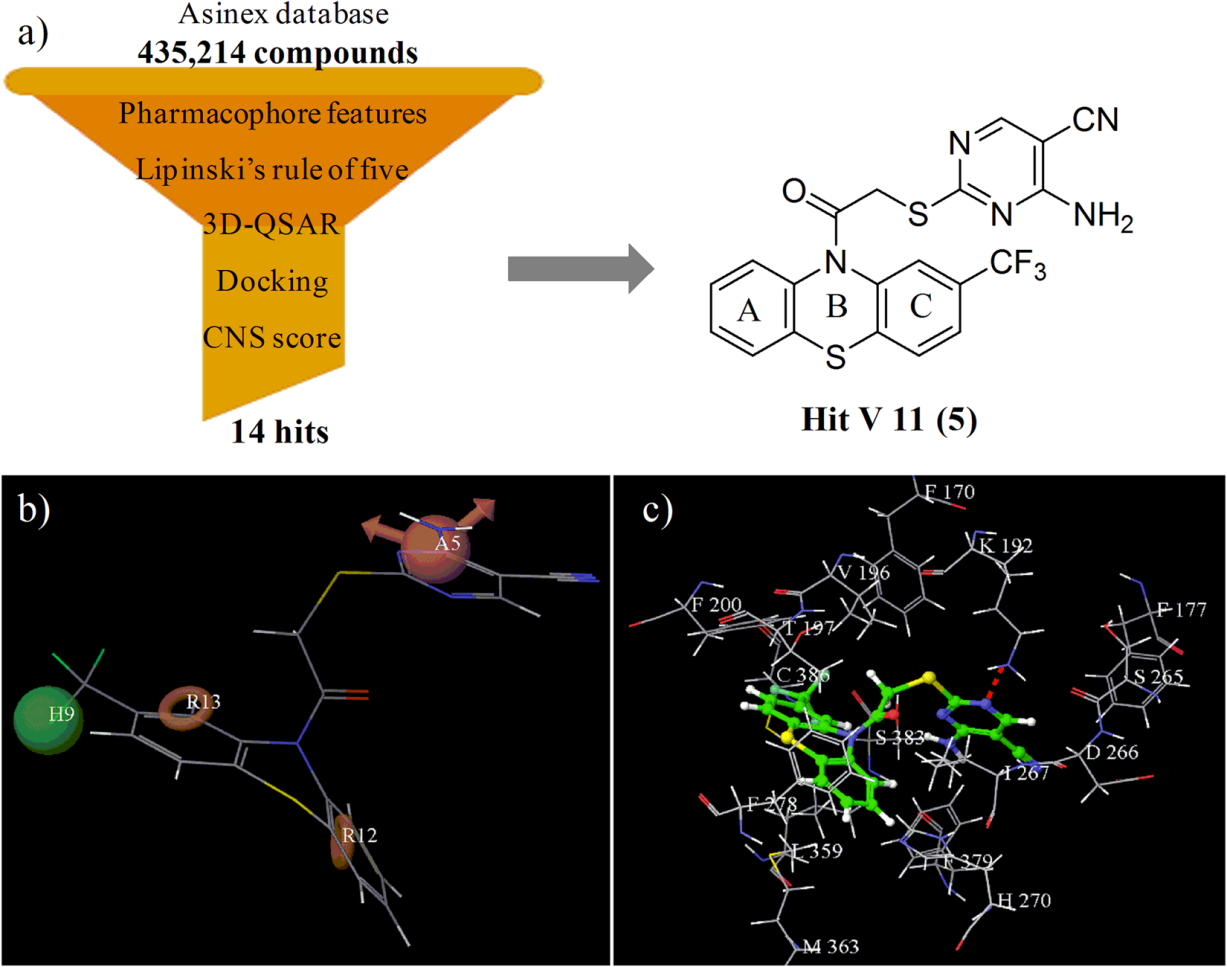
(**a**) Virtual screening flowchart and chemical structure of hit V11 (**5**), **(b**) Pharmacophore model (AHRR) aligned to hit V11 (**5**), (**c**) Binding mode of hit V11 (**5**) in the active site of CB1 receptor (red dotted line represents hydrogen bond).

Our group has been actively engaged in the design and development of novel peripherally acting CB1 receptor antagonists^3,13,51,52^. In the present work, we are reporting a novel series of compounds possessing phenothiazine scaffold as potential peripherally acting CB1 receptor antagonists. In the literature, compounds bearing phenothiazine scaffold have been reported to show different activities such as antipsychotic, anticonvulsant, antihistaminic, anticholinergic, antipruritic, antiemetic, antifungal, antibacterial, antiviral, anticancer, antimalarial, analgesic, anti-inflammatory, immunosuppressive, antifilarial, trypanocidal, and multidrug resistance reversal properties^53–55^. Probably this is the first report in which compounds with phenothiazine scaffold have been reported as peripherally acting CB1 receptor antagonists. The selection of phenothiazine scaffold was based on our previous communication^52^ wherein we reported 14 hits obtained through molecular modeling studies. To our surprise, out of these 14 hits, one was having phenothiazine scaffold, which was quite unexpected as there were no reports on phenothiazine scaffold-containing compounds as CB1 receptor antagonists. The literature is flooded with monotonous reports^3,13,34–46^ on vicinal diaryl heterocyclic systems exhibiting CB1 receptor antagonizing properties, and any other new chemical framework showing such an activity has the potential to give the drug discovery program on CB1 receptor antagonists, a new lease of life. Thus, it was planned to take up the synthesis of this novel hit 2-(2-(2-(trifluoromethyl)-10*H*-phenothiazin-10-yl)-2-oxoethylthio)-4-aminopyrimidine-5-carbonitrile (V11) (Fig. 2) and evaluate it biologically. The hit (V11) (compound **5**) so obtained was theoretically optimized by substituting different hydrophobic and hydrophilic functional groups, in light of the inputs obtained from the previously developed pharmacophore model and atom-based three dimensional quantitative structure–activity relationship (3D-QSAR) model. Thus, a series of phenothiazine derivatives were designed and synthesized which offered encouraging results in molecular modeling studies, in parallel artificial membrane permeability assay (PAMPA) and preliminary *in vivo* animal studies.

## Results and Discussion

### Identification of hit (V11) as anti-obesity agent

In the current era of drug discovery process, computational methods play a vital role in the identification of novel hits, which could be optimized further into clinically useful therapeutic agents. In our search for newer scaffolds as CB1 receptor antagonist, we successfully reported^52^ new hits by applying ‘virtual screening technique’ on Asinex database (containing 435,214 compounds) using different filters and tools such as pharmacophore map, 3D-QSAR model, Lipinski's rule of five, CNS scoring and receptor-ligand interaction studies. Out of the initial 435,214 compounds, 14 compounds were finally identified as novel hits. Among these 14 hits, seven hits (V1, V4, V7, V8, V11, V12 and V14) were found to possess new scaffolds, which were never reported as CB1 receptor antagonists. Except for the hit (V11), the remaining six hits were having quite similar-type of scaffolds containing diaryl rings. The hit (V11) contained phenothiazine scaffold which has never been reported in the literature exhibiting CB1 receptor antagonist activity.

The hit (V11) was evaluated qualitatively using our previously developed pharmacophore model (AHRR) for peripherally acting CB1R antagonists. In the four featured pharmacophore (AHRR) model, the two phenyl rings of the phenothiazine scaffold of the hit (V11) occupied the aromatic ring (R12 and R13) features. The hydrophobic (H9) feature was occupied by the trifluoromethyl group attached to 2^nd^ position of the phenothiazine ring. The hydrogen bond acceptor (A5) feature was occupied by one of the nitrogen atoms of the pyrimidine ring indicating that the hit (V11) fitted well into the pharmacophore model as shown in Fig. 2b. The *in silico* activity of the obtained hit (V11) was computed by using the previously developed atom-based 3D-QSAR model^52^ offering a *p*K_i_ value of 7.55. The threshold value for the active compounds was set to be *p*Ki ≥ 7.5 in the model indicating that the hit (V11) could be an important true positive. Orientation of the hit (V11) in the active site of CB1 receptor was also studied and was found to be quite similar to rimonabant (Fig. 2c). The trifluoromethyl group attached to the 2^nd^ position of the phenothiazine ring of the hit (V11) was oriented towards Val196, Phe200, Trp356 and Leu360 residues similar to the 4-chlorophenyl group of rimonabant. Ring A of the phenothiazine scaffold of the hit (V11) was oriented towards Trp279 and Met363 residues similar to the rimonabant's 2,4-dichlorophenyl ring. The pyrimidine ring of the hit (V11) was oriented towards Phe177, Phe189 and Trp255 residues similar to the piperidyl ring of rimonabant. The hit (V11) made a hydrogen bond attachment between the nitrogen of pyrimidine ring and Lys192, which was considered to be essential for exhibiting CB1 receptor antagonistic activity. Thus, the docking studies indicated that the hit (V11) was having an orientation similar to rimonabant in the active site of the CB1 receptor as shown in Fig. 2c. The results of the pharmacophore mapping, 3D-QSAR and docking studies clearly indicated that the hit (V11) was an appropriate case for further structural optimization. The result of biological evaluation of the hit (V11) (compound **5**) as an orally active CB1 receptor antagonist supported the theoretical studies. So, it was planned to go ahead with the synthesis and optimization of a novel series of phenothiazines as peripherally acting CB1 receptor antagonist for the treatment of obesity.

### Optimization of the hit V11 (5)

CB1 receptor antagonists are believed to be potent anti-obesity agents. But, their clinical application is limited because of their associated central side effects. To overcome their CNS side effects, the clinical candidates must have restricted entry into the brain. Therefore, the current drug designing approach was aimed at developing peripherally active compounds only, as successful anti-obesity agents.

In order to enhance the potency and PSA, and decrease the hydrophobicity, *in silico* modifications were carried out in the structure of the hit (V11) molecules, and the resulting compounds were evaluated initially using molecular modeling techniques. In accordance to the pharmacophore (AHRR) model, two phenyl rings of the phenothiazine scaffold of the hit (V11) were perfectly aligned on the pharmacophoric ring features (R12 and R13), hence it was decided not to temper with the phenyl rings of the phenothiazine scaffold in the new molecules to be designed. Modifications were envisaged on the other two pharmacophoric sites in the molecule i.e. on the sites having the hydrophobic feature and the hydrogen bond acceptor feature. The hydrophobic (H) feature of the pharmacophore was occupied by the trifluoromethyl group of the hit (V11). So, different hydrophobic substituents such as trifluoromethyl, chloro and methoxy were planned to be attached, at this position and the activity of the resulting compounds was predicted. Keeping hydrogen at this position decreased the activity as was found later on in case of compound (**24**) because the molecule complied only with three pharmacophoric features (ARR). In the pharmacophoric region having the hydrogen bond acceptor (A) group, it was decided to attach some polar functional groups so that these groups could occupy the hydrogen bond acceptor region that would show mandatory interaction with the Lys192 residue in the active site, a key amino acid residue for CB1 receptor binding. Thus, considering the synthetic feasibility, *in silico* assessment of compounds having polar functional groups such as 4,5-dihydrothiazole, 1-phenyl-1*H*-tetrazole, 1,3,4-thiadiazol-2-amine, 4-(1,3,4-oxadiazol-2-yl)pyridine, 4-amino-5-(4-pyridyl)-4*H*-1,2,4-triazole and ethyl 4-aminopyrimidine-5-carboxylate, was done. The 4,5-dihydrothiazole substituted compound showed low PSA and G-score values of 33.15 Å^2^ and -8.58 respectively in the *in silico* studies. In 1-phenyl-1*H*-tetrazole substituted compound, the PSA increased to 69.66 Å^2^ but the G-score got affected adversely (−7.44). The 1,3,4-thiadiazol-2-amino substituted compound offered comparatively better values of PSA and G-score i.e. 74.10 Å^2^ and −8.85 respectively. 4-(1,3,4-Oxadiazol-2-yl)pyridine substituted compounds gave improved PSA value of 76.81 Å^2^ and G-score of −10.79. Substitution with 4-amino-5-(4-pyridyl)-4*H*-1,2,4-triazole group increased the PSA value to 96.27 Å^2^ and improved the G-score further to −11.74. The ethyl 4-aminopyrimidine-5-carboxylate substituted compound showed the highest PSA value of 106.26 Å^2^ with a good G-score of −10.37. Higher values of PSA and more negative G-scores in the docking experiments suggested that the substituted pyrimidine, oxadiazole and triazole groups could prove to be favourable for peripheral CB1R antagonist activity. Furthermore, good fitness scores and improved predictive activities were obtained for these compounds in the pharmacophore modeling and atom-based 3D-QSAR studies respectively; therefore they were selected for chemical synthesis.

Efforts were also made to vary the length of the carbon chain spacer between the phenothiazine scaffold and the H-bond acceptor group like 4-amino-2-mercaptopyrimidine-5-carbonitrile. The two carbon chain-bearing compounds yielded poor results (fitness score = 1.09, *p*Ki = 6.90, G score = −9.17) in comparison to the compounds having three carbon spacer (fitness score = 1.15, *p*Ki = 8.20, G score = −10.17). So, it was decided to synthesize some compounds having three carbon-chain spacer also. The fitness score, predicted activity (*p*K_i_) and G-scores of the designed compounds are shown in Table 1.

**Table 1:** Fitness score, predicted activity and G-score of the synthesized compounds.

Comp	Fitness Score (Pharmacophore)	Predicted Activity (*p*K_i_) (3D-QSAR model)	G-score (Docking)
**5**	1.55	7.55	−10.58
**22**	1.48	7.59	−10.05
**23**	1.63	7.66	−10.04
**24**	1.76	7.04	−10.21
**25**	1.53	7.99	−10.37
**26**	1.77	7.52	−9.46
**27**	1.60	8.00	−10.33
**28**	1.72	6.99	−10.35
**29**	1.67	7.16	−10.79
**30**	1.69	7.31	−9.48
**31**	1.67	7.47	−9.10
**32**	1.14	7.51	−9.99
**33**	1.45	7.60	−11.74
**34**	1.46	7.75	−9.80
**35**	1.44	7.17	−10.70
**36**	0.95	7.10	−9.49
**42**	1.64	7.55	−12.59
**43**	1.15	8.20	−10.17
**44**	1.76	8.15	−10.34
**45**	1.64	7.12	−10.24
**46**	1.63	8.15	−11.20
**47**	1.55	7.79	−8.70
**48**	1.42	7.11	−9.22
**49**	1.63	7.29	−9.57
**Rimonabant**	2.53	7.90	−9.01

So, the identified hit (V11) was modified by incorporating different hydrophobic and polar functional groups in its structure. The compounds planned for the synthesis contained groups such as trifluoromethyl, chloro, methoxy and hydrogen, at 2^nd^ position of the phenothiazine ring and polar functional groups such as substituted pyrimidine, oxadiazole and triazole attached to the amide chain. Some compounds having a spacer of three carbon chain in between the phenothiazine scaffold and the polar pyrimidine ring were also planned to be synthesized. All the designed compounds were evaluated *in silico* using the pharmacophore model, 3D-QSAR model and docking studies, prior to their synthesis, as shown in Table 1.

The physicochemical and pharmacokinetic parameters of the designed compounds were also calculated. Physicochemical properties such as molecular weight (391.46–506.56 Da), number of hydrogen bond donors (0–2), number of hydrogen bond acceptors (6–9), hydrophobicity (2.77–6.31), polar surface area (75.22–114.55) and number of rotatable bonds (3–9) were all in the acceptable range. All of the designed compounds were theoretically predicted to show good absorption (71.62–100%) in humans. BBB permeability, an important parameter for peripherally acting CB1 antagonists, was also predicted for the designed compounds. The threshold value for the compounds which could readily cross the BBB was logBB > 0.3 whereas logBB < −1 indicated poor penetration into the brain^56^. The computed values of BBB permeability of the designed compounds were in the range −0.52 to −1.72, whereas the BBB permeability value for rimonabant was calculated to be 0.23. Based upon this data, it was expected that the designed compounds would not cross the BBB. It was also predicted that all the compounds would be metabolized by CYP3A4 enzyme system. The total clearance of the synthesized compounds, ranging from 0.009–0.351 ml/min/kg, indicated good physicochemical and pharmacokinetic properties (Table 2) suggesting their drug-like behavior.

**Table 2:** Calculated physicochemical and pharmacokinetic properties of the synthesized compounds.

Comp	Mol. Wt	No. of HBD	No. of HBA	log P	PSA	No. of rotatable bonds	% Absorp-tion (human)	BBB perme-ability (logBB)	Metabolizedby	Total clearance log(ml/min/kg)
**5**	459.46	2	7	3.77	100.37	5	89.93	−1.30	CYP3A4	0.158
**22**	425.91	2	7	3.59	99.42	5	88.59	−1.02	CYP3A4	0.079
**23**	421.49	2	8	3.20	107.71	6	86.29	−0.97	CYP3A4	0.313
**24**	391.46	2	7	2.95	97.59	5	86.93	−0.84	CYP3A4	0.138
**25**	506.51	2	8	5.33	106.26	6	78.15	−1.54	CYP3A4	0.195
**26**	472.96	2	8	4.83	106.27	6	100.00	−1.26	CYP3A4	0.117
**27**	468.54	2	8	4.42	114.55	7	100.00	−1.31	CYP3A4	0.351
**28**	438.51	2	8	4.42	104.43	6	100.00	−1.08	CYP3A4	0.176
**29**	486.48	0	7	4.68	76.81	3	100.00	−1.57	CYP3A4	0.150
**30**	452.93	0	8	4.17	76.82	3	100.00	−0.89	CYP3A4	0.067
**31**	448.51	0	8	3.72	85.10	4	100.00	−1.32	CYP3A4	0.302
**32**	418.48	0	7	3.66	76.83	3	100.00	−1.10	CYP3A4	0.133
**33**	500.51	2	8	3.73	96.27	4	71.62	−1.72	CYP3A4	0.032
**34**	466.96	2	8	3.25	96.28	4	81.62	−1.44	CYP3A4	0.046
**35**	462.54	2	9	2.85	104.57	5	79.30	−1.09	CYP3A4	0.174
**36**	432.51	2	8	2.77	96.29	4	78.80	−0.87	CYP3A4	0.009
**42**	459.50	2	6	5.04	75.23	7	91.35	−0.95	CYP3A4	0.151
**43**	425.93	2	6	4.33	75.33	7	100.00	−0.68	CYP3A4	0.094
**44**	421.53	2	7	4.17	81.92	8	100.00	−0.74	CYP3A4	0.319
**45**	391.50	2	6	4.07	75.22	7	100.00	−0.52	CYP3A4	0.153
**46**	506.56	2	7	6.31	81.73	8	89.36	−1.25	CYP3A4	0.160
**47**	473.00	2	7	5.82	81.73	8	100.00	−0.98	CYP3A4	0.103
**48**	468.58	2	8	5.47	87.55	9	100.00	−1.04	CYP3A4	0.328
**49**	438.56	2	7	5.34	80.52	8	95.59	−0.81	CYP3A4	0.162
**Rimonabant**	463.79	1	5	6.11	53.32	2	100.00	0.23	CYP3A4	0.156

### Chemistry

**Figure 3:**
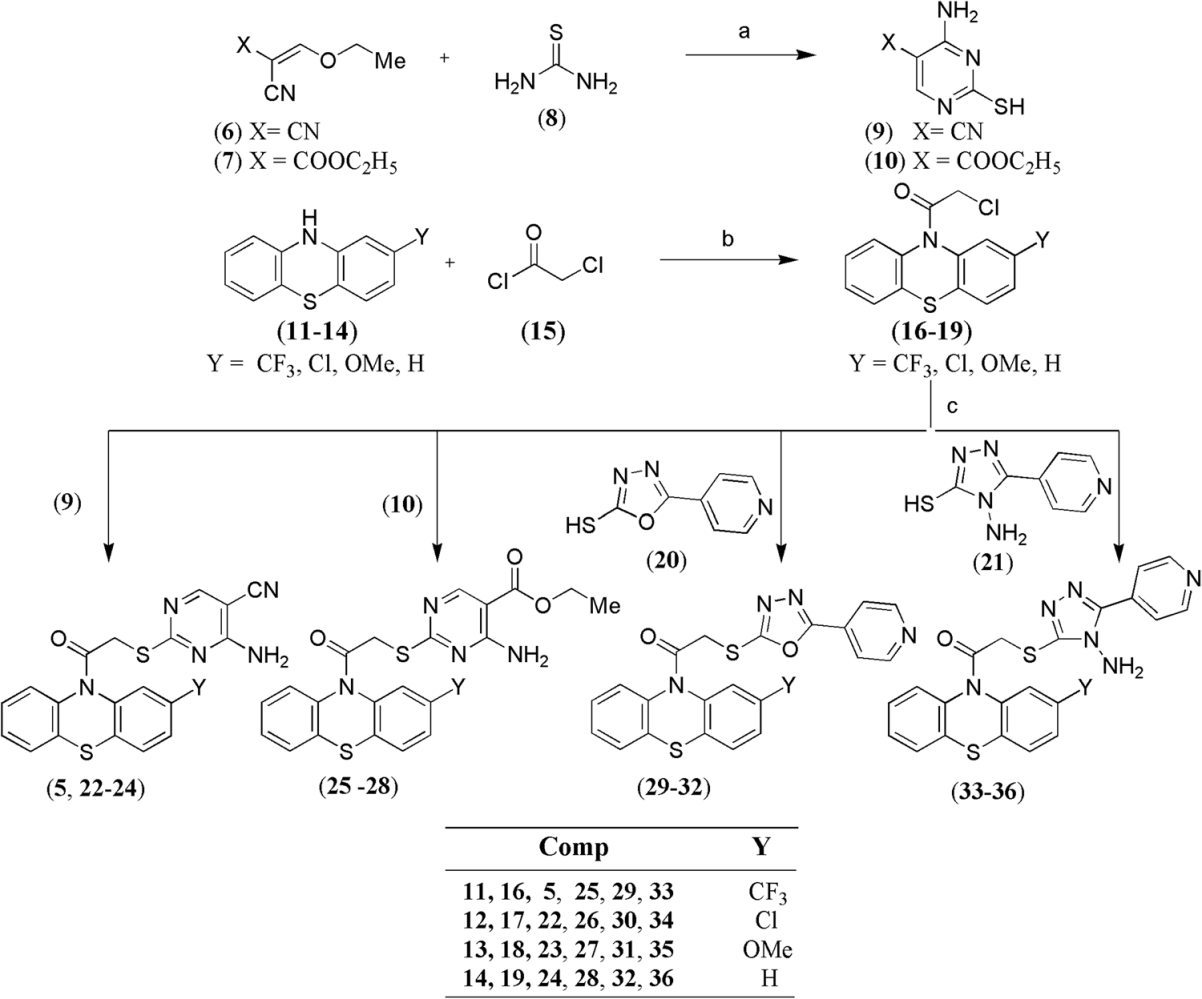
Synthetic pathway for compounds (**5**, **22**–**36**). Reagents and conditions: (**a**) NaOC_2_H_5_, EtOH, rt; (**b**) triethylamine, DCM, reflux; (**c**) K_2_CO_3_, DMF, rt.

**Figure 4:**
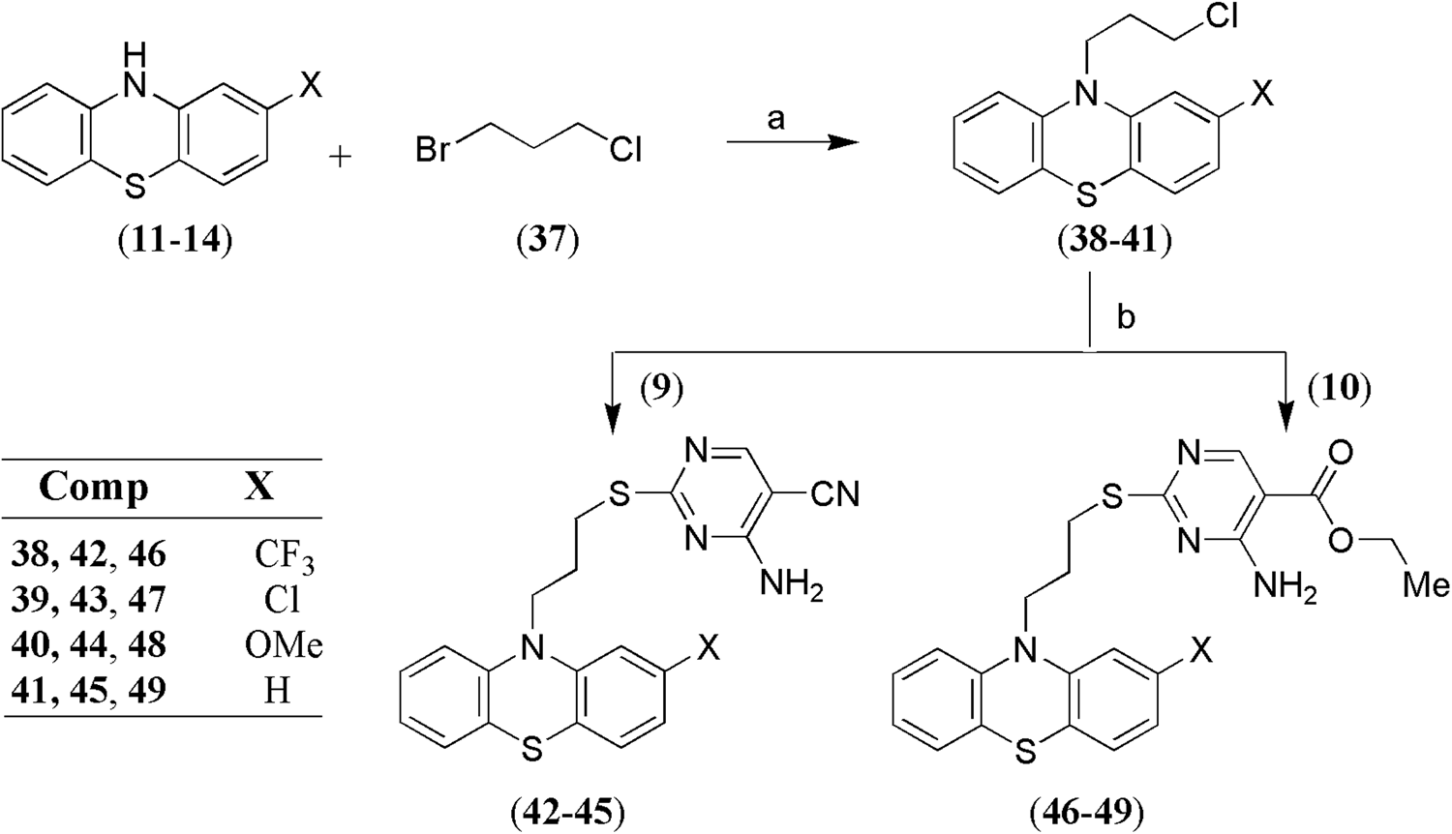
Synthetic pathway for compounds (**42**–**49**). Reagents and conditions: (**a**) NaH, DMSO, THF, 0 °C; (**b**) K_2_CO_3_, DMF, rt.

The designed compounds were synthesized as per the synthetic schemes shown in Figs 3 and 4. In the synthetic scheme (Fig. 3), ethoxymethylenemalononitrile and ethyl 2-cyano-3-ethoxyacrylate (**6** and **7**) were treated with thiourea (**8**) in the presence of sodium ethoxide as base at room temperature to obtain 5-substituted 4-amino-2-mercaptopyrimidine derivatives (**9** and **10**) respectively. The 2-substituted phenothiazine derivatives (**11**–**14**) were treated with chloroacetyl chloride (**15**) using triethylamine as base to yield the 2-chloro-1-(10*H*-phenothiazin-10-yl)ethanone derivatives (**16**–**19**) which were further treated with the thiol derivatives (**9**, **10, 20, 21**) using potassium carbonate and dimethylformamide (DMF) to obtain the final compounds (**5**, **22**–**36**).

In the synthetic scheme (Fig. 4), 2-substituted phenothiazine derivatives (**11**–**14**) were treated with 1-bromo-3-chloropropane (**37**) using sodium hydride in dimethyl sulfoxide (DMSO) and tetrahydrofuran (THF) to yield 10-(3-chloropropyl)-10*H*-phenothiazine derivatives (**38**–**41**), which were further reacted with the thiol derivatives (**9**, **10**) to form the desired compounds (**42**–**49**).

### Biological

#### *In vitro* permeability assay of the test compounds

As discussed above, BBB permeation of the available CB1 receptor antagonists limits their therapeutic utility. A successful CB1 receptor antagonist to be used as anti-obesity agent must be devoid of BBB permeability. Compounds which showed predicted activity (*p*Ki) of ≥7.5 were selected for evaluation for their tendency to penetrate the BBB. In this study, the ability of the test compounds to cross the BBB was determined by the well established *in vitro* model PAMPA-BBB assay. This simple and rapid model has the advantage to predict passive BBB permeation with high accuracy^57,58^. The *in vitro* permeability (*P*_e_) of the test compounds through the lipid extract of porcine brain was determined in PBS/ethanol (70:30). All the test compounds showed permeability (*P*_e_) values lesser than the threshold value of 3.8 × 10^–6^ cm s^−1^, suggesting^58^ that they would have nil/low propensity to cross the BBB by passive diffusion (Table 3).

**Table 3:** Permeability (*P*_e_) results of the test compounds from the PAMPA-BBB assay with their predicted penetration in CNS. (Data expressed as mean ± SEM of three independent experiments. CNS- indicates low passive CNS permeation).

Compound	*P*_*e*_ (10^–6^ cm s^−1^)	Prediction
**5**	2.78 ± 0.14	CNS-
**22**	2.28 ± 0.11	CNS-
**23**	3.08 ± 0.16	CNS-
**25**	2.72 ± 0.19	CNS-
**26**	3.66 ± 0.67	CNS-
**27**	2.80 ± 0.58	CNS-
**32**	3.22 ± 0.63	CNS-
**33**	1.05 ± 0.22	CNS-
**34**	2.09 ± 0.82	CNS-
**42**	2.60 ± 0.18	CNS-
**43**	3.47 ± 0.66	CNS-
**44**	3.58 ± 0.73	CNS-
**46**	2.84 ± 0.28	CNS-
**47**	3.28 ± 0.38	CNS-

#### Acute hypophagic effect of the test compounds

**Figure 5:**
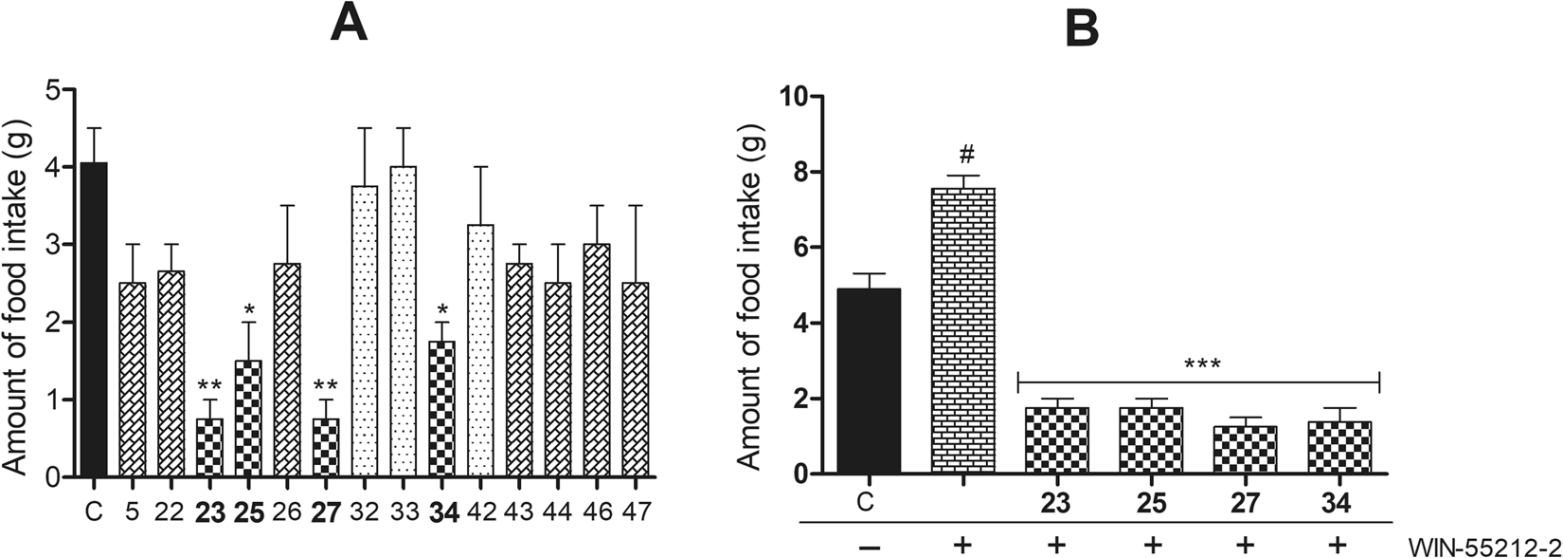
Hypophagic response of the test compounds in twenty four hour fasted rats. Among the tested compounds, **23**, **25**, **27** and **34** (10 mg/kg, p.o.) showed significant hypophagic activity alone (**A**) and also suppressed the hyperphagic effect of WIN-55212-2 (2 mg/kg, i.p.) (**B**) revealing their CB1 receptor antagonist potential. Data expressed as mean ± SEM (n = 6). **p < 0.01, *p < 0.05 *vs*. vehicle-treated control group (**A**). ^#^*p* < 0.01 *vs*. vehicle-treated control group. ***p < 0.001 *vs*. WIN-55212-2-treated group (**B**). One-way ANOVA, Bonferroni *post hoc* test.

As a preliminary screening, the synthesized compounds were evaluated for acute hypophagia in rats and the results are summarized in Fig. 5. Twenty four hour food deprived Wistar rats were given weighed amounts of food pellets after administration of the test compounds. Quantum of food consumed by the control group of animals was considered as 100%. Out of the 14 compounds evaluated for this activity, three compounds (**32**, **33** and **42**) were found inactive as they caused insignificant decrease in the food intake (80–99% food intake), while seven compounds (**5, 22, 26, 43**, **44**, **46** and **47**) were found weakly active (62–74%) causing mild acute hypophagia after 2 h of treatment. However, four compounds (**23, 25, 27** and **34**) showed significant hypophagic activity (19–43%) as compared to the control animals (*p* < 0.01) (Fig. 5A).

In another set of experiment, the selected test compounds (**23, 25, 27** and **34**) were further evaluated to assess their *in vivo* CB1 receptor antagonistic activity in presence of WIN-55212–2, a potent cannabinoid receptor agonist. Here also, the quantity of feed consumed by the control group animals was taken as 100%. The animals treated with the CB1R agonist WIN-55212-2, consumed 154% feed in comparison to the control group animals. Ability of the test compounds to act as CB1 receptor antagonists was assessed by their capability to attenuate the hyperphagic response shown by the animals treated with WIN-55212-2. WIN-55212-2 significantly increased (54%) the amount of food intake in treated animals as compared to the control group (*p* < 0.05). However, the hyperphagic effect of WIN-55212-2 was significantly attenuated by the test compounds (**23, 25, 27** and **34**) (*p* < 0.001). These compounds (**23**, **25**, **27** and **34**) showed significant decrease (36%, 36%, 26% and 28%, respectively) in food intake as compared to the control. The results supported CB1 receptor antagonistic potential of the test compounds (**23, 25, 27** and **34**) (Fig. 5B).

### Molecular modeling studies and SAR of the synthesized compounds

#### Pharmacophore mapping

**Figure 6:**
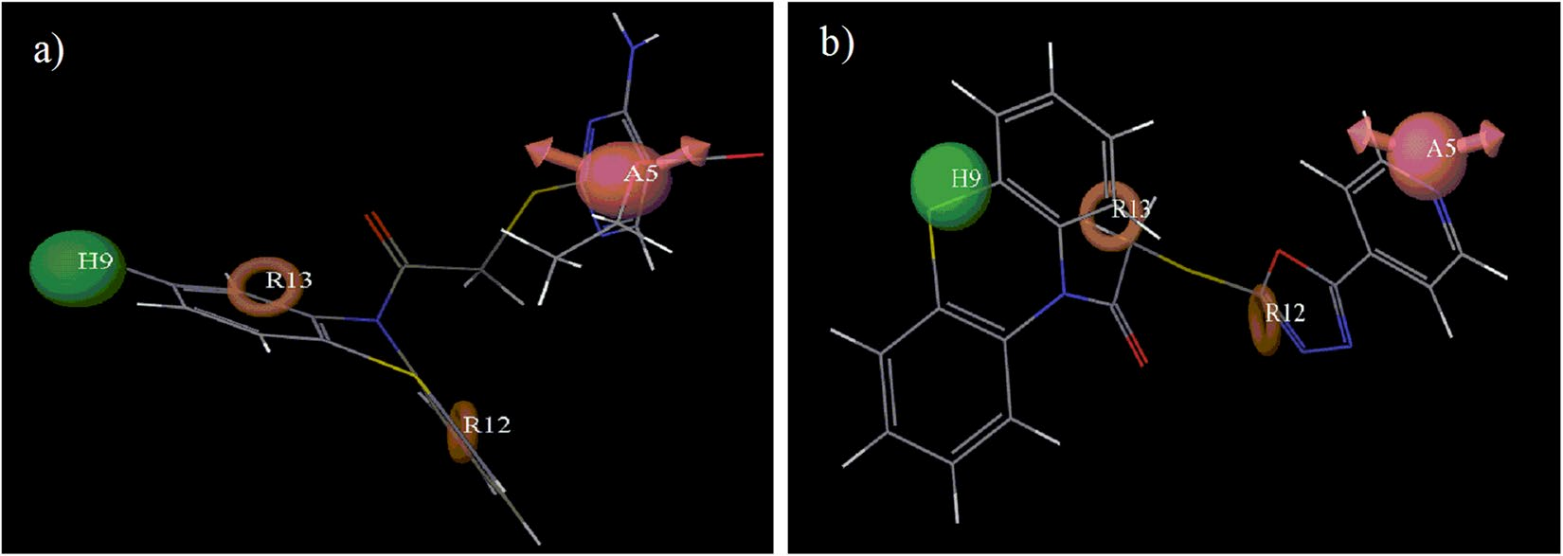
Mapping of (**a**) the highest fitness score compound (**26**) and (**b**) the lowest fitness score compound (**32**) on the best pharmacophoric model (AHRR).

Modeling studies were performed to evaluate the structural fitting of the synthesized compounds into the previously developed pharmacophore model (AHRR)^52^. The highest fitness score was observed (1.77) for compound (**26**) in pharmacophore mapping. Compound (**26**) occupied all four of the pharmacophoric features as shown in Fig. 6a in which two aromatic rings of the phenothiazine scaffold occupied the two aromatic ring features, Cl group at 2^nd^ position of the phenothiazine ring occupied the hydrophobic feature and oxygen of the ester group acted as the hydrogen bond acceptor group. The most active compounds (**23**, **25**, **27** and **34**) showed good fitness scores (1.63, 1.53, 1.60 and 1.46 respectively) and structures of all four compounds fitted well into all the four pharmacophoric features. Compound (**32**) showed a lowered fitness score of 1.14 as it did not contain a hydrophobic group at 2^nd^ position of the phenothiazine ring when mapped in the pharmacophore model. Absence of the hydrophobic group flipped the molecule in such a way that one of the aromatic features was occupied by the phenyl ring of the phenothiazine scaffold and the other one by the oxadiazole ring. Hydrogen bond acceptor feature was oriented near the nitrogen of the pyridine ring in the attached side chain as shown in Fig. 6b. So, the orientation of compound (**32**) got a lowered fitness score in the pharmacophore mapping. It became clear from this study that it was necessary for the phenothiazine derivatives to match all the pharmacophoric features for good biological activity.

#### Docking studies

**Figure 7:**
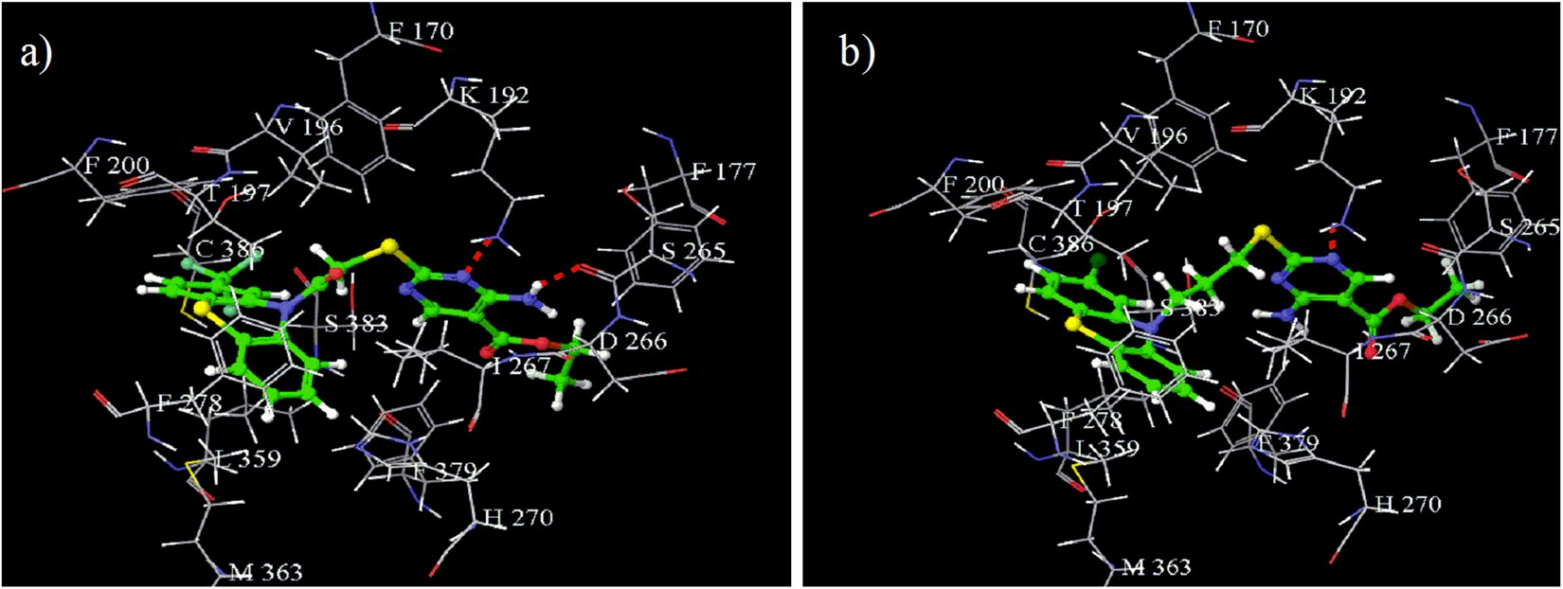
Binding interactions of (**a**) active compound (**25**), and (**b**) less active compound (**47**) in the active site of CB1 receptor (red dotted line represents hydrogen bond).

In docking studies, it was observed that formation of a hydrogen bond between Lys192 residue of the active site and the docked molecule was a characteristic feature for binding to the CB1 receptor. The virtual screening hit V11 (**5**) molecule having a G-score of −10.58 was observed to have the required binding in the active site of CB1 receptor with the Lys192 residue. Compounds (**25** and **27**) exhibiting G-scores of −10.37 and −10.33 respectively, formed two hydrogen bonds, one between one of the nitrogen atoms of the pyrimidine ring and the Lys192 residue, and another between the hydrogen of the amine attached to pyrimidine ring and the Ser265 residue, which might be responsible for their good biological activity. The orientation of compound (**25**) in the active site of CB1 receptor is shown in Fig. 7a. Similarly, compounds (**23** and **34**) also exhibited good G-scores (−10.04 and −9.80 respectively) and were suitably well oriented in the active site of CB1 receptor, which supported their good biological activity. The lowest G-score (−8.70) was obtained for compound (**47**). Although it also formed a hydrogen bond with Lys192 residue as shown in Fig. 7b but still showed a comparatively low G-score. This might be due to the presence of three carbon chain spacer which increased the distance between the phenothiazine scaffold (containing two aromatic rings and one hydrophobic features) and the substituted pyrimidine ring (containing a hydrogen bond acceptor feature).

#### Structure activity relationship (SAR)

Different phenothiazine derivatives were synthesized so that a preliminary structure activity relationship could be developed. For developing a robust SAR, a lot more number of compounds having different types of groups would be needed. In the acute hypophagic study, the food intake got decreased (62%) when the animals were treated with compound (**5**), the virtual screening hit (V11). Replacement of trifluoromethyl group of compound (**5**) with methoxy group yielded compound (**23**) which caused significant decrease in the food intake to 19% of the control. Replacement of 4-amino-2-mercaptopyrimidine-5-carbonitrile group of compound (**5**) by 4-aminopyrimidine-5-carboxylate resulted in compound (**25**) causing decrease in activity (food intake 37%). The trifluoromethyl group of compound (**25**) when replaced by methoxy group in compound (**27**) again caused a substantial improvement in the activity (food intake 19%). The trifluoromethyl and 4-amino-2-mercaptopyrimidine-5-carbonitrile groups of compound (**5**) when replaced by chloro and 4-amino-5-(4-pyridyl)-4*H*-1,2,4-triazole groups resulted in compound (**34**) having a lower activity (decreasing the food intake to 43%). Replacement of chloro group of **34** by trifluoromethyl group in compound (**33**) did not impact the food intake. Similarly, presence of hydrogen and 4-(1,3,4-oxadiazol-2-yl)pyridine groups in compound (**32**) also showed no effect on food intake. Removal of the amide linkage of compound (**5**) by three carbon methylene chain resulted in compound (**42**), again having no effect on the food intake. In another experiment carried out using WIN-55212-2 as CB1R agonist, compounds (**23**, **25**, **27** and **34**) showed significant decrease in food intake (36%, 36%, 26% and 28% respectively) which indicated their potential *in vivo* CB1 receptor antagonism.

In nutshell, the SAR revealed that the hydrophobic substituents such as trifluoromethyl, chloro and methoxy at 2^nd^ position of phenothiazine ring were beneficial for the activity, especially the methoxy group in compounds (**23** and **27**). Whereas, absence of hydrophobic feature at 2^nd^ position of the phenothiazine scaffold resulted in poor activity for the resulting compounds as observed in compound (**32**), underlining the importance of a hydrophobic feature at this position. Amide linkage in the spacer was observed to be favourable for the activity. Replacement of the amide group spacer by a three carbon methylene chain spacer was observed to offer comparatively less active compounds (**42**-**44**, **46** and **47**) than in comparison to the compounds (**23**, **25**, **27** and **34**) having an amide linkage. Different polar groups, such as 4-amino-2-mercaptopyrimidine-5-carbonitrile in compound (**23**), 4-aminopyrimidine-5-carboxylate in compounds (**25** and **27**) and 4-amino-5-(4-pyridyl)-4*H*-1,2,4-triazole group in compound (**34**), were found useful to increase the PSA of the resulting compounds and also acted as hydrogen bond acceptor features which formed hydrogen bond with the Lys192 residue providing favourable CB1R binding affinity to the ligands.

## Conclusion

With the aim to diversify the chemical domain of the existing peripherally acting CB1 antagonists, a novel hit possessing phenothiazine scaffold was identified. The hit molecule was structurally optimized to afford a novel series of phenothiazine derivatives having higher PSA and lower hydrophobicity as compared to rimonabant. Compounds of the designed series were synthesized. The synthesized compounds are hypothesized not to cross the blood brain barrier as assessed by PAMPA assay, indicating their peripherally restricted presence that could make them devoid of unwanted CNS side effects. In the preliminary screening, it was found that the hit V11 (**5**) possessed weak short acting hypophagic activity. Further modifications in the hit V11 (**5**) by using hydrophobic groups such as chloro and methoxy groups at 2^nd^ position of the phenothiazine ring were found beneficial for the activity in computational studies as well as whole animal assay. Substituted pyrimidine and triazole rings acted as hydrogen bond acceptors forming hydrogen bond with Lys192 residue, which was favourable for the activity. In the *in vivo* studies, the structurally modified compounds (**23, 25, 27** and **34**) showed a significant decrease in food intake in comparison to the control group. Thus, the phenothiazine moiety has been identified as a promising scaffold in providing peripherally acting CB1 receptor antagonists for the management of obesity with nil/minimum CNS side effects. To the best of our knowledge this is the first report wherein the phenothiazine scaffold has been shown to exhibit peripherally acting CB1 receptor antagonistic activity which could be exploited therapeutically for the management of obesity without affecting the psyche of the subject under treatment.

## Experimental

### Computational

All the computational studies reported in the present work were carried out using Maestro (version 9.0 and 9.4) software of Schrödinger, New York^59,60^. The previously built pharmacophore and atom-based 3D-QSAR models were used in this study^52^ for mapping of the pharmacophoric features of the designed compounds, and prediction of the biological activity. For molecular modeling purposes, chemical structures of all the compounds were sketched, cleaned up and minimized by using OPLS 2005 force field in the standard tool ‘Ligprep’ version 2.3^61^. Molecular docking studies for the designed compounds were performed by using standard tool ‘Glide’ version 5.5^62^. The co-crystallized structure for CB1 receptor (PDB code: 5TGZ^63^) was downloaded from RCSB Protein Data Bank. Protein preparation was done by all hydrogen atom addition and water molecules deletion. The hydrogen bond assignment was optimized by exhaustive sampling method and the obtained protein structure was energy minimized in presence of OPLS_2005 force field using Impref module in the standard option ‘protein preparation wizard’ of Maestro 9.0. The obtained protein structure was used for receptor grid generation^64^. The energy minimized structures were docked into the active site of CB1 receptor by using extra-precision (XP) mode. The docking protocol was validated by first knocking out the ligand from the co-crystallized structure and then re-docking of the ligand in the active site of the CB1 receptor and by observing the interactions. Physicochemical and pharmacokinetic parameters were calculated by using Qikprop module of Maestro 9.0^65^ and pkCSM^56^.

### Chemistry

All the solvents and reagents used in the synthesis were of analytical reagent grade commercially procured from Sigma-Aldrich, Spectrochem, S. d. fine chemicals or Avra chemicals. Purification of all the solvents and reagents were carried out by using general laboratory techniques before their use for synthesis of the desired compounds^66^. The melting points (mp) of the compounds were determined by using Veego make silicon oil bath-type melting point apparatus and are uncorrected. Thin layer chromatography (TLC) using silica gel pre-coated plates (60F_254_, Merck, 0.25 mm thickness) were employed to monitor the progress of the reactions. TLC plates were visualized with ultraviolet light (254 nm) or iodine vapors. The practical yields of the compounds reported here are un-optimized. The infrared spectra (IR) expressed in wave numbers in cm^**−**1^ were obtained using BRUKER ALPHA-T (Germany) FT-IR spectrophotometer where potassium bromide discs were used in the region of 4000–450 cm^−1^. ^1^H-NMR (400 MHz) spectra and ^13^C-NMR spectra were determined on Bruker Advance-II spectrometer in CDCl_3_ or DMSO-*d*_6_ solvents; the chemical shift has been expressed in parts per million (δ ppm) and coupling constant (*J*) in Hz. The mass spectra were taken either on Thermo Fisher mass spectrometer having EI ion source or Shimadzu LCMS2020 with APCI & ESI probes for the compounds. Chromatographic separations were carried out on columns using silica gel (100–200) as the adsorbent.

### 4-Amino-2-mercaptopyrimidine-5-carbonitrile (9)

**Method A**: Freshly cut sodium (0.19 g, 8.26 mmol) was added into absolute ethanol (5 mL) in cold conditions. To this solution, thiourea (**8**) (0.62 g, 8.15 mmol) was added at room temperature. When thiourea got dissolved, ethoxymethylenemalononitrile (**6**) (1 g, 8.19 mmol) in ethanol (5 mL) was added using dropping funnel to the above stirred mixture over a period of 2 h and completion of the reaction was monitored by thin layer chromatography (TLC). The solvent was evaporated, water (10 mL) added to the residue and 2 M hydrochloric acid (HCl) was added to make the solution acidic. The precipitates so obtained were filtered to obtain a yellow colored solid of compounds (**9**)^67^ (0.92 g, 74%); mp: >270 °C (Lit^67^: >270 °C); IR (KBr, cm^−1^): 3443, 3370, 3042, 2959, 2223, 1646; MS (m/z): 153.1 (M + 1)^+^.

### Ethyl 4-amino-2-mercaptopyrimidine-5-carboxylate (10)

Reaction of thiourea (**8**) (0.45 g, 5.91 mmol) and ethyl 2-cyano-3-ethoxyacrylate (**7**) (1 g, 5.91 mmol) under a set of conditions described in **Method A**, afforded a white solid compound (**10**) (0.9 g, 76%); mp: >270 °C (Lit^67^ >270 °C); IR (KBr, cm^−1^): 3551, 3387, 3063, 2976, 1705, 1637, 1064; MS (m/z): 199.7 (M + 1)^+^.

### 2-Chloro-1-(2-trifluoromethyl-10*H*-phenothiazin-10-yl)ethanone (16)

**Method B:** Commercially available 2-trifluoromethyl-10*H*-phenothiazine (**11**) (1 g, 3.75 mmol) was dissolved in dry toluene (8 mL) and triethylamine (1.12 g, 11.07 mmol) was added to it at room temperature and the solution was stirred for 10 minutes. Chloroacetyl chloride (**15**) (1.24 g, 10.98 mmol) in dry toluene (3 mL) was added drop wise into the reaction mixture at 0–5 °C. The reaction mixture was refluxed for 5 to 6 h and completion of the reaction was checked by TLC. On completion of the reaction, the solvent was removed, water added to the residue and the suspension extracted with ethyl acetate. Purification through silica gel column using pet ether: ethyl acetate (5%) resulted into the desired pure white solid compound (**16**)^68,69^ (0.9 g, 70%); mp: 110–112 °C (Lit^68^: 110–111 °C); IR (KBr, cm^−1^): 3004, 2951, 1690, 823, 752.

### 2-Chloro-1-(2-chloro-10*H*-phenothiazin-10-yl)ethanone (17)

Reaction of 2-chloro-10*H*-phenothiazine (**12**) (1 g, 4.28 mmol), triethylamine (1.30 g, 12.85 mmol) and chloroacetyl chloride (**15**) (l.44 g, 12.75 mmol) in dry dichloromethane (DCM) (10 mL) under a set of conditions described in **Method B**, afforded white solid compound (**17**) (1.08 g, 82%); mp: 117–119 °C (Lit^68^: 118–119 °C); IR (KBr, cm^−1^): 3065, 2947, 1682, 758.

### 2-Chloro-1-(2-methoxy-10*H*-phenothiazin-10-yl)ethanone (18)

Reaction of 2-methoxy-10*H*-phenothiazine (**13**) (1 g, 4.08 mmol), triethylamine (1.24 g, 12.25 mmol) and chloroacetyl chloride (**15**) (l.38 g, 12.22 mmol) in dry DCM (10 mL) under a set of conditions described in **Method B**, afforded white solid compound (**18**) (1.04 g, 78%); mp: 120–122 °C (Lit^70^: 123–124 °C); IR (KBr, cm^−1^): 2996, 2956, 1689, 745.

### 2-Chloro-1-(10*H*-phenothiazin-10-yl)ethanone (19)

Reaction of 10*H*-phenothiazine (**14**) (1 g, 5.02 mmol), triethylamine (1.52 g, 15.02 mmol) and chloroacetyl chloride (**15**) (1.68 g, 14.88 mmol) in dry DCM (10 mL) under a set of conditions described in **Method B**, afforded white solid compound (**19**) (1.1 g, 80%); mp: 110–112 °C (Lit^68^: 114–115 °C); IR (KBr, cm^−1^): 3067, 2951, 1693, 762.

### 4-Amino-2-(2-(2-trifluoromethyl-10*H*-phenothiazin-10-yl)-2-oxoethylthio)pyrimidine-5-carbonitrile (5)

**Method C:** 4-Amino-2-mercaptopyrimidine-5-carbonitrile (**9**) (0.27 g, 1.77 mmol) was dissolved in dry DMF (2 mL) and stirred at room temperature. Anhydrous potassium carbonate (0.52 g, 3.77 mmol) was added to the above solution. 2-Chloro-1-(2-trifluoromethyl-10*H*-phenothiazin-10-yl)ethanone (**16**) (0.5 g, 1.45 mmol) dissolved in dry DMF (2 mL) was added dropwise to the above solution. The reaction mixture was stirred for 3 to 4 h and completion of the reaction checked by TLC. On addition of water to the reaction mixture, a white precipitate was obtained. Purification through silica gel column using pet ether: ethyl acetate (30%) as eluent gave pure white solid of the desired product (**5**)^71^ (0.45 g, 67%); mp: 218–220 °C; IR (KBr) cm^−1^: 3402, 3321, 2216, 1684, 1631, 1573, 1542, 1375, 1331; ^1^H NMR: 8.25 (s, 1 H), 7.64 (s, 1 H), 7.54–7.52 (d, 1 H, *J* = 7.8), 7.50–7.48 (d, 1 H, *J* = 7.8), 7.40–7.36 (m, 2 H), 7.32–7.28 (m, 1 H), 7.26–7.23 (d, 1 H, *J* = 9.04), 5.70 (bs, 2 H), 4.03 (s, 2 H); ^13^C NMR: 172.88, 166.55, 161.22, 159.46, 138.12, 137.13, 131.35, 128.0, 127.79, 127.42, 127.19, 126.85, 124.67, 123.96, 123.0, 121.97, 114.91, 85.94, 34.02; MS (ESI) m/z: 460.2 (M + 1)^+^; Anal. Calcd for C_20_H_12_F_3_N_5_OS_2_: C, 52.28; H, 2.63; N, 15.24; Found: C, 52.40; H, 2.76; N, 15.12%.

### 4-Amino-2-(2-(2-chloro-10*H*-phenothiazin-10-yl)-2-oxoethylthio)pyrimidine-5-carbonitrile (22)

Reaction of 4-amino-2-mercaptopyrimidine-5-carbonitrile (**9**) (0.29 g, 1.91 mmol), anhydrous potassium carbonate (0.55 g, 3.99 mmol) and 2-chloro-1-(2-chloro-10*H*-phenothiazin-10-yl)ethanone (**17**) (0.5 g, 1.61 mmol) in dry DMF (4 mL) under a set of conditions described in **Method C**, afforded white solid compound (**22**) (0.49 g, 71%); mp: 222–224 °C; IR (KBr) cm^−1^: 3460, 3332, 2224, 1645, 1574, 1534, 1462, 1402, 1237; ^1^H NMR: 8.24 (s, 1 H), 7.91 (s, 1 H), 7.58–7.54 (d, 2 H), 7.51–7.49 (d, 2 H), 7.43–7.39 (m, 1 H), 7.35–7.31 (m, 1 H), 5.74 (bs, 2 H), 4.05 (s, 2 H); MS (ESI) m/z: 426 (M + 1)^+^, 428 (M + 3)^+^; Anal. Calcd for C_19_H_12_ClN_5_OS_2_: C, 53.58; H, 2.84; N, 16.44; Found: C, 53.72; H, 2.68; N, 16.30%.

### 4-Amino-2-(2-(2-methoxy-10*H*-phenothiazin-10-yl)-2-oxoethylthio)pyrimidine-5-carbonitrile (23)

Reaction of 4-amino-2-mercaptopyrimidine-5-carbonitrile (**9**) (0.29 g, 1.91 mmol), anhydrous potassium carbonate (0.55 g, 3.99 mmol) and 2-chloro-1-(2-methoxy-10*H*-phenothiazin-10-yl)ethanone (**18**) (0.5 g, 1.63 mmol) in dry DMF (4 mL) under a set of conditions described in **Method C**, afforded white solid compound (**23**) (0.48 g, 69%); mp: 215–217 °C; IR (cm^−1^): 3337, 3179, 2219, 1664, 1575, 1537, 1465, 1353, 1245, 1166; ^1^H NMR: 8.26 (s, 1 H), 7.70 (bs, 2 H), 7.65–7.63 (d, 1 H, *J* = 7.76 Hz) 7.51–7.49 (d, 1 H, *J* = 7.76 Hz), 7.40–7.38 (d, 1 H), 7.36–7.34 (d, 1 H), 7.30 (s, 1 H), 7.28–7.26 (m, 1 H), 6.90–6.87 (dd, 1 H, *J* = 8.7, 2.6 Hz), 4.18 (s, 2 H), 3.80 (s, 3 H); ^13^C NMR: 173.02, 166.35, 161.23, 160.07, 159.99, 158.73, 139.25, 138.03, 128.23, 127.64, 127.25, 126.80, 115.18, 113.27, 113.02, 85.83, 55.46, 34.25; MS (EI) m/z: 421.12 (M^+^); Anal. Calcd for C_20_H_15_N_5_O_2_S_2_: C, 56.99; H, 3.59; N, 16.62; Found: C, 56.80; H, 3.70; N, 16.72%.

### 4-Amino-2-(2-oxo-2-(10*H*-phenothiazin-10-yl)ethylthio)pyrimidine-5-carbonitrile (24)

Reaction of 4-amino-2-mercaptopyrimidine-5-carbonitrile (**9**) (0.33 g, 2.17 mmol), anhydrous potassium carbonate (0.62 g, 4.49 mmol) and 2-chloro-1-(10*H*-phenothiazin-10-yl)ethanone (**19**) (0.5 g, 1.81 mmol) in dry DMF (4 mL) under a set of conditions described in **Method C**, afforded white solid compound (**24**) (0.45 g, 63%); mp: 241–243 °C; IR (KBr) cm^−1^: 3361, 3145, 2228, 1663, 1572, 1466, 1351, 1237, 1165, 757; ^1^H NMR: 8.21 (s, 1 H), 7.66–7.64 (d, 2 H, *J* = 7.8 Hz), 7.51–7.49 (d, 2 H, *J* = 7.8 Hz), 7.48 (bs, 2 H), 7.40–7.36 (m, 2 H), 7.31–7.27 (m, 2 H) and 4.12 (s, 2 H); MS (ESI) m/z: 414.4 (M + Na)^+^; Anal. Calcd for C_19_H_13_N_5_OS_2_: C, 58.29; H, 3.35; N, 17.89; Found: C, 58.42; H, 3.22; N, 17.78%.

### Ethyl 4-amino-2-(2-(2-trifluoromethyl-10*H*-phenothiazin-10-yl)-2-oxoethylthio)-pyrimidine-5-carboxylate (25)

Reaction of ethyl 4-amino-2-mercaptopyrimidine-5-carboxylate (**10**) (0.36 g, 1.81 mmol), anhydrous potassium carbonate (0.52 g, 3.77 mmol) and 2-chloro-1-(2-(trifluoromethyl)-10*H*-phenothiazin-10-yl)ethanone (**16**) (0.5 g, 1.45 mmol) in dry DMF (4 mL) under a set of conditions described in **Method C**, afforded white solid compound (**25**) (0.47 g, 63%); mp: 148–151 °C; IR (KBr) cm^−1^: 3422, 3282, 1694, 1627, 1569, 1328, 1117; ^1^H NMR: 8.62 (s, 1 H), 7.93 (s, 1 H), 7.80 (bs, 2 H), 7.59–7.55 (d, 2 H, *J* = 7.96), 7.51–7.47 (d, 2 H, *J* = 7.96), 7.42–7.38 (m, 1 H), 7.33–7.29 (m, 1 H), 4.35–4.29 (q, 2 H), 4.08 (s, 2 H), 1.38–1.34 (t, 3 H); MS (ESI) m/z: 506.2 (M^+^); Anal. Calcd for C_22_H_17_F_3_N_4_O_3_S_2_: C, 52.17; H, 3.38; N, 11.06; Found: C, 52.36; H, 3.26; N, 11.14%.

### Ethyl 4-amino-2-(2-(2-chloro-10*H*-phenothiazin-10-yl)-2-oxoethylthio)pyrimidine-5-carboxylate (26)

Reaction of ethyl 4-amino-2-mercaptopyrimidine-5-carboxylate (**10**) (0.38 g, 1.91 mmol), anhydrous potassium carbonate (0.55 g, 3.99 mmol) and 2-chloro-1-(2-chloro-10*H*-phenothiazin-10-yl)ethanone (**17**) (0.5 g, 1.61 mmol) in dry DMF (4 mL) under a set of conditions described in **Method C**, afforded white solid compound (**26**) (0.53 g, 70%); mp: 156–158 °C; IR (KBr) cm^−1^: 3471, 3358, 1676, 1608, 1580, 1520, 1459, 1374, 1336, 1236, 1163, 807; ^1^H NMR: 8.62 (s, 1 H), 7.79 (bs, 2 H), 7.67–7.66 (d, 1 H), 7.57–7.55 (dd, 1 H, *J* = 7.8, 0.8 Hz), 7.48–7.46 (dd, 1 H, *J* = 7.8, 1.36 Hz), 7.39–7.36 (m, 1 H), 7.35 (s, 1 H), 7.30–7.26 (m, 1 H), 7.24–7.21 (d, 1 H, *J* = 8.4 Hz), 4.34–4.29 (q, 2 H), 4.07 (s, 2 H), 1.38 (t, 3 H); ^13^C NMR: 172.97, 166.63, 165.33, 161.15, 158.36, 158.31, 139.22, 137.61, 131.88, 131.56, 131.23, 129.07, 128.10, 127.64, 127.45, 127.24, 127.07, 100.89, 60.63, 34.04, 14.04; MS (ESI) m/z: 494.1 (M + Na)^+^, 495.8 (M + 2 + Na)^+^; Anal. Calcd for C_21_H_17_ClN_4_O_3_S_2_: C, 53.33; H, 3.62; N, 11.85; Found: C, 53.66; H, 3.84; N, 11.96%.

### Ethyl 4-amino-2-(2-(2-methoxy-10*H*-phenothiazin-10-yl)-2-oxoethylthio)pyrimidine-5-carboxylate (27)

Reaction of ethyl 4-amino-2-mercaptopyrimidine-5-carboxylate (**10**) (0.38 g, 1.91 mmol), anhydrous potassium carbonate (0.55 g, 3.99 mmol) and 2-chloro-1-(2-methoxy-10*H*-phenothiazin-10-yl)ethanone (**18**) (0.5 g, 1.63 mmol) in dry DMF (4 mL) under a set of conditions described in **Method C**, afforded white solid compound (**27**) (0.44 g, 57%); mp: 104–106 °C; IR (KBr) cm^−1^: 3442, 3333, 1692, 1604, 1571, 1464, 1371, 1194, 1024; ^1^H NMR: 8.60 (s, 1 H), 7.77 (bs, 2 H), 7.58–7.56 (d, 1 H), 7.47–7.45 (d, 1 H, *J* = 7.6 Hz), 7.35 (s, 1 H), 7.33–7.32 (m, 1 H), 7.26–7.24 (d, 1 H, *J* = 7.6 Hz), 7.24–7.21 (m, 1 H), 6.84–6.81 (d, 1 H), 4.34–4.28 (q, 2 H), 4.06 (s, 2 H), 3.81 (s, 3 H), 1.37–1.33 (t, 3 H); ^13^C NMR: 173.97, 166.87, 166.22, 161.92, 159.30, 159.04, 139.75, 138.49, 128.51, 128.16, 127.18, 127.10, 113.59, 113.07, 101.34, 60.96, 55.73, 35.50, 14.25; MS (ESI) m/z: 468.2 (M^+^); Anal. Calcd for C_22_H_20_N_4_O_4_S_2_: C, 56.39; H, 4.30; N, 11.96; Found: C, 56.22; H, 4.44; N, 11.82%.

### Ethyl 4-amino-2-(2-oxo-2-(10*H*-phenothiazin-10-yl)ethylthio)pyrimidine-5-carboxylate (28)

Reaction of ethyl 4-amino-2-mercaptopyrimidine-5-carboxylate (**10**) (0.43 g, 2.16 mmol), anhydrous potassium carbonate (0.62 g, 4.49 mmol) and 2-chloro-1-(10*H*-phenothiazin-10-yl)ethanone (**19**) (0.5 g, 1.81 mmol) in dry DMF (4 mL) under a set of conditions described in **Method C**, afforded white solid compound (**28**) (0.53 g, 67%); mp: 202–204 °C; IR (KBr) cm^−1^: 3399, 3278, 1704, 1669, 1632, 1573, 1461, 1335, 761; ^1^H NMR: 8.51 (s, 1 H), 7.69–7.67 (d, 2 H, *J* = 7.7 Hz), 7.53 (bs, 2 H), 7.53–7.51 (d, 2 H, *J* = 7.7 Hz), 7.41–7.37 (m, 2 H), 7.32–7.28 (m, 2 H), 4.32–4.27 (q, 2 H), 4.17 (s, 2 H), 1.36–1.32 (t, 3 H); MS (ESI) m/z: 438.4 (M^+^). Anal. Calcd for C_21_H_17_N_3_O_3_S_2_: C, 59.56; H, 4.05; N, 9.92; Found: C, 59.72; H, 4.16; N, 9.84%.

### 2-(5-(4-Pyridyl)-1,3,4-oxadiazol-2-ylthio)-1-(2-trifluoromethyl-10*H*-phenothiazin-10-yl)ethanone (29)

Reaction of 5-(pyridin-4-yl)-1,3,4-oxadiazole-2-thiol (**20**) (0.32 g, 1.79 mmol), anhydrous potassium carbonate (0.52 g, 3.77 mmol) and 2-chloro-1-(2-trifluoromethyl-10*H*-phenothiazin-10-yl)ethanone (**16**) (0.5 g, 1.45 mmol) in dry DMF (4 mL) under a set of conditions described in **Method C**, afforded white solid compound (**29**) (0.45 g, 64%); mp: 156–158 °C; IR (KBr) cm^−1^: 1680, 1464, 1330, 1169; ^1^H NMR: 8.80–8.79 (d, 2 H, *J* = 6.1 Hz), 7.93 (s, 1 H), 7.83–7.82 (d, 2 H, *J* = 6.1 Hz), 7.65–7.63 (d, 1 H), 7.59–7.57 (d, 1 H), 7.52–7.49 (d, 2 H), 7.46–7.41 (m, 1 H), 7.37–7.33 (m, 1 H), 4.22 (s, 2 H); ^13^C NMR: 165.40, 164.92, 164.20, 150.90, 138.09, 137.08, 130.47, 129.84, 129.51, 128.63, 128.35, 128.20, 126.67, 124.92, 124.38, 124.1, 122.21, 120.0, 36.96 MS (EI) m/z: 486.6 (M^+^); Anal. Calcd for C_22_H_13_F_3_N_4_O_2_S_2_: C, 54.31; H, 2.69; N, 11.52; Found: C, 54.44; H, 2.82; N, 11.40%.

### 1-(2-Chloro-10*H*-phenothiazin-10-yl)-2-(5-(4-pyridyl)-1,3,4-oxadiazol-2-ylthio)ethanone (30)

Reaction of 5-(pyridin-4-yl)-1,3,4-oxadiazole-2-thiol (**20**) (0.34 g, 1.89 mmol), anhydrous potassium carbonate (0.55 g, 3.99 mmol) and 2-chloro-1-(2-chloro-10*H*-phenothiazin-10-yl)ethanone (**17**) (0.5 g, 1.61 mmol) in dry DMF (4 mL) under a set of conditions described in **Method C**, afforded white solid compound (**30**) (0.54 g, 74%); mp: 194–196 °C; IR (KBr) cm^−1^: 1679, 1576, 1461, 1334, 1235, 1166, 700; ^1^H NMR: 8.80–8.78 (d, 2 H, *J = *6.1 Hz), 7.83–7.82 (d, 2 H, *J* = 6.1 Hz), 7.67 (s, 1 H), 7.62–7.60 (d, 1 H), 7.50–7.48 (d, 1 H), 7.42–7.40 (m, 1 H), 7.39–7.37 (d, 1 H), 7.33–7.29 (m, 1 H), 7.27–7.24 (d, 1 H), 4.30 (s, 2 H); MS (ESI) m/z: 452.2 (M)^+^, 454.2 (M + 2)^+^; Anal. Calcd for C_21_H_13_ClN_4_O_2_S_2_: C, 55.69; H, 2.89; N, 12.37; Found: C, 55.84; H, 2.76; N, 12.48%.

### 1-(2-Methoxy-10*H*-phenothiazin-10-yl)-2-(5-(4-pyridyl)-1,3,4-oxadiazol-2-ylthio)-ethanone (31)

Reaction of 5-(pyridin-4-yl)-1,3,4-oxadiazole-2-thiol (**20**) (0.34 g, 1.89 mmol), anhydrous potassium carbonate (0.55 g, 3.99 mmol) and 2-chloro-1-(2-methoxy-10*H*-phenothiazin-10-yl)ethanone (**18**) (0.5 g, 1.63 mmol) in dry DMF (4 mL) under a set of conditions described in **Method C**, afforded white solid compound (**31**) (0.48 g, 66%); mp: 166–168 °C; IR (KBr) cm^−1^: 1671, 1596, 1450, 1345, 1021, 746; ^1^H NMR: 8.80–8.78 (d, 2 H, *J* = 6.0 Hz), 7.83–7.82 (d, 2 H, *J = *6.0 Hz), 7.62–7.60 (d, 1 H, *J* = 7.8 Hz), 7.48–7.46 (d, 1 H, *J* = 7.8 Hz), 7.38–7.35 (m, 1 H), 7.34 (s, 1 H), 7.29–7.27 (m, 1 H), 7.25–7.23 (d, 1 H), 6.86–6.84 (d, 1 H), 4.45 (s, 2 H), 3.83 (s, 3 H); MS (ESI) m/z: 448.6 (M^+^); Anal. Calcd for C_22_H_16_N_4_O_3_S_2_: C, 58.91; H, 3.60; N, 12.49; Found: C, 58.78; H, 3.46; N, 12.62%.

### 1-(10*H*-Phenothiazin-10-yl)-2-(5-(4-pyridyl)-1,3,4-oxadiazol-2-ylthio)ethanone (32)

Reaction of 5-(pyridin-4-yl)-1,3,4-oxadiazole-2-thiol (**20**) (0.39 g, 2.18 mmol), anhydrous potassium carbonate (0.62 g, 4.49 mmol) and 2-chloro-1-(10*H*-phenothiazin-10-yl)ethanone (**19**) (0.5 g, 1.81 mmol) in dry DMF (4 mL) under a set of conditions described in **Method C**, afforded white solid compound (**32**) (0.54 g, 71%); mp: 212–214 °C; IR (KBr) cm^−1^: 1667, 1462, 1350, 757; ^1^H NMR: 8.79–8.77 (d, 2 H, *J* = 6.0 Hz), 7.82–7.81 (d, 2 H, *J* = 6.0 Hz), 7.63–7.61 (d, 2 H), 7.49–7.47 (d, 2 H), 7.39–7.35 (m, 2 H), 7.30–7.26 (m, 2 H), 4.44 (s, 2 H); MS (ESI) m/z: 418.6 (M^+^); Anal. Calcd for C_21_H_14_N_4_O_2_S_2_: C, 60.27; H, 3.37; N, 13.39; Found: C, 60.39; H, 3.26; N, 13.54%.

### 2-(4-Amino-5-(4-pyridyl)-4*H*-1,2,4-triazol-3-ylthio)-1-(2-(trifluoromethyl)-10*H*-phenothiazin-10-yl)ethanone (33)

Reaction of 4-amino-5-(4-pyridyl)-4*H*-1,2,4-triazole-3-thiol (**21**) (0.35 g, 1.81 mmol), anhydrous potassium carbonate (0.52 g, 3.77 mmol) and 2-chloro-1-(2-trifluoromethyl-10*H*-phenothiazin-10-yl)ethanone (**16**) (0.5 g, 1.45 mmol) in dry DMF (4 mL) under a set of conditions described in **Method C**, afforded white solid compound (**33**) (0.36 g, 49%); mp: 202–204 °C; IR (KBr) cm^−1^: 3321, 3111, 2965, 2924, 1670, 1606, 1464, 1331, ^1^H NMR: 8.70–8.68 (d, 2 H, *J* = 5.6 Hz), 8.03–8.01 (d, 2 H, *J* = 5.6 Hz), 8.01 (s, 1 H), 7.82–7.80 (d, 1 H), 7.75–7.73 (d, 1 H, *J* = 8.3 Hz), 7.62–7.60 (d, 1 H, *J* = 8.3 Hz), 7.58–7.56 (d, 1 H), 7.47–7.44 (m, 1 H), 7.39–7.35 (m, 1 H), 6.22 (s, 2 H), 4.54 (bs, 2 H); MS (EI) m/z: 500.89 (M^+^); Anal. Calcd for C_22_H_15_F_3_N_6_OS_2_: C, 52.79; H, 3.02; N, 16.79; Found: 52.96; H, 3.16; N, 16.66%.

### 2-(4-Amino-5-(4-pyridyl)-4*H*-1,2,4-triazol-3-ylthio)-1-(2-chloro-10*H*-phenothiazin-10-yl)ethanone (34)

Reaction of 4-amino-5-(4-pyridyl)-4*H*-1,2,4-triazole-3-thiol (**21**) (0.37 g, 1.91 mmol), anhydrous potassium carbonate (0.55 g, 3.99 mmol) and 2-chloro-1-(2-chloro-10*H*-phenothiazin-10-yl)ethanone (**17**) (0.5 g, 1.61 mmol) in dry DMF under a set of conditions described in **Method C**, afforded white solid compound (**34**) (0.45 g, 60%); mp: 225–227 °C; IR (KBr) cm^−1^: 3320, 3106, 1666, 1602, 1459, 1402, 1335, 1230, 1167, 760; ^1^H NMR: 8.69–8.68 (d, 2 H, *J* = 4.8 Hz), 8.07–8.06 (d, 2 H, *J* = 4.8 Hz), 7.71 (s, 1 H), 7.71–7.69 (d, 1 H), 7.52–7.50 (d, 1 H), 7.47–7.45 (d, 1 H), 7.42–7.38 (m, 1 H), 7.34–7.32 (m, 1 H), 7.30–7.28 (d, 1 H), 6.12 (s, 2 H), 4.45 (bs, 2 H); MS (ESI) m/z: 465.9 (M)^+^ and 467.6 (M + 2)^+^; Anal. Calcd for C_21_H_15_ClN_6_OS_2_: C, 54.01; H, 3.24; N, 18.00; Found: C, 54.16; H, 3.10; N, 18.12%.

### 2-(4-Amino-5-(4-pyridyl)-4*H*-1,2,4-triazol-3-ylthio)-1-(2-methoxy-10*H*-phenothiazin-10-yl)ethanone (35)

Reaction of 4-amino-5-(4-pyridyl)-4*H*-1,2,4-triazole-3-thiol (**21**) (0.37 g, 1.91 mmol), anhydrous potassium carbonate (0.55 g, 3.99 mmol) and 2-chloro-1-(2-methoxy-10*H*-phenothiazin-10-yl)ethanone (**18**) (0.5 g, 1.63 mmol) in dry DMF (4 mL) under a set of conditions described in **Method C**, afforded white solid compound (**35**) (0.46 g, 61%); mp: 188–190 °C; IR (KBr) cm^−1^: 3318, 3207, 1654, 1601, 1462, 1360, 1213; ^1^H NMR: 8.70–8.68 (d, 2 H, *J* = 6.1 Hz), 8.03–8.01 (d, 2 H, *J* = 6.1 Hz), 7.68–7.65 (d, 1 H), 7.52–7.50 (d, 1 H), 7.42–7.40 (d, 1 H, *J* = 8.7 Hz), 7.39 (s, 1 H), 7.38–7.36 (m, 1 H), 7.31–7.30 (m, 1 H), 6.93–6.90 (d, 1 H, *J* = 8.7 Hz), 6.22 (s, 2 H), 4.40 (bs, 2 H), 3.81 (s, 3 H); MS (EI) m/z: 462.1 (M^+^); Anal. Calcd for C_22_H_18_N_6_O_2_S_2_: C, 57.13; H, 3.92; N, 18.17; Found: C, 57.36; H, 3.76; N, 18.30%.

### 2-(4-Amino-5-(4-pyridyl)-4*H*-1,2,4-triazol-3-ylthio)-1-(10*H*-phenothiazin-10-yl)ethanone (36)

Reaction of 4-amino-5-(4-pyridyl)-4*H*-1,2,4-triazole-3-thiol (**21**) (0.42 g, 2.17 mmol), anhydrous potassium carbonate (0.62 g, 4.49 mmol) and 2-chloro-1-(10*H*-phenothiazin-10-yl)ethanone (**19**) (0.5 g, 1.81 mmol) in dry DMF (4 mL) under a set of conditions described in **Method C**, afforded white solid compound (**36**) (0.46 g, 59%); mp: 232–234 °C; IR (KBr) cm^−1^: 3308, 3202, 1652, 1605, 1458, 1368; ^1^H NMR: 8.69–8.68 (d, 2 H, *J* = 6.1 Hz), 8.03–8.02 (d, 2 H, *J* = 6.1 Hz), 7.73–7.71 (d, 2 H), 7.54–7.52 (d, 2 H), 7.42–7.38 (m, 2 H), 7.33–7.29 (m, 2 H), 6.20 (s, 2 H), 4.37 (bs, 2 H); MS (EI) m/z: 432.80 (M^+^); Anal. Calcd for C_21_H_16_N_6_OS_2_: C, 58.31; H, 3.73; N, 19.43; Found: C, 58.48; H, 3.84; N, 19.30%.

### 10-(3-Chloropropyl)-2-trifluoromethyl-10*H*-phenothiazine (38)

**Method D:** Commercially available 2-trifluoromethyl-10*H*-phenothiazine (**11**) (1 g, 3.75 mmol) was dissolved in dry THF (5 mL) and a suspension of NaH (0.13 g, 5.42 mmol) in dry DMSO (10 mL) and THF (5 mL) was added to it. The reaction mixture was stirred in the presence of nitrogen at 0 °C for 30 min. A solution of 1-bromo-3-chloropropane (**37**) (1.74 g, 11.08 mmol) in dry DMSO (5 mL) was added to the above suspension. The reaction mixture was then stirred at room temp under nitrogen for 4 h and completion of the reaction was checked by TLC. On completion of the reaction, the reaction mixture was poured into 50 mL of ice water and extracted with DCM (3 × 75 mL) yielding the desired white solid compounds (**38**)^72^ (0.87 g, 68%); mp: 72–74 °C (Lit^73^: 72 °C); IR (KBr) cm^−1^: 2877, 1600, 1425, 1242, 1108, 750; MS (ESI) m/z: 342.9 (M + 1)^+^.

### 2-Chloro-10-(3-chloropropyl)-10*H*-phenothiazine (39)

Reaction of 2-chloro-10*H*-phenothiazine (**12**) (1 g, 4.28 mmol), NaH (0.15 g, 6.25 mmol) and 1-bromo-3-chloropropane (**37**) (2.03 g, 12.93 mmol) in dry THF and DMSO under a set of conditions described in **Method D**, afforded yellow colored liquid compound (**39**) (0.99 g, 75%); IR (KBr) cm^−1^: 2929, 1637, 1457, 1026, 751; MS (ESI) m/z: 301 (M + 1)^+^ and 303 (M + 3)^+^.

### 10-(3-Chloropropyl)-2-methoxy-10*H*-phenothiazine (40)

Reaction of 2-methoxy-10*H*-phenothiazine (**13**) (1 g, 4.08 mmol), NaH (0.15 g, 6.25 mmol) and 1-bromo-3-chloropropane (**37**) (1.93 g, 12.29 mmol) in dry THF and DMSO under a set of conditions described in **Method D**, afforded yellow colored liquid compound (**40**) (0.86 g, 65%); IR (KBr) cm^−1^: 2938, 1580, 1457, 1267, 1121, 1028, 759; MS (ESI) m/z: 305.1 (M + 1)^+^.

### 10-(3-Chloropropyl)-10*H*-phenothiazine (41)

Reaction of 10*H*-phenothiazine (**14**) (1 g, 5.02 mmol), NaH (0.18 g, 7.50 mmol) and 1-bromo-3-chloropropane (**37**) (2.36 g, 15.03 mmol) in dry THF and DMSO under a set of conditions described in **Method D**, afforded white solid compound (**41**) (1.08 g, 78%); mp: 60–62 °C (Lit^74^: 60 °C); IR (KBr) cm^−1^: 2956, 1590, 1457, 1332, 1248, 1194, 1037, 756; MS (ESI) m/z: 275.2 (M + 1)^+^.

### 4-Amino-2-(3-(2-trifluoromethyl-10*H*-phenothiazin-10-yl)propylthio)pyrimidine-5-carbonitrile (42)

Reaction of 4-amino-2-mercaptopyrimidine-5-carbonitrile (**9**) (0.27 g, 1.77 mmol), anhydrous potassium carbonate (0.52 g, 3.77 mmol) and 10-(3-chloropropyl)-2-trifluoromethyl-10*H*-phenothiazine (**38**) (0.5 g, 1.45 mmol) in dry DMF (4 mL) under a set of conditions described in **Method C**, afforded white solid compound (**42**) (0.4 g, 60%); mp: 164–166 °C; IR (KBr) cm^−1^: 3475, 3306, 2220, 1645, 1573, 1538, 1470, 1423, 1363, 1329, 1247, 1107; ^1^H NMR: 8.23 (s, 1 H), 7.72 (bs, 2 H), 7.31–7.29 (d, 1 H), 7.21–7.19 (m, 2 H), 7.16–7.14 (d, 1 H), 7.13 (s, 1 H), 7.06–7.04 (d, 1 H, *J* = 7.92), 7.00–6.98 (m, 1 H), 4.09–4.05 (t, 2 H), 3.18–3.14 (t, 2 H), 2.14–2.10 (m, 2 H); MS (EI): 459.3 (M^+^); Anal. Calcd for C_21_H_16_F_3_N_5_S_2_: C, 54.89; H, 3.51; N, 15.24; Found: C, 54.72; H, 3.42; N, 15.38%.

### 4-Amino-2-(3-(2-chloro-10*H*-phenothiazin-10-yl)propylthio)pyrimidine-5-carbonitrile (43)

Reaction of 4-amino-2-mercaptopyrimidine-5-carbonitrile (**9**) (0.29 g, 1.91 mmol), anhydrous potassium carbonate (0.55 g, 3.99 mmol) and 2-chloro-10-(3-chloropropyl)-10*H*-phenothiazine (**39**) (0.5 g, 1.61 mmol) in dry DMF (4 mL) under a set of conditions described in **Method C**, afforded white solid compound (**43**) (0.33 g, 48%); mp: 138–140 °C; IR (KBr) cm^−1^: 3444, 3343, 2218, 1631, 1572, 1534, 1456, 1366, 1237, 777; ^1^H NMR: 8.23 (s, 1 H), 7.70 (bs, 2 H), 7.18–7.16 (m, 1 H), 7.13–7.11 (d, 1 H), 7.09–7.07 (d, 1 H, *J* = 8.1 Hz), 7.02–7.00 (d, 1 H), 6.97 (s, 1 H), 6.97–6.95 (m, 1 H), 6.94–6.91 (d, 1 H, *J* = 8.1 Hz), 4.02–3.98 (t, 2 H), 3.16–3.13 (t, 2 H), 2.14–2.11 (m, 2 H); MS (ESI) m/z: 446.5 (M + Na^+^), 448.5 (M + 2 + Na)^+^; Anal. Calcd for C_20_H_16_ClN_5_S_2_: C, 56.39; H, 3.79; N, 16.44; Found: C, 56.54; H, 3.66; N, 16.58%.

### 4-Amino-2-(3-(2-methoxy-10*H*-phenothiazin-10-yl)propylthio)pyrimidine-5-carbonitrile (44)

Reaction of 4-amino-2-mercaptopyrimidine-5-carbonitrile (**9**) (0.29 g, 1.91 mmol), anhydrous potassium carbonate (0.55 g, 3.99 mmol) and 10-(3-chloropropyl)-2-methoxy-10*H*-phenothiazine (**40**) (0.5 g, 1.63 mmol) in dry DMF (4 mL) under a set of conditions described in **Method C**, afforded white solid compound (**44**) (0.28 g, 40%); mp: 158–160 °C; IR (KBr) cm^−1^: 3414, 3340, 2217, 1655, 1569, 1463, 1373, 1156, 1036; ^1^H NMR: 8.12 (s, 1 H), 7.09–7.04 (m, 2 H), 6.98–6.96 (d, 1 H, *J* = 8.0 Hz), 6.87–6.82 (m, 1 H), 6.81–6.79 (d, 1 H, *J* = 8.0 Hz), 6.44–6.41 (d, 1 H), 6.41 (s, 1 H), 5.44 (bs, 2 H), 3.92–3.89 (t, 2 H), 3.70 (s, 3 H), 3.12–3.09 (t, 2 H), 2.17–2.12 (m, 2 H); ^13^C NMR: 174.06, 161.30, 159.49, 159.22, 145.84, 144.24, 127.05, 126.79, 126.70, 124.76, 122.07, 115.33, 115.16, 114.94, 106.64, 102.86, 85.41, 54.91, 45.12, 27.29, 25.91; MS (ESI) m/z: 420.7 (M)^+^; Anal. Calcd for C_21_H_19_N_5_OS_2_: C, 59.83; H, 4.54; N, 16.61; Found: C, 59.97; H, 4.40; N, 16.76%.

### 4-Amino-2-(3-(10*H*-phenothiazin-10-yl)propylthio)pyrimidine-5-carbonitrile (45)

Reaction of 4-amino-2-mercaptopyrimidine-5-carbonitrile (**9**) (0.33 g, 2.17 mmol), anhydrous potassium carbonate (0.62 g, 4.49 mmol) and 10-(3-chloropropyl)−10*H*-phenothiazine (**41**) (0.5 g, 1.81 mmol) in dry DMF (4 mL) under a set of conditions described in **Method C**, afforded white solid compound (**45**) (0.43 g, 61%); mp: 117–119 °C; IR (KBr) cm^−1^: 3353, 3160, 2224, 1661, 1573, 1542, 1459, 1360, 1239, 781; ^1^H NMR: 8.19 (s, 1 H), 7.18–7.16 (m, 2 H), 7.15–7.13 (d, 2 H), 6.96–6.92 (m, 2 H), 6.90–6.88 (d, 2 H), 5.33 (bs, 2 H) 4.05–4.02 (t, 2 H), 3.22–3.18 (t, 2 H), 2.25–2.18 (m, 2 H); ^13^C NMR: 175.88, 161.46, 159.49, 145.28, 127.64, 127.33, 125.61, 122.69, 115.70, 115.06, 86.13, 45.63, 28.42, 26.56; MS (EI) m/z: 391.5 (M)^**+**^; Anal. Calcd for C_20_H_17_N_5_S_2_: C, 61.36; H, 4.38; N, 17.89; Found: C, 61.54; H, 4.23; N, 17.76%.

### Ethyl 4-amino-2-(3-(2-trifluoromethyl-10*H*-phenothiazin-10-yl)propylthio)pyrimidine-5-carboxylate (46)

Reaction of ethyl 4-amino-2-mercaptopyrimidine-5-carboxylate (**10**) (0.36 g, 1.81 mmol), anhydrous potassium carbonate (0.52 g, 3.77 mmol) and 10-(3-chloropropyl)-2-(trifluoromethyl)-10*H*-phenothiazine (**38**) (0.5 g, 1.45 mmol) in dry DMF (4 mL) under a set of conditions described in **Method C**, afforded white solid compound (**46**) (0.49 g, 67%); mp: 118–120 °C; IR (KBr) cm^−1^: 3422, 3282, 2912, 1697, 1472, 1421, 1113; ^1^H NMR: 8.60 (s, 1 H), 7.76 (bs, 2 H), 7.23–7.21 (d, 1 H, *J* = 8.0 Hz), 7.17–7.13 (m, 3 H), 7.04 (s, 1 H), 6.98–6.94 (m, 1 H), 6.92–6.90 (d, 1 H, *J* = 8.0 Hz), 4.36–4.30 (q, 2 H), 4.06–4.03 (t, 2 H), 3.23–3.20 (t, 2 H), 2.27–2.20 (m, 2 H), 1.39–1.36 (t, 3 H); MS (EI) m/z: 506.68 (M^+^); Anal. Calcd for C_23_H_21_F_3_N_4_O_2_S_2_: C, 54.53; H, 4.18; N, 11.06; Found: C, 54.66; H, 4.02; N, 11.19%.

### Ethyl 4-amino-2-(3-(2-chloro-10*H*-phenothiazin-10-yl)propylthio)pyrimidine-5-carboxylate (47)

Reaction of ethyl 4-amino-2-mercaptopyrimidine-5-carboxylate (**10**) (0.38 g, 1.91 mmol), anhydrous potassium carbonate (0.55 g, 3.99 mmol) and 2-chloro-10-(3-chloropropyl)-10*H*-phenothiazine (**39**) (0.5 g, 1.61 mmol) in dry DMF (4 mL) under a set of conditions described in **Method C**, afforded white solid compound (**47**) (0.37 g, 49%); mp: 120–122 °C; IR (KBr) cm^−1^: 3412, 3268, 1695, 1458, 1381, 1089, 749; ^1^H NMR: 8.40 (s, 1 H), 7.84 (bs, 2 H), 6.96–6.92 (d, 1 H, *J* = 8.2 Hz), 6.91 (s, 1 H),6.83–6.81 (d, 1 H, *J* = 8.2 Hz), 6.74–6.70 (m, 1 H), 6.68–6.63 (m, 3 H), 4.14–4.09 (q, 2 H), 3.79–3.75 (t, 2 H), 3.00–2.95 (t, 2 H), 2.05–1.98 (m, 2 H), 1.18–1.14 (t, 3 H); MS (ESI) m/z: 472.5 (M)^+^, 474.5 (M + 2)^+^; Anal. Calcd for C_22_H_21_ClN_4_O_2_S_2_: C, 55.86; H, 4.47; N, 11.84; Found: C, 55.71; H, 4.60; N, 11.98%.

### Ethyl 4-amino-2-(3-(2-methoxy-10*H*-phenothiazin-10-yl)propylthio)pyrimidine-5-carboxylate (48)

Reaction of ethyl 4-amino-2-mercaptopyrimidine-5-carboxylate (**10**) (0.38 g, 1.91 mmol), anhydrous potassium carbonate (0.55 g, 3.99 mmol) and 10-(3-chloropropyl)-2-methoxy-10*H*-phenothiazine (**40**) (0.5 g, 1.63 mmol) in dry DMF (4 mL) under a set of conditions described in **Method C**, afforded white solid compound (**48**) (0.41 g, 53%); mp: 106–108 °C; IR (KBr) cm^−1^: 3410, 3268, 1691, 1430, 1382, 1102; ^1^H NMR: 8.61 (s, 1 H), 7.74 (bs, 2 H), 7.15–7.11 (m, 2 H), 7.04–7.02 (d, 1 H, *J = *8.1 Hz), 6.92–6.90 (m, 1 H), 6.89–6.87 (d, 1 H, *J* = 8.1 Hz), 6.49 (s, 1 H), 6.49–6.47 (d, 1 H), 4.35–4.30 (q, 2 H), 4.00–3.96 (t, 2 H), 3.76 (s, 3 H), 3.21–3.17 (t, 2 H), 2.28–2.22 (m, 2 H), 1.38–1.35 (t, 3 H); ^13^C NMR: 175.34, 166.35, 161.80, 159.72, 158.97, 146.55, 144.97, 127.69, 127.46, 127.15, 125.70, 122.56, 116.20, 115.57, 106.77, 103.49, 101.15, 60.95, 55.50, 46.02, 28.25, 26.75, 14.29; MS (ESI) m/z: 468.5 (M)^+^; Anal. Calcd for C_23_H_24_N_4_O_3_S_2_: C, 58.95; H, 5.16; N, 11.96; Found: C, 58.84; H, 5.29; N, 11.81%.

### Ethyl 4-amino-2-(3-(10*H*-phenothiazin-10-yl)propylthio)pyrimidine-5-carboxylate (49)

Reaction of ethyl 4-amino-2-mercaptopyrimidine-5-carboxylate (**10**) (0.43 g, 2.16 mmol), anhydrous potassium carbonate (0.62 g, 4.49 mmol) and 10-(3-chloropropyl)-10*H*-phenothiazine (**41**) (0.5 g, 1.81 mmol) in dry DMF (4 mL) under a set of conditions described in **Method C**, afforded white solid compound (**49**) (0.33 g, 42%); mp: 146–148 °C; IR (KBr) cm^−1^: 3416, 3270, 1698, 1533, 1455, 1368, 1053; ^1^H NMR: 8.60 (s, 1 H), 7.72 (bs, 2 H), 7.16–7.12 (m, 4 H), 6.93–6.88 (m, 4 H), 4.35–4.29 (q, 2 H), 4.04–4.00 (t, 2 H), 3.22–3.19 (t, 2 H), 2.26–2.20 (m, 2 H), 1.38–1.35 (t, 3 H); MS (ESI) m/z: 438.7 (M)^+^; Anal. Calcd for C_22_H_22_N_4_O_2_S_2_: C, 60.25; H, 5.06; N, 12.78; Found: C, 60.44; H, 5.22; N, 12.63%.

### Biology

#### *In vitro* blood-brain permeability assay

To assess the *in vivo* brain permeability potential of the test compounds, PAMPA assay for BBB was performed as reported by Di. *et al*.^57^. The donor microplate (polyvinylidene fluoride (PVDF) membrane, diameter 25 mm, pore size 0.45 µm Millipore) and the acceptor microplate were used for the experiment. The acceptor microplate was filled with 200 µl phosphate buffer saline (PBS) and ethanol in the ratio of 70:30 whereas the donor microplate filter surface was impregnated with porcine brain lipid (Avanti Polar Lipids) in dodecane (Sigma) (4 µl of 20 mg/ml). 200 µl of the test compound (100 µg/ml in PBS/ethanol) was added to the donor well. A sandwich was formed by placing the donor plate carefully on the acceptor plate, and the plates were kept undisturbed for 120 min at 25 °C. The donor plate was carefully detached after the incubation period and concentration of the test compound in the acceptor wells was determined using UV spectroscopy. Each sample was analyzed in four wells at five different wavelengths, and at least in three independent runs. Assay validation was performed by using nine commercial quality standard drugs of known BBB permeability^75^ in which the reported permeability values of the standards were compared with their experimental permeability values, which gave a linear relationship, *P*_*e*_(exp.) = 1.171*P*_*e*_(bibl.) + 1.489 (R^2^ = 0.983)^57,58^. From this equation and considering the limit established by Di. *et al*.^57^, the range of permeability (*P*_e_) was determined which suggested that compounds having *P*_e_ value less than 3.8 × 10^−6^ cm s^−1^ resulted in low BBB permeation.

#### Acute hypophagic response in animal model

Male Wistar rats weighing 200–250 g were used in the present study to assess the acute hypophagic response. Animals were housed individually in a plexi-glass cage in a temperature and humidity controlled room with a 12 h light/dark cycle. Standard chow pellets and water were made available *ad libitum*. Animals were handled daily for a week before performing the actual experiments. The animal study protocol was approved by the Institutional Animal Ethics Committee (IAEC) of Faculty of Pharmacy, The Maharaja Sayajirao University of Baroda (Approval no. MSU/IAEC/2014-15/1401). The experiments were conducted in accordance to the guidelines of CPCSEA (Committee for the Purpose of Control and Supervision of Experiments on Animals), Ministry of Environment & Forests, Govt. of India. We tried our best to reduce the number of animals and their suffering.

The acute effect of the synthesized test compounds on feeding behavior was assessed in 24 h food-deprived animals. Before performing the experiments, the bedding material was removed from the cage. The animals were deprived of feed for 24 h with free access to water. The fasted animals were administered with the test compounds (10 mg/kg, p.o.). After 30 min of administration, weighed amounts of food pellets were served to the animals. Amount of food pellets and food spillage remaining after 2 h were weighed to find out the amount of food consumed by the animals.

In another set of experiments, *in vivo* CB1 receptor antagonistic activity of the test compounds was assessed. WIN-55212-2, a selective CB1 receptor agonist is known to increase the food intake. To assess the CB1 receptor antagonistic activity of the test compounds, 24 h fasted animals were given WIN-55212-2 (2 mg/kg, i.p.), 15 min prior to the administration of the test compounds and the experiment was proceeded as described above.

## Electronic supplementary material

Supplementary Information
