# Supplementary material for: New role of phenothiazine derivatives as peripherally acting CB1 receptor antagonizing anti-obesity agents
Source: Sci Rep. 2018 Jan 26;8:1650. doi: 10.1038/s41598-018-20078-w (PMC5785958; doi:10.1038/s41598-018-20078-w)
Supplement: Supplementary file 1 — Supplementary Information [file 41598_2018_20078_MOESM1_ESM.pdf]

Supporting Information

New role of phenothiazine derivatives as peripherally acting CB1 receptor antagonizing anti-obesity agents

Mayank Kumar Sharma, Jatin Machhi, Prashant Murumkar, Mange Ram Yadav*

Faculty of Pharmacy, Kalabhavan Campus,
The Maharaja Sayajirao University of Baroda, Vadodara-390 001, Gujarat, India.

Table of Content

^{1}H NMR Spectra……S2

^{13}C NMR Spectra……S14

^{1}H-NMR Spectra.

4-Amino-2-(2-(2-trifluoromethyl-10*H*-phenothiazin-10-yl)-2-oxoethylthio)pyrimidine-5-carbonitrile (5)

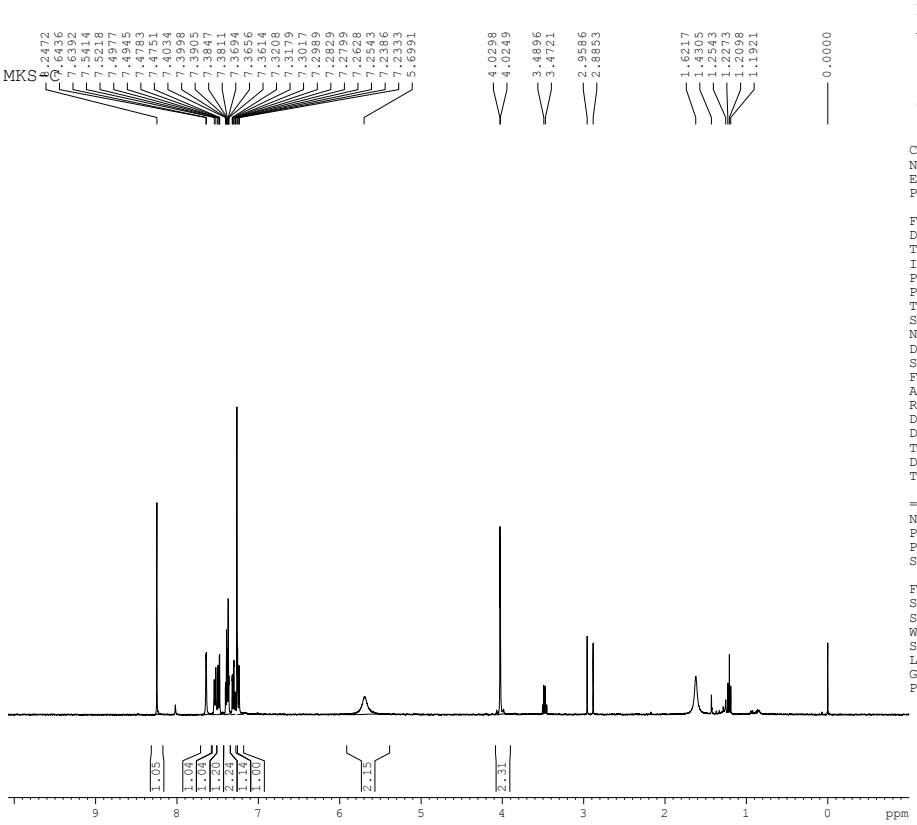

BRUKER
AVANCE II 400 NMR
Spectrometer
SAIF
Panjab University
Chandigarh

```
Current Data Parameters
NAME          Feb17-2016
EXPNO                240
PROCNO                 1

F2 - Acquisition Parameters
Date_          20160217
Time              18.13
INSTRUM           spect
PROBHD   5 mm PABBO BB-
PULPROG            zg30
TD                65536
SOLVENT           CDCl3
NS                    8
DS                    2
SWH           12019.230 Hz
FIDRES         0.183399 Hz
AQ            2.7263477 sec
RG                  912
DW               41.600 usec
DE                6.00 usec
TE               295.2 K
D1           1.00000000 sec
TD0                   1

======== CHANNEL f1 ========
NUC1                  1H
P1                10.90 usec
PL1               -3.00 dB
SFO1        400.1324710 MHz

F2 - Processing parameters
SI                32768
SF          400.1300086 MHz
WDW                  EM
SSB                   0
LB                0.30 Hz
GB                    0
PC                1.00
```

manishkumarmanu1986@gmail.com

4-Amino-2-(2-(2-chloro-10*H*-phenothiazin-10-yl)-2-oxoethylthio)pyrimidine-5-carbonitrile (22)

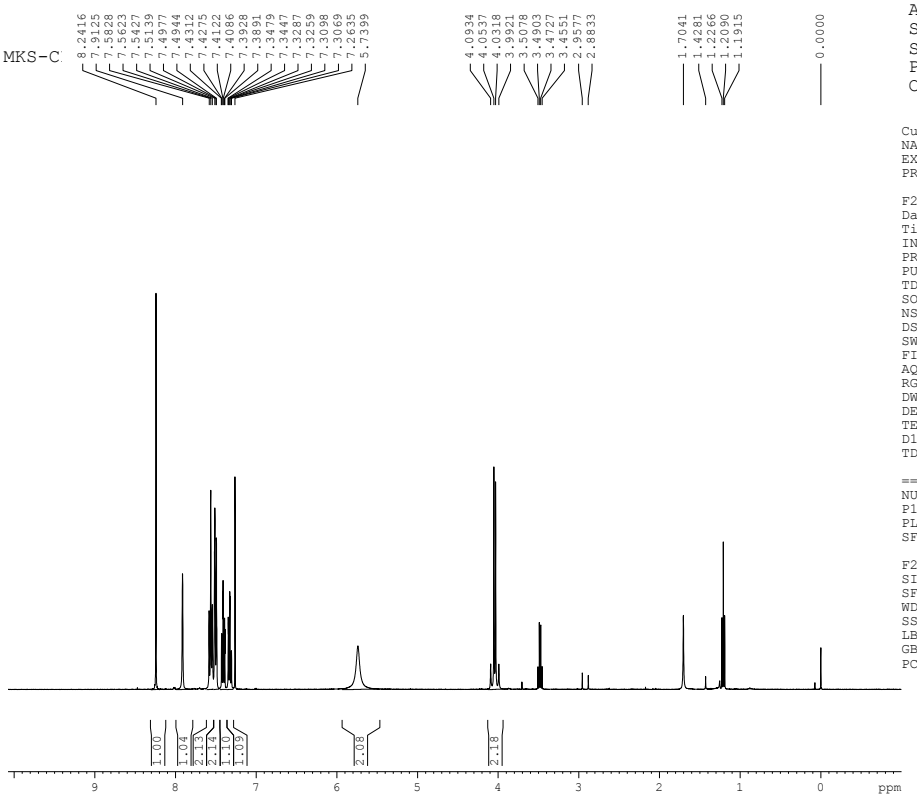

BRUKER
AVANCE II 400 NMR
Spectrometer
SAIF
Panjab University
Chandigarh

```
Current Data Parameters
NAME          Feb17-2016
EXPNO                250
PROCNO                 1

F2 - Acquisition Parameters
Date_          20160217
Time              18.18
INSTRUM           spect
PROBHD   5 mm PABBO BB-
PULPROG            zg30
TD                65536
SOLVENT           CDCl3
NS                    8
DS                    2
SWH           12019.230 Hz
FIDRES         0.183399 Hz
AQ            2.7263477 sec
RG                  362
DW               41.600 usec
DE                6.00 usec
TE               295.3 K
D1           1.00000000 sec
TD0                   1

======== CHANNEL f1 ========
NUC1                  1H
P1                10.90 usec
PL1               -3.00 dB
SFO1        400.1324710 MHz

F2 - Processing parameters
SI                32768
SF          400.1300084 MHz
WDW                  EM
SSB                   0
LB                0.30 Hz
GB                    0
PC                1.00
```

manishkumarmanu1986@gmail.com

4-Amino-2-(2-(2-methoxy-10*H*-phenothiazin-10-yl)-2-oxoethylthio)pyrimidine-5-carbonitrile (23)

MKS3

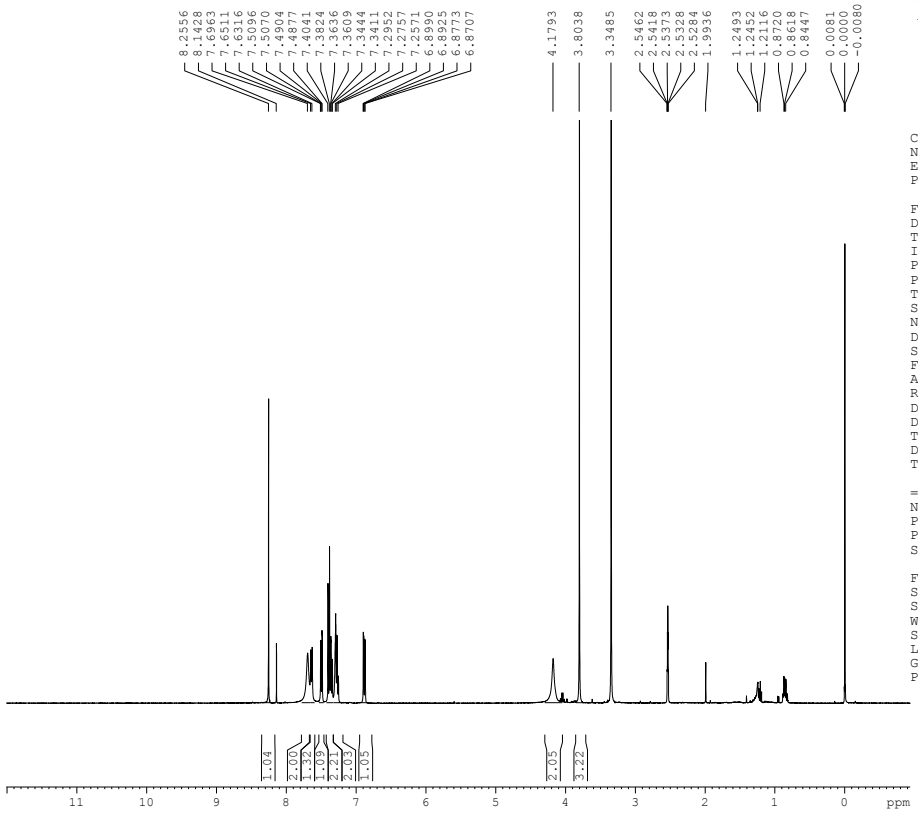

BRUKER
AVANCE II 400 NMR
Spectrometer
SAIF
Panjab University
Chandigarh

Current Data Parameters	
NAME	Mar09-2016
EXPNO	270
PROCNO	1
F2 - Acquisition Parameters	
Date_	20160309
Time	20.42
INSTRUM	spect
PROBHD	5 mm PABBO BB-
PULPROG	zg30
TD	65536
SOLVENT	DMSO
NS	8
DS	2
SWH	12019.230 Hz
FIDRES	0.183399 Hz
AQ	2.7263477 sec
RG	287
DW	41.600 usec
DE	6.00 usec
TE	294.5 K
D1	1.00000000 sec
TD0	1
======== CHANNEL f1 ========	
NUC1	1H
P1	10.90 usec
PL1	-3.00 dB
SFO1	400.1324710 MHz
F2 - Processing parameters	
SI	32768
SF	400.1299888 MHz
WDW	EM
SSB	0
LB	0.30 Hz
GB	0
PC	1.00

manishkumarmanu1986@gmail.com

4-Amino-2-(2-oxo-2-(10*H*-phenothiazin-10-yl)ethylthio)pyrimidine-5-carbonitrile (24)

MKS 04

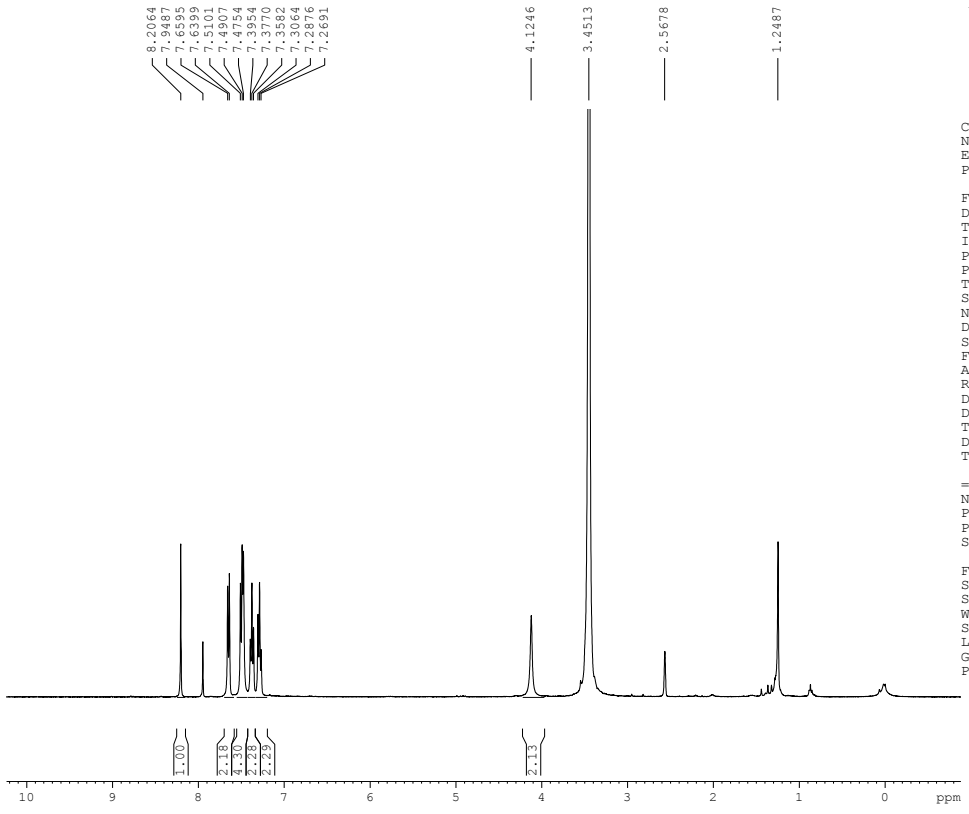

BRUKER
AVANCE II 400 NMR
Spectrometer
SAIF
Panjab University
Chandigarh

Current Data Parameters	
NAME	Sep14-2016
EXPNO	20
PROCNO	1
F2 - Acquisition Parameters	
Date_	20160914
Time	16.37
INSTRUM	spect
PROBHD	5 mm PABBO BB-
PULPROG	zg30
TD	65536
SOLVENT	DMSO
NS	8
DS	2
SWH	12019.230 Hz
FIDRES	0.183399 Hz
AQ	2.7263477 sec
RG	161
DW	41.600 usec
DE	6.00 usec
TE	297.2 K
D1	1.00000000 sec
TD0	1
======== CHANNEL f1 ========	
NUC1	1H
P1	10.90 usec
PL1	-3.00 dB
SFO1	400.1324710 MHz
F2 - Processing parameters	
SI	32768
SF	400.1299765 MHz
WDW	EM
SSB	0
LB	0.30 Hz
GB	0
PC	1.00

manishkumarmanu1986@gmail.com

Ethyl 4-amino-2-(2-(2-trifluoromethyl-10*H*-phenothiazin-10-yl)-2-oxoethylthio)pyrimidine-5-carboxylate (25)

MKS 17

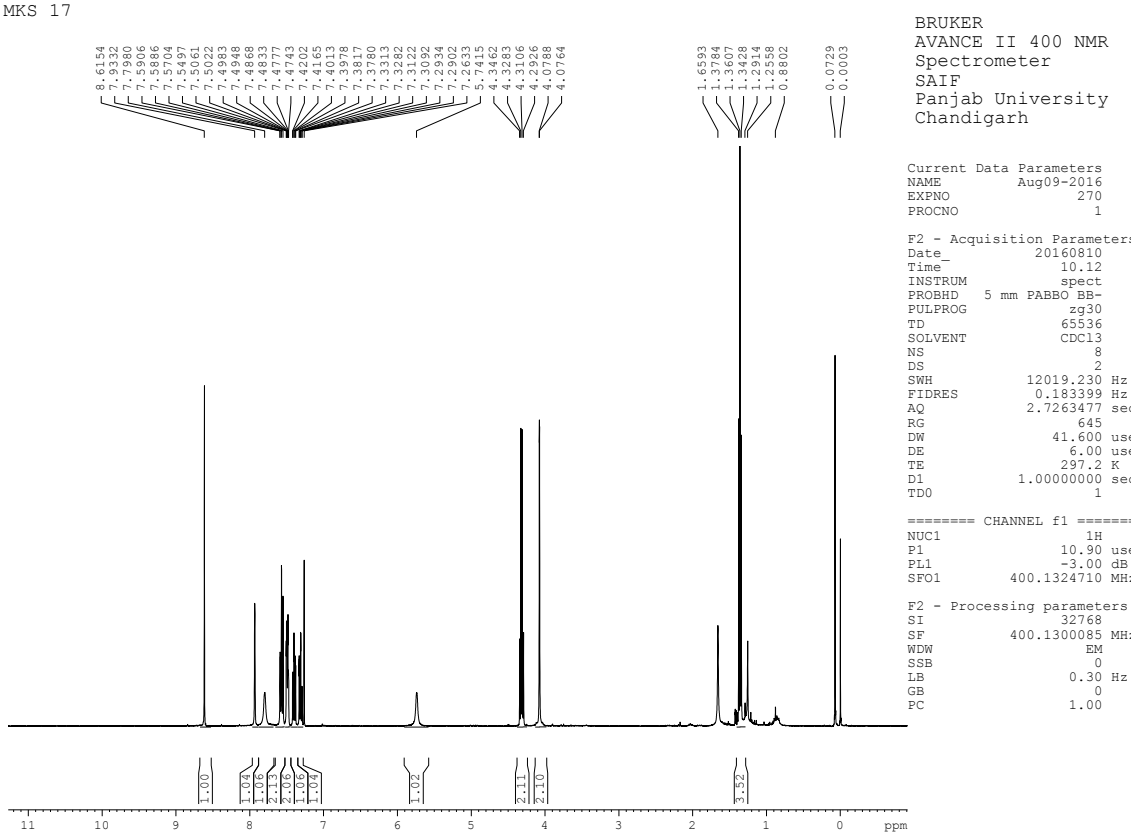

BRUKER
AVANCE II 400 NMR
Spectrometer
SAIF
Panjab University
Chandigarh

```
Current Data Parameters
NAME         Aug09-2016
EXPNO               270
PROCNO                1

F2 - Acquisition Parameters
Date_          20160810
Time              10.12
INSTRUM           spect
PROBHD   5 mm PABBO BB-
PULPROG            zg30
TD                65536
SOLVENT           CDCl3
NS                    8
DS                    2
SWH           12019.230 Hz
FIDRES         0.183399 Hz
AQ            2.7263477 sec
RG                  645
DW               41.600 usec
DE                6.00 usec
TE               297.2 K
D1           1.00000000 sec
TD0                   1

======== CHANNEL f1 ========
NUC1                 1H
P1                10.90 usec
PL1               -3.00 dB
SFO1        400.1324710 MHz

F2 - Processing parameters
SI                32768
SF          400.1300085 MHz
WDW                  EM
SSB                   0
LB                 0.30 Hz
GB                    0
PC                 1.00
```

manishkumarmanu1986@gmail.com

Ethyl 4-amino-2-(2-(2-chloro-10*H*-phenothiazin-10-yl)-2-oxoethylthio)pyrimidine-5-carboxylate (26)

MKS 11

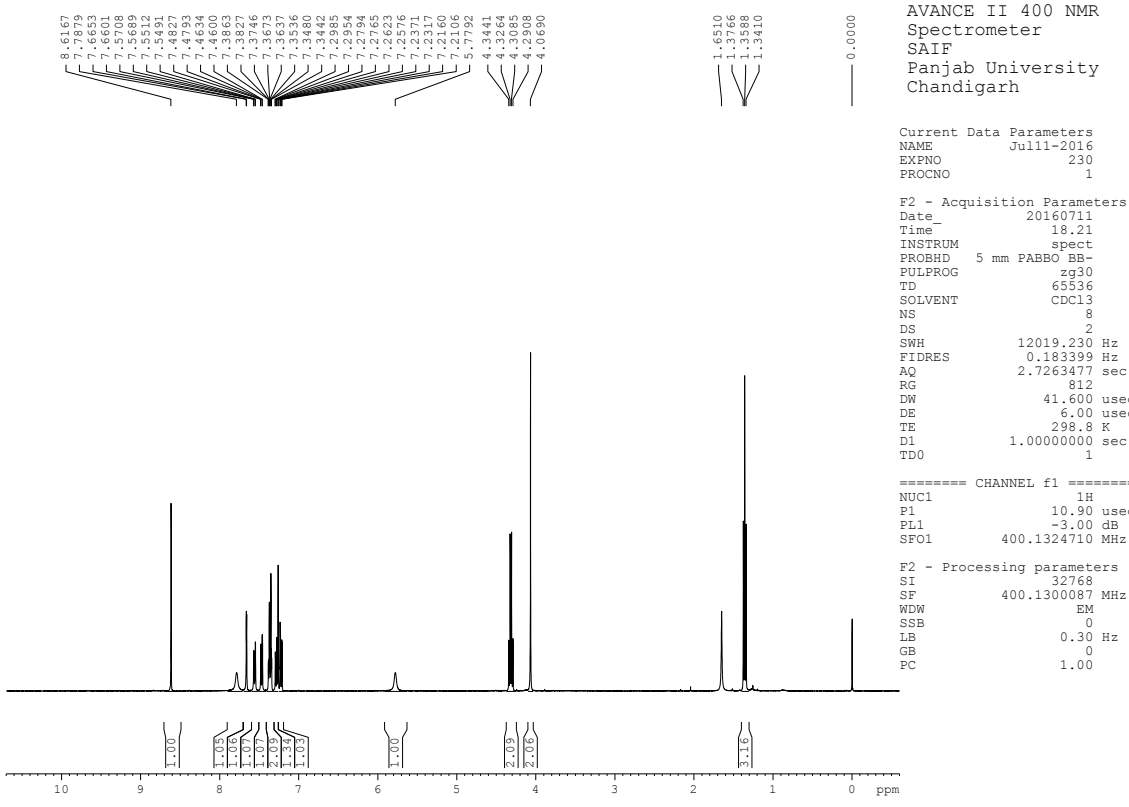

BRUKER
AVANCE II 400 NMR
Spectrometer
SAIF
Panjab University
Chandigarh

```
Current Data Parameters
NAME         Jul11-2016
EXPNO               230
PROCNO                1

F2 - Acquisition Parameters
Date_          20160711
Time              18.21
INSTRUM           spect
PROBHD   5 mm PABBO BB-
PULPROG            zg30
TD                65536
SOLVENT           CDCl3
NS                    8
DS                    2
SWH           12019.230 Hz
FIDRES         0.183399 Hz
AQ            2.7263477 sec
RG                  812
DW               41.600 usec
DE                6.00 usec
TE               298.8 K
D1           1.00000000 sec
TD0                   1

======== CHANNEL f1 ========
NUC1                 1H
P1                10.90 usec
PL1               -3.00 dB
SFO1        400.1324710 MHz

F2 - Processing parameters
SI                32768
SF          400.1300087 MHz
WDW                  EM
SSB                   0
LB                 0.30 Hz
GB                    0
PC                 1.00
```

manishkumarmanu1986@gmail.com

Ethyl 4-amino-2-(2-(2-methoxy-10*H*-phenothiazin-10-yl)-2-oxoethylthio)pyrimidine-5-carboxylate (27)

MKS16

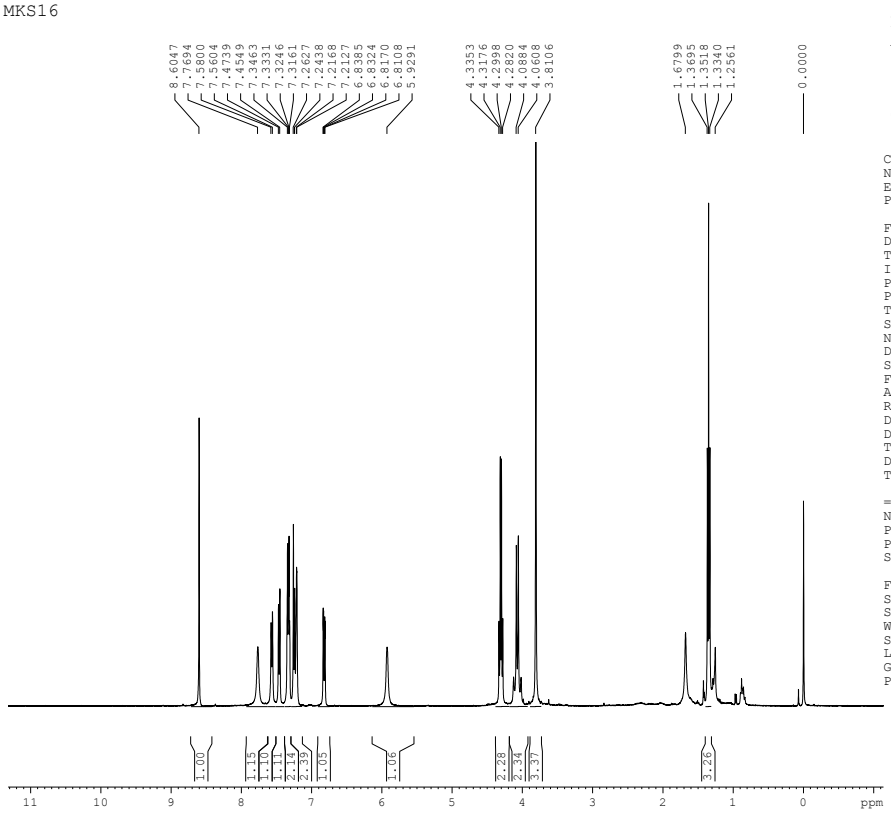

BRUKER
AVANCE II 400 NMR
Spectrometer
SAIF
Panjab University
Chandigarh

```
Current Data Parameters
NAME         Nov29-2016
EXPNO               220
PROCNO                1

F2 - Acquisition Parameters
Date_          20161129
Time              14.23
INSTRUM           spect
PROBHD   5 mm PABBO BB-
PULPROG            zg30
TD                65536
SOLVENT            CDC13
NS                    8
DS                    2
SWH           12019.230 Hz
FIDRES         0.183399 Hz
AQ            2.7263477 sec
RG                  322
DW               41.600 usec
DE                6.00 usec
TE                298.1 K
D1           1.00000000 sec
TD0                   1

======== CHANNEL f1 ========
NUC1                 1H
P1                10.90 usec
PL1               -3.00 dB
SFO1        400.1324710 MHz

F2 - Processing parameters
SI                32768
SF          400.1300083 MHz
WDW                  EM
SSB                   0
LB                 0.30 Hz
GB                    0
PC                 1.00
```

manishkumarmanu1986@gmail.com

Ethyl 4-amino-2-(2-oxo-2-(10*H*-phenothiazin-10-yl)ethylthio)pyrimidine-5-carboxylate (28)

MKS 15

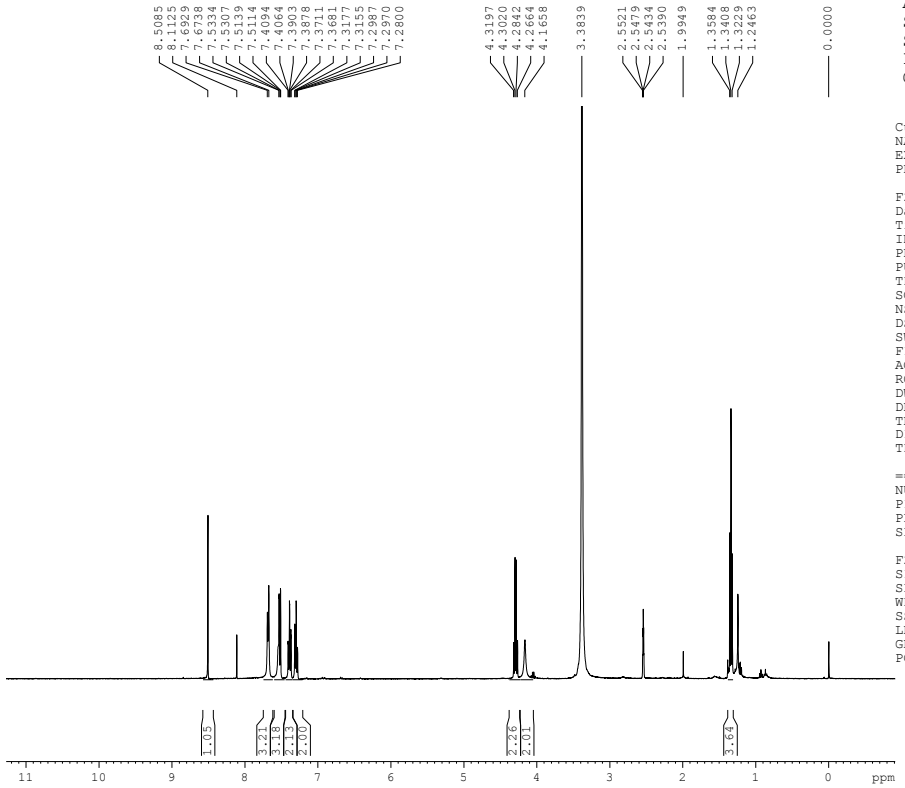

BRUKER
AVANCE II 400 NMR
Spectrometer
SAIF
Panjab University
Chandigarh

```
Current Data Parameters
NAME         Aug09-2016
EXPNO               260
PROCNO                1

F2 - Acquisition Parameters
Date_          20160810
Time              10.06
INSTRUM           spect
PROBHD   5 mm PABBO BB-
PULPROG            zg30
TD                65536
SOLVENT             DMSO
NS                    8
DS                    2
SWH           12019.230 Hz
FIDRES         0.183399 Hz
AQ            2.7263477 sec
RG                  456
DW               41.600 usec
DE                6.00 usec
TE                297.2 K
D1           1.00000000 sec
TD0                   1

======== CHANNEL f1 ========
NUC1                 1H
P1                10.90 usec
PL1               -3.00 dB
SFO1        400.1324710 MHz

F2 - Processing parameters
SI                32768
SF          400.1299867 MHz
WDW                  EM
SSB                   0
LB                 0.30 Hz
GB                    0
PC                 1.00
```

manishkumarmanu1986@gmail.com

2-(5-(4-pyridyl)-1,3,4-oxadiazol-2-ylthio)-1-(2-trifluoromethyl-10*H*-phenothiazin-10-yl)ethanone (29)

MKS 20

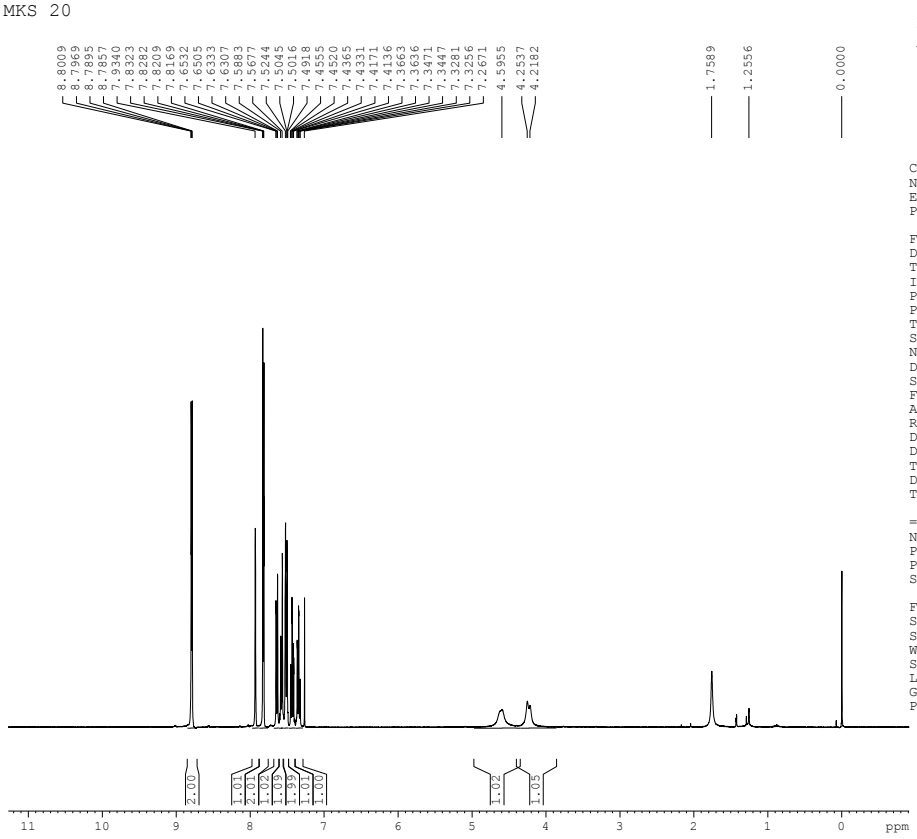

BRUKER
AVANCE II 400 NMR
Spectrometer
SAIF
Panjab University
Chandigarh

```
Current Data Parameters
NAME         Aug09-2016
EXPNO               300
PROCNO                1

F2 - Acquisition Parameters
Date_          20160810
Time              10.31
INSTRUM           spect
PROBHD   5 mm PABBO BB-
PULPROG            zg30
TD                65536
SOLVENT           CDCl3
NS                    8
DS                    2
SWH           12019.230 Hz
FIDRES         0.183399 Hz
AQ            2.7263477 sec
RG                  645
DW               41.600 usec
DE                6.00 usec
TE                297.2 K
D1           1.00000000 sec
TD0                   1

======== CHANNEL f1 ========
NUC1                 1H
P1                10.90 usec
PL1               -3.00 dB
SFO1        400.1324710 MHz

F2 - Processing parameters
SI                32768
SF          400.1300068 MHz
WDW                  EM
SSB                   0
LB                 0.30 Hz
GB                    0
PC                 1.00
```

manishkumarmanu1986@gmail.com

1-(2-Chloro-10*H*-phenothiazin-10-yl)-2-(5-(4-pyridyl)-1,3,4-oxadiazol-2-ylthio)ethanone (30)

MKS 13

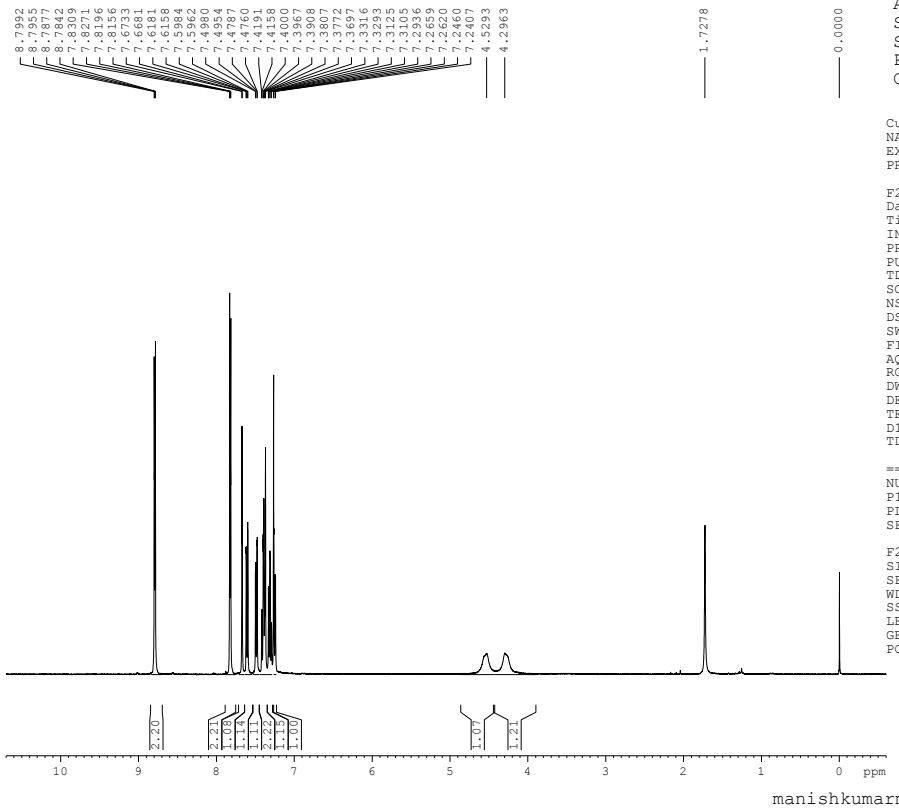

BRUKER
AVANCE II 400 NMR
Spectrometer
SAIF
Panjab University
Chandigarh

```
Current Data Parameters
NAME         Jul11-2016
EXPNO               250
PROCNO                1

F2 - Acquisition Parameters
Date_          20160711
Time              18.32
INSTRUM           spect
PROBHD   5 mm PABBO BB-
PULPROG            zg30
TD                65536
SOLVENT           CDCl3
NS                    8
DS                    2
SWH           12019.230 Hz
FIDRES         0.183399 Hz
AQ            2.7263477 sec
RG                  812
DW               41.600 usec
DE                6.00 usec
TE                298.8 K
D1           1.00000000 sec
TD0                   1

======== CHANNEL f1 ========
NUC1                 1H
P1                10.90 usec
PL1               -3.00 dB
SFO1        400.1324710 MHz

F2 - Processing parameters
SI                32768
SF          400.1300074 MHz
WDW                  EM
SSB                   0
LB                 0.30 Hz
GB                    0
PC                 1.00
```

manishkumarmanu1986@gmail.com

1-(2-Methoxy-10*H*-phenothiazin-10-yl)-2-(5-(4-pyridyl)-1,3,4-oxadiazol-2-ylthio)ethanone (31)

MKS 19

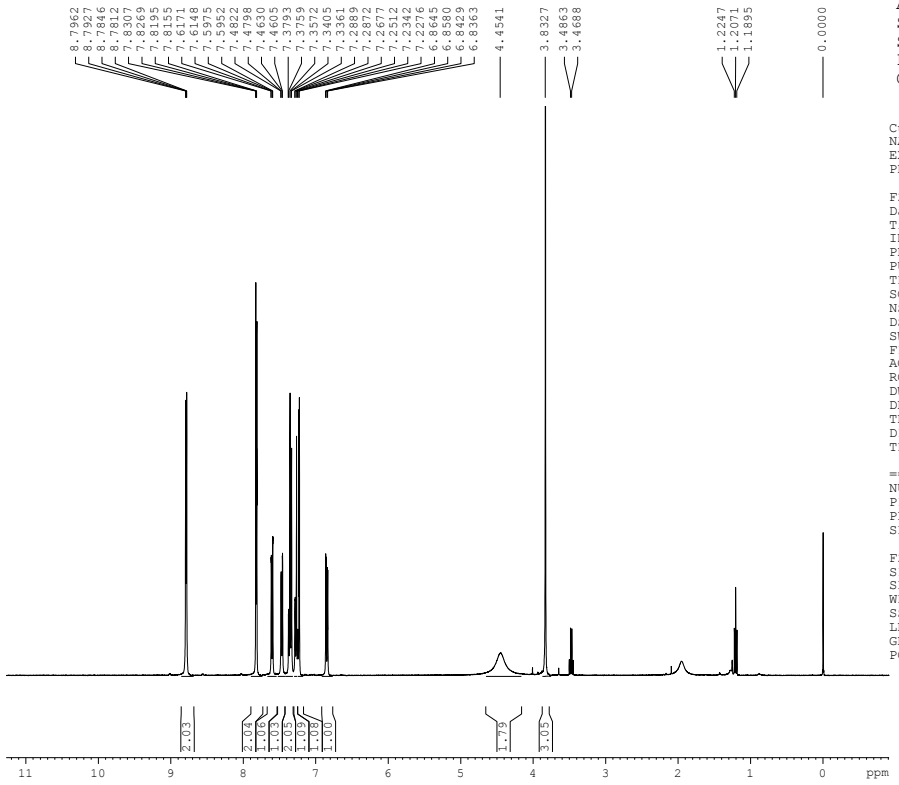

BRUKER
AVANCE II 400 NMR
Spectrometer
SAIF
Panjab University
Chandigarh

```
Current Data Parameters
NAME         Aug09-2016
EXPNO               290
PROCNO                1

F2 - Acquisition Parameters
Date_          20160810
Time              10.25
INSTRUM           spect
PROBHD   5 mm PABBO BB-
PULPROG             zg30
TD                65536
SOLVENT           CDCl3
NS                    8
DS                    2
SWH           12019.230 Hz
FIDRES         0.183399 Hz
AQ            2.7263477 sec
RG                  575
DW               41.600 usec
DE                6.00 usec
TE               297.2 K
D1           1.00000000 sec
TD0                   1

======== CHANNEL f1 ========
NUC1                 1H
P1                10.90 usec
PL1               -3.00 dB
SFO1        400.1324710 MHz

F2 - Processing parameters
SI                32768
SF          400.1300067 MHz
WDW                  EM
SSB                   0
LB                 0.30 Hz
GB                    0
PC                 1.00
```

manishkumarmanu1986@gmail.com

1-(10*H*-phenothiazin-10-yl)-2-(5-(4-pyridyl)-1,3,4-oxadiazol-2-ylthio)ethanone (32)

MKS 18

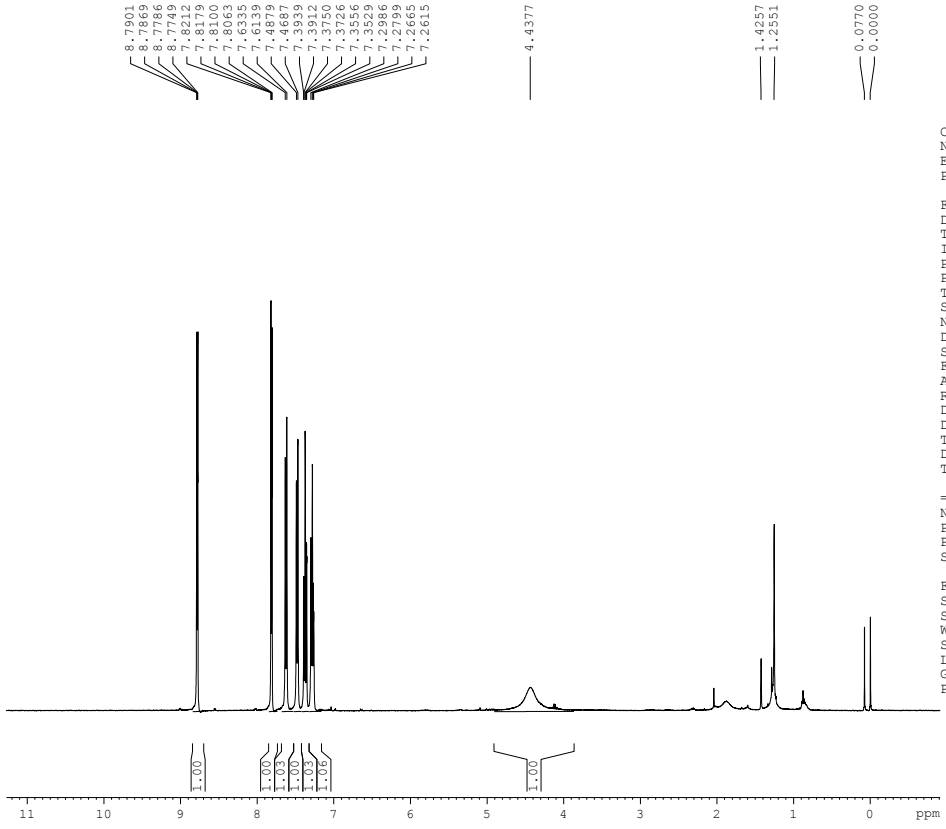

BRUKER
AVANCE II 400 NMR
Spectrometer
SAIF
Panjab University
Chandigarh

```
Current Data Parameters
NAME         Aug09-2016
EXPNO               280
PROCNO                1

F2 - Acquisition Parameters
Date_          20160810
Time              10.20
INSTRUM           spect
PROBHD   5 mm PABBO BB-
PULPROG             zg30
TD                65536
SOLVENT           CDCl3
NS                    8
DS                    2
SWH           12019.230 Hz
FIDRES         0.183399 Hz
AQ            2.7263477 sec
RG                  512
DW               41.600 usec
DE                6.00 usec
TE               297.2 K
D1           1.00000000 sec
TD0                   1

======== CHANNEL f1 ========
NUC1                 1H
P1                10.90 usec
PL1               -3.00 dB
SFO1        400.1324710 MHz

F2 - Processing parameters
SI                32768
SF          400.1300070 MHz
WDW                  EM
SSB                   0
LB                 0.30 Hz
GB                    0
PC                 1.00
```

manishkumarmanu1986@gmail.com

2-(4-Amino-5-(4-pyridyl)-4*H*-1,2,4-triazol-3-ylthio)-1-(2-(trifluoromethyl)-10*H*-phenothiazin-10-yl)ethanone (33)

MKS22

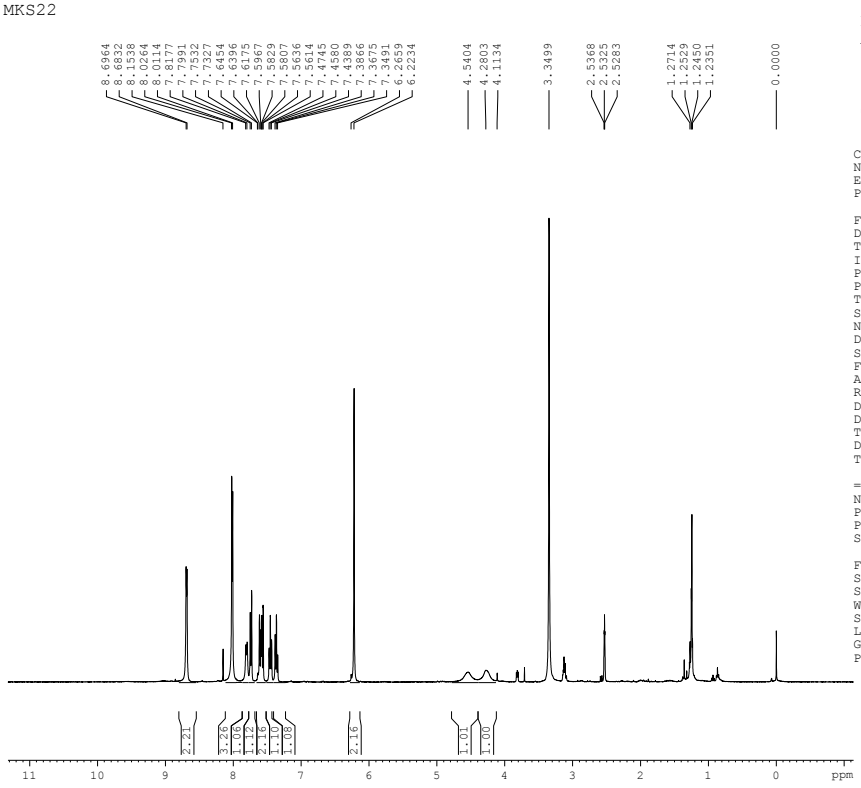

BRUKER
AVANCE II 400 NMR
Spectrometer
SAIF
Panjab University
Chandigarh

Current Data Parameters	
NAME	Nov29-2016
EXPNO	240
PROCNO	1
F2 - Acquisition Parameters	
Date_	20161129
Time	14.33
INSTRUM	spect
PROBHD	5 mm PABBO BB-
PULPROG	zg30
TD	65536
SOLVENT	DMSO
NS	8
DS	2
SWH	12019.230 Hz
FIDRES	0.183399 Hz
AQ	2.7263477 sec
RG	287
DW	41.600 usec
DE	6.00 usec
TE	298.1 K
D1	1.00000000 sec
TD0	1
======== CHANNEL f1 ========	
NUC1	1H
P1	10.90 usec
PL1	-3.00 dB
SFO1	400.1324710 MHz
F2 - Processing parameters	
SI	32768
SF	400.1299902 MHz
WDW	EM
SSB	0
LB	0.30 Hz
GB	0
PC	1.00

manishkumarmanu1986@gmail.com

2-(4-Amino-5-(4-pyridyl)-4*H*-1,2,4-triazol-3-ylthio)-1-(2-chloro-10*H*-phenothiazin-10-yl)ethanone (34)

MKS 14

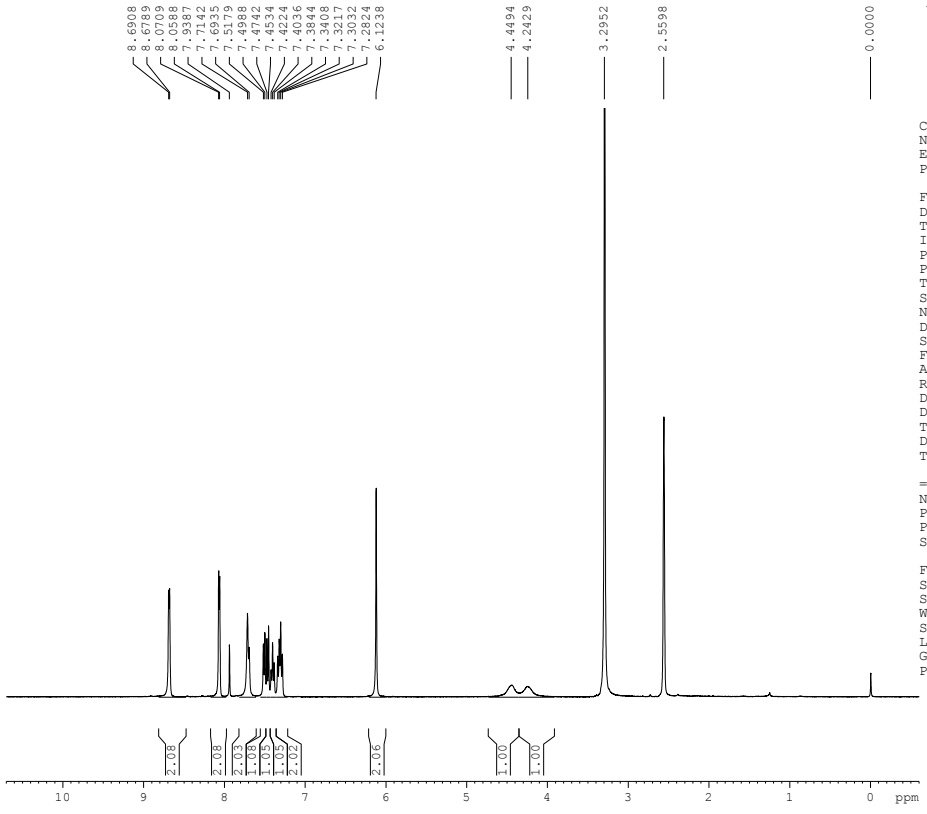

BRUKER
AVANCE II 400 NMR
Spectrometer
SAIF
Panjab University
Chandigarh

Current Data Parameters	
NAME	Jul11-2016
EXPNO	260
PROCNO	1
F2 - Acquisition Parameters	
Date_	20160711
Time	18.38
INSTRUM	spect
PROBHD	5 mm PABBO BB-
PULPROG	zg30
TD	65536
SOLVENT	DMSO
NS	8
DS	2
SWH	12019.230 Hz
FIDRES	0.183399 Hz
AQ	2.7263477 sec
RG	362
DW	41.600 usec
DE	6.00 usec
TE	298.7 K
D1	1.00000000 sec
TD0	1
======== CHANNEL f1 ========	
NUC1	1H
P1	10.90 usec
PL1	-3.00 dB
SFO1	400.1324710 MHz
F2 - Processing parameters	
SI	32768
SF	400.1299803 MHz
WDW	EM
SSB	0
LB	0.30 Hz
GB	0
PC	1.00

manishkumarmanu1986@gmail.com

2-(4-Amino-5-(4-pyridyl)-4*H*-1,2,4-triazol-3-ylthio)-1-(2-methoxy-10*H*-phenothiazin-10-yl)ethanone (35)

MKS21

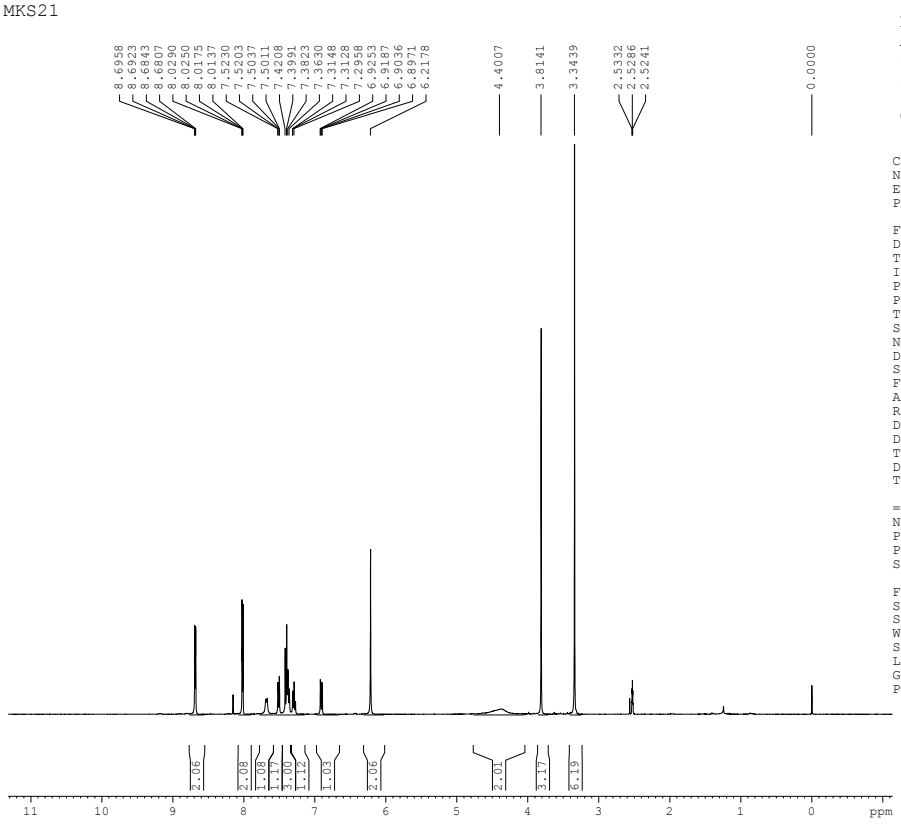

BRUKER
AVANCE II 400 NMR
Spectrometer
SAIF
Panjab University
Chandigarh

Current Data Parameters	
NAME	Nov29-2016
EXPNO	230
PROCNO	1
F2 - Acquisition Parameters	
Date_	20161129
Time	14.28
INSTRUM	spect
PROBHD	5 mm PABBO BB-
PULPROG	zg30
TD	65536
SOLVENT	DMSO
NS	8
DS	2
SWH	12019.230 Hz
FIDRES	0.183399 Hz
AQ	2.7263477 sec
RG	256
DW	41.600 usec
DE	6.00 usec
TE	298.1 K
D1	1.00000000 sec
TD0	1
======== CHANNEL f1 ========	
NUC1	1H
P1	10.90 usec
PL1	-3.00 dB
SFO1	400.1324710 MHz
F2 - Processing parameters	
SI	32768
SF	400.1299918 MHz
WDW	EM
SSB	0
LB	0.30 Hz
GB	0
PC	1.00

manishkumarmanu1986@gmail.com

2-(4-Amino-5-(4-pyridyl)-4*H*-1,2,4-triazol-3-ylthio)-1-(10*H*-phenothiazin-10-yl)ethanone (36)

MKS23

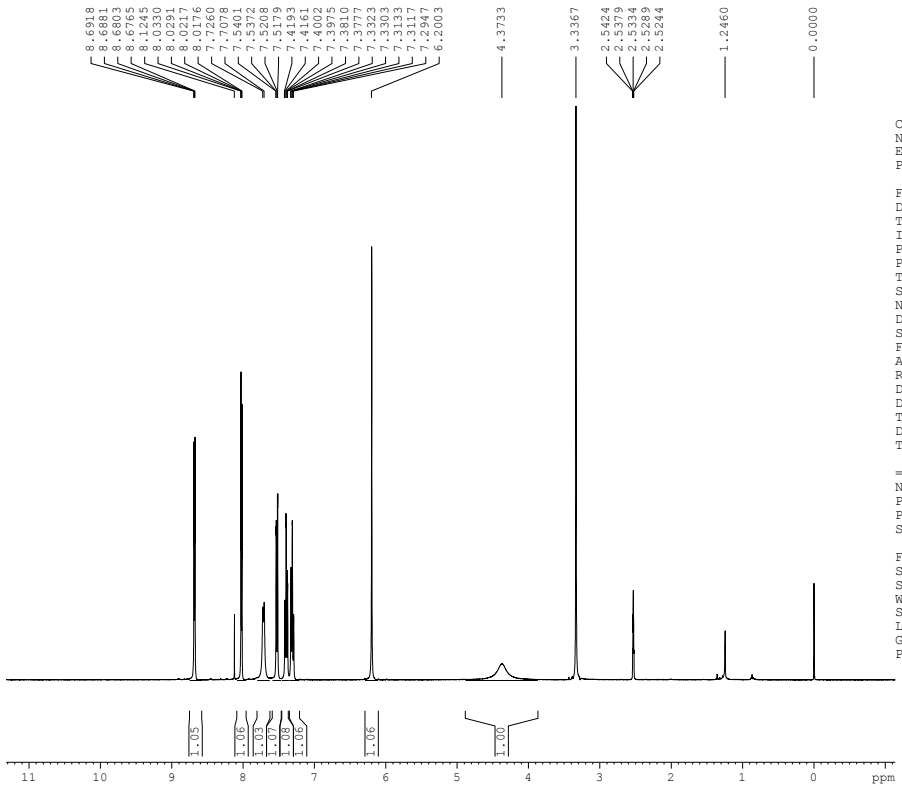

BRUKER
AVANCE II 400 NMR
Spectrometer
SAIF
Panjab University
Chandigarh

Current Data Parameters	
NAME	Nov29-2016
EXPNO	250
PROCNO	1
F2 - Acquisition Parameters	
Date_	20161129
Time	14.38
INSTRUM	spect
PROBHD	5 mm PABBO BB-
PULPROG	zg30
TD	65536
SOLVENT	DMSO
NS	8
DS	2
SWH	12019.230 Hz
FIDRES	0.183399 Hz
AQ	2.7263477 sec
RG	322
DW	41.600 usec
DE	6.00 usec
TE	298.1 K
D1	1.00000000 sec
TD0	1
======== CHANNEL f1 ========	
NUC1	1H
P1	10.90 usec
PL1	-3.00 dB
SFO1	400.1324710 MHz
F2 - Processing parameters	
SI	32768
SF	400.1299900 MHz
WDW	EM
SSB	0
LB	0.30 Hz
GB	0
PC	1.00

manishkumarmanu1986@gmail.com

4-Amino-2-(3-(2-trifluoromethyl-10*H*-phenothiazin-10-yl)propylthio)pyrimidine-5-carbonitrile (42)

MKS-31

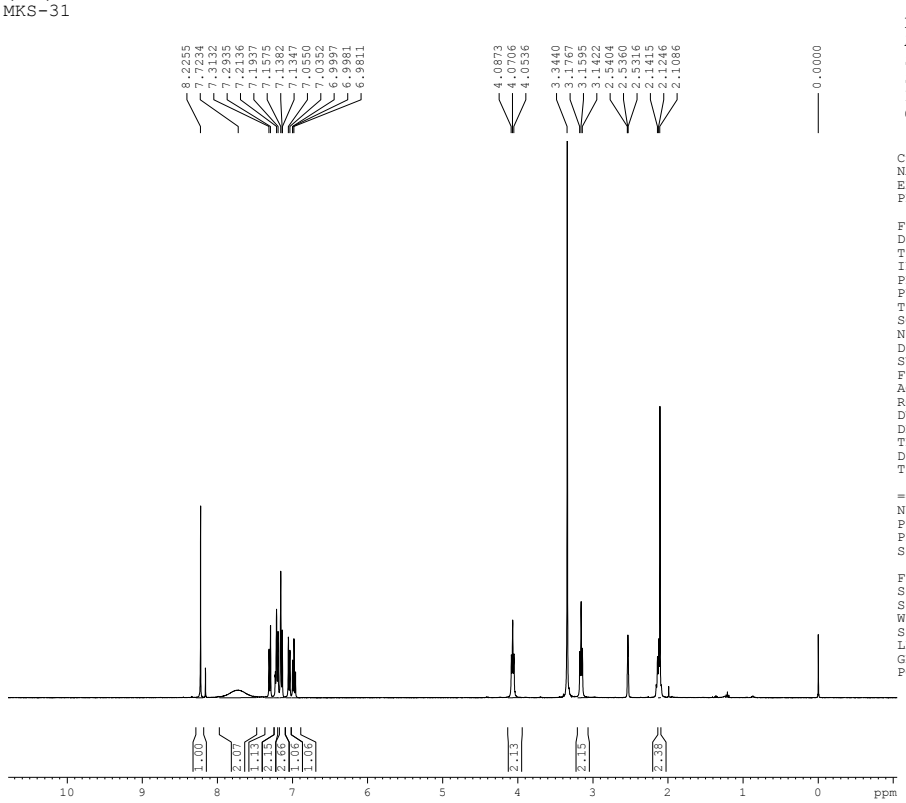

BRUKER
AVANCE II 400 NMR
Spectrometer
SAIF
Panjab University
Chandigarh

Current Data Parameters	
NAME	Dec15-2016
EXPNO	380
PROCNO	1
F2 - Acquisition Parameters	
Date_	20161215
Time	16.33
INSTRUM	spect
PROBHD	5 mm PABBO BB-
PULPROG	zg30
TD	65536
SOLVENT	DMSO
NS	8
DS	2
SWH	12019.230 Hz
FIDRES	0.183399 Hz
AQ	2.7263477 sec
RG	322
DW	41.600 usec
DE	6.00 usec
TE	295.7 K
D1	1.00000000 sec
TD0	1
======== CHANNEL f1 ========	
NUC1	1H
P1	10.90 usec
PL1	-3.00 dB
SFO1	400.1324710 MHz
F2 - Processing parameters	
SI	32768
SF	400.1299889 MHz
WDW	EM
SSB	0
LB	0.30 Hz
GB	0
PC	1.00

manishkumarmanu1986@gmail.com

4-Amino-2-(3-(2-chloro-10*H*-phenothiazin-10-yl)propylthio)pyrimidine-5-carbonitrile (43)

MKS-28

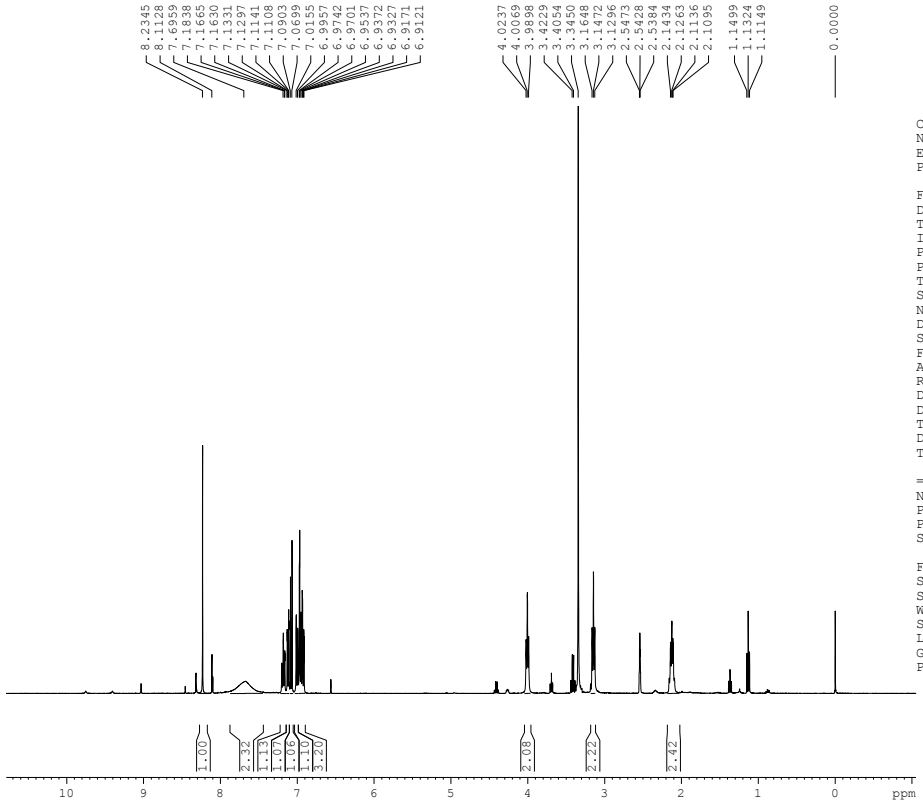

BRUKER
AVANCE II 400 NMR
Spectrometer
SAIF
Panjab University
Chandigarh

Current Data Parameters	
NAME	Dec15-2016
EXPNO	370
PROCNO	1
F2 - Acquisition Parameters	
Date_	20161215
Time	16.28
INSTRUM	spect
PROBHD	5 mm PABBO BB-
PULPROG	zg30
TD	65536
SOLVENT	DMSO
NS	8
DS	2
SWH	12019.230 Hz
FIDRES	0.183399 Hz
AQ	2.7263477 sec
RG	287
DW	41.600 usec
DE	6.00 usec
TE	295.7 K
D1	1.00000000 sec
TD0	1
======== CHANNEL f1 ========	
NUC1	1H
P1	10.90 usec
PL1	-3.00 dB
SFO1	400.1324710 MHz
F2 - Processing parameters	
SI	32768
SF	400.1299863 MHz
WDW	EM
SSB	0
LB	0.30 Hz
GB	0
PC	1.00

manishkumarmanu1986@gmail.com

4-Amino-2-(3-(2-methoxy-10*H*-phenothiazin-10-yl)propylthio)pyrimidine-5-carbonitrile (44)

MKS-30

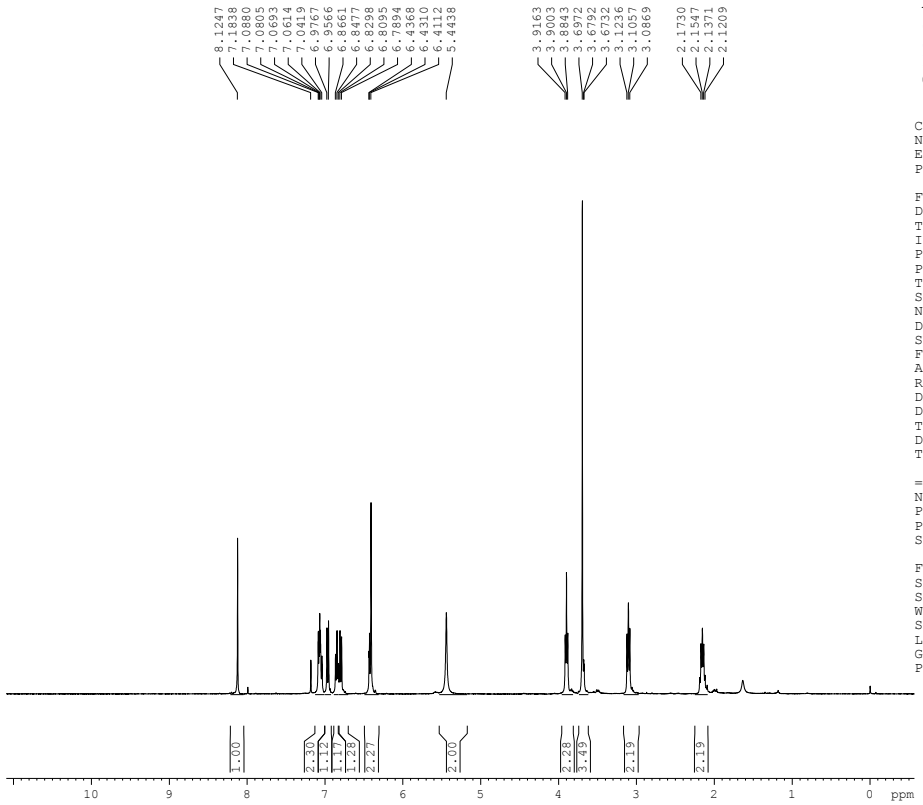

BRUKER
AVANCE II 400 NMR
Spectrometer
SAIF
Panjab University
Chandigarh

Current Data Parameters	
NAME	Dec08-2016
EXPNO	80
PROCNO	1
**F2 - Acquisition Parameters**	
Date_	20161208
Time	17.15
INSTRUM	spect
PROBHD	5 mm PABBO BB-
PULPROG	zg30
TD	65536
SOLVENT	CDCl3
NS	8
DS	2
SWH	12019.230 Hz
FIDRES	0.183399 Hz
AQ	2.7263477 sec
RG	406
DW	41.600 usec
DE	6.00 usec
TE	296.7 K
D1	1.00000000 sec
TD0	1
**======== CHANNEL f1 ========**	
NUC1	1H
P1	10.90 usec
PL1	-3.00 dB
SFO1	400.1324710 MHz
**F2 - Processing parameters**	
SI	32768
SF	400.1300399 MHz
WDW	EM
SSB	0
LB	0.30 Hz
GB	0
PC	1.00

manishkumarmanu1986@gmail.com

4-Amino-2-(3-(10*H*-phenothiazin-10-yl)propylthio)pyrimidine-5-carbonitrile (45)

MKS 12

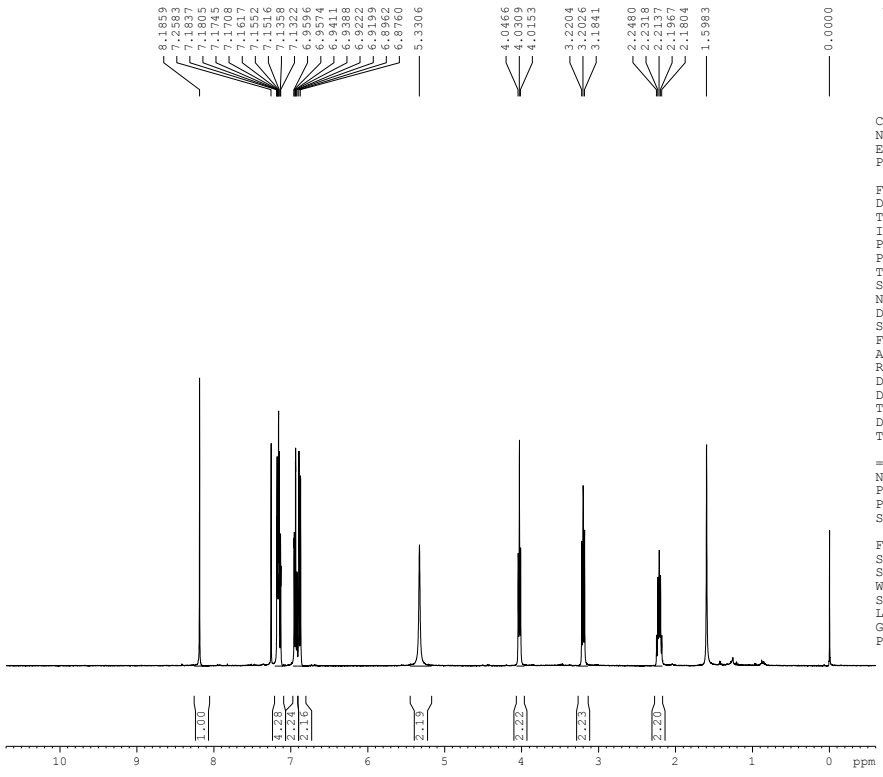

BRUKER
AVANCE II 400 NMR
Spectrometer
SAIF
Panjab University
Chandigarh

Current Data Parameters	
NAME	Jul11-2016
EXPNO	240
PROCNO	1
**F2 - Acquisition Parameters**	
Date_	20160711
Time	18.27
INSTRUM	spect
PROBHD	5 mm PABBO BB-
PULPROG	zg30
TD	65536
SOLVENT	CDCl3
NS	8
DS	2
SWH	12019.230 Hz
FIDRES	0.183399 Hz
AQ	2.7263477 sec
RG	456
DW	41.600 usec
DE	6.00 usec
TE	298.8 K
D1	1.00000000 sec
TD0	1
**======== CHANNEL f1 ========**	
NUC1	1H
P1	10.90 usec
PL1	-3.00 dB
SFO1	400.1324710 MHz
**F2 - Processing parameters**	
SI	32768
SF	400.1300104 MHz
WDW	EM
SSB	0
LB	0.30 Hz
GB	0
PC	1.00

manishkumarmanu1986@gmail.com

Ethyl 4-amino-2-(3-(2-trifluoromethyl-10*H*-phenothiazin-10-yl)propylthio)pyrimidine-5-carboxylate (46)

MKS 24

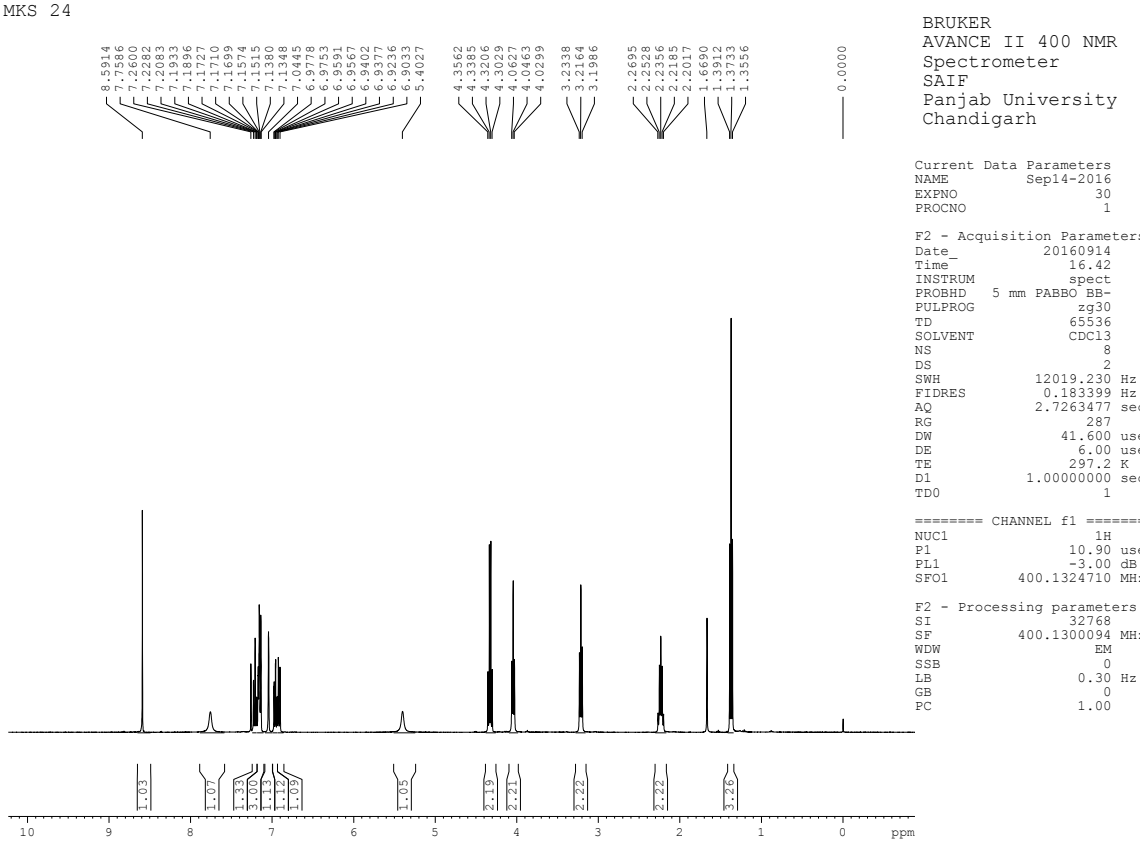

manishkumarmanu1986@gmail.com

Ethyl 4-amino-2-(3-(2-chloro-10*H*-phenothiazin-10-yl)propylthio)pyrimidine-5-carboxylate (47)

MKS-27

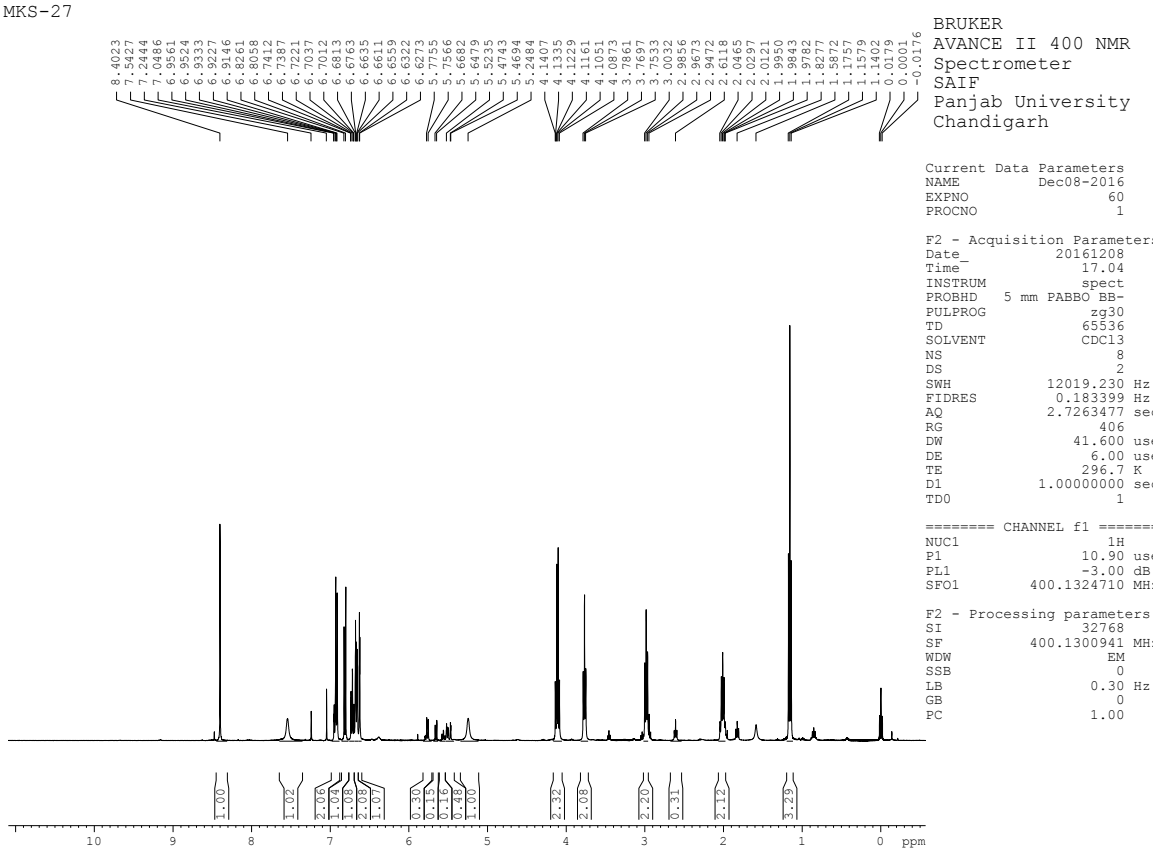

manishkumarmanu1986@gmail.com

Ethyl 4-amino-2-(3-(2-methoxy-10*H*-phenothiazin-10-yl)propylthio)pyrimidine-5-carboxylate (48)

MKS-29

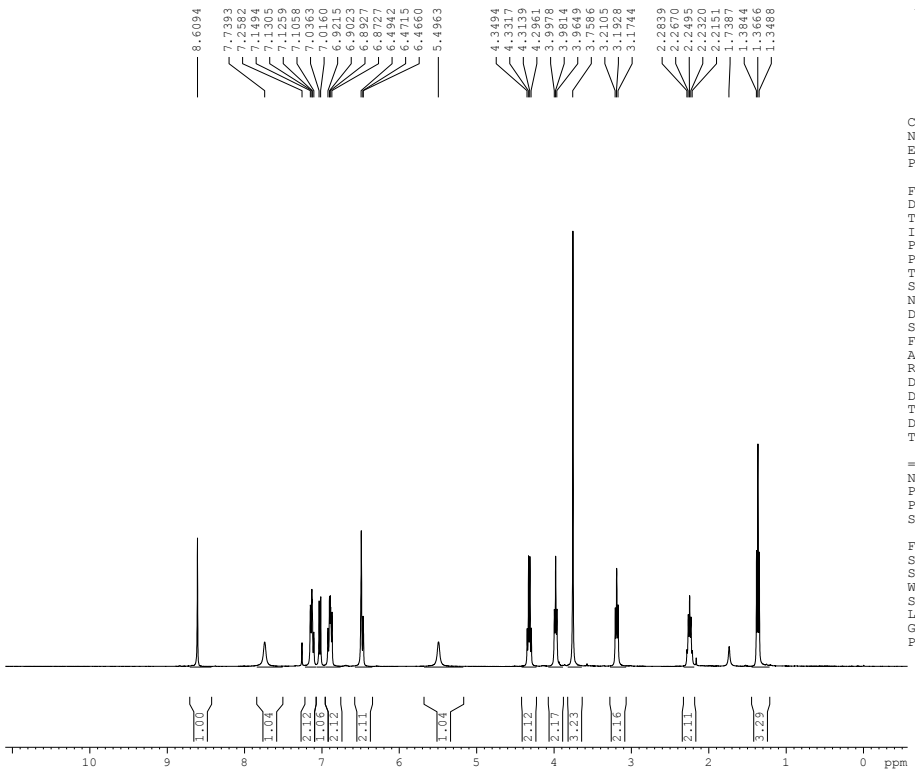


```
BRUKER
AVANCE II 400 NMR
Spectrometer
SAIF
Panjab University
Chandigarh

Current Data Parameters
NAME         Dec08-2016
EXPNO                70
PROCNO                1

F2 - Acquisition Parameters
Date_          20161208
Time              17.10
INSTRUM           spect
PROBHD   5 mm PABBO BB-
PULPROG            zg30
TD                65536
SOLVENT           CDCl3
NS                    8
DS                    2
SWH           12019.230 Hz
FIDRES         0.183399 Hz
AQ            2.7263477 sec
RG                  203
DW               41.600 usec
DE                6.00 usec
TE                296.7 K
D1           1.00000000 sec
TD0                   1

======== CHANNEL f1 ========
NUC1                  1H
P1                10.90 usec
PL1               -3.00 dB
SFO1        400.1324710 MHz

F2 - Processing parameters
SI                32768
SF          400.1300100 MHz
WDW                  EM
SSB                   0
LB                 0.30 Hz
GB                    0
PC                 1.00
```

manishkumarmanu1986@gmail.com

Ethyl 4-amino-2-(3-(10*H*-phenothiazin-10-yl)propylthio)pyrimidine-5-carboxylate (49)

MKS-27

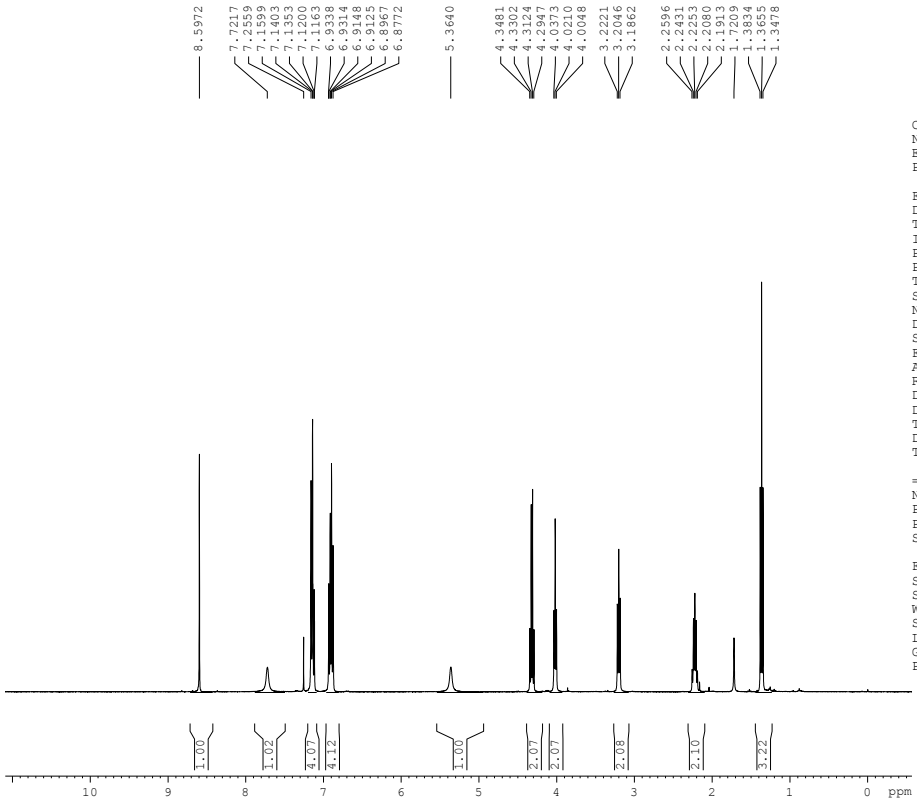


```
BRUKER
AVANCE II 400 NMR
Spectrometer
SAIF
Panjab University
Chandigarh

Current Data Parameters
NAME         Dec08-2016
EXPNO                50
PROCNO                1

F2 - Acquisition Parameters
Date_          20161208
Time              16.59
INSTRUM           spect
PROBHD   5 mm PABBO BB-
PULPROG            zg30
TD                65536
SOLVENT           CDCl3
NS                    8
DS                    2
SWH           12019.230 Hz
FIDRES         0.183399 Hz
AQ            2.7263477 sec
RG                  406
DW               41.600 usec
DE                6.00 usec
TE                296.7 K
D1           1.00000000 sec
TD0                   1

======== CHANNEL f1 ========
NUC1                  1H
P1                10.90 usec
PL1               -3.00 dB
SFO1        400.1324710 MHz

F2 - Processing parameters
SI                32768
SF          400.1300111 MHz
WDW                  EM
SSB                   0
LB                 0.30 Hz
GB                    0
PC                 1.00
```

manishkumarmanu1986@gmail.com

^{13}C-NMR Spectra.

4-Amino-2-(2-(2-trifluoromethyl-10*H*-phenothiazin-10-yl)-2-oxoethylthio)pyrimidine-5-carbonitrile (5)

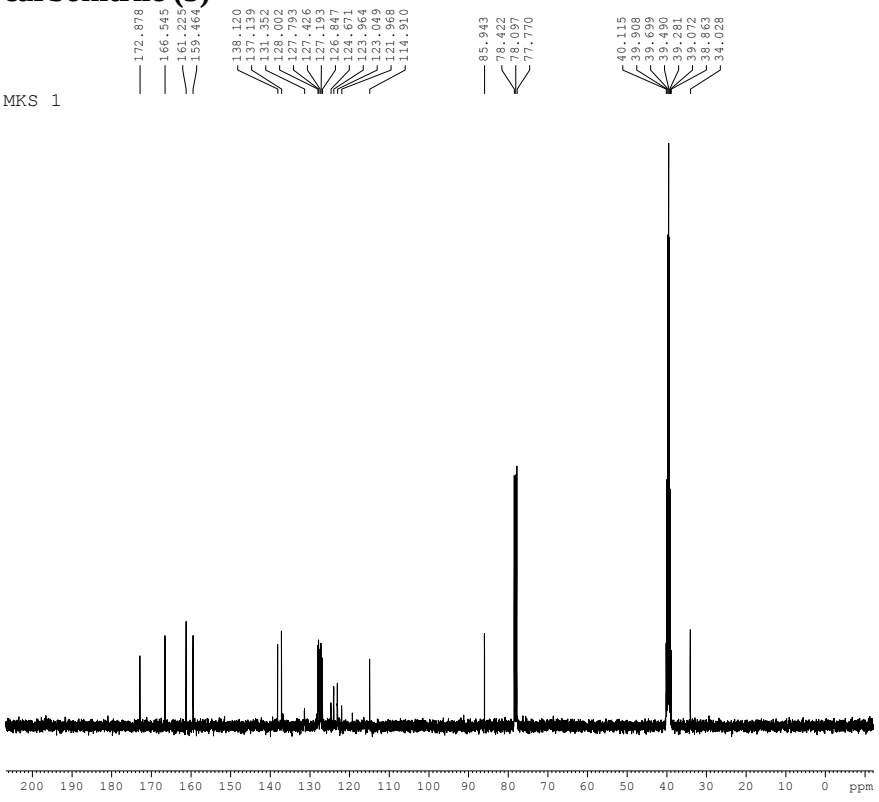


```
   BRUKER
   AVANCE II 400 NMR
   Spectrometer
   SAIF
   Panjab University
   Chandigarh
Current Data Parameters
NAME          Jul04-2017
EXPNO                242
PROCNO                 1

F2 - Acquisition Parameters
Date_           20170705
Time                7.19
INSTRUM            spect
PROBHD   5 mm PABBO BB-
PULPROG           zgpg30
TD                 65536
SOLVENT             DMSO
NS                   512
DS                     4
SWH            29761.904 Hz
FIDRES          0.454131 Hz
AQ             1.1010048 sec
RG                  1290
DW                16.800 usec
DE                 6.50 usec
TE                296.3 K
D1            2.00000000 sec
D11           0.03000000 sec
TD0                    1

======== CHANNEL f1 ========
SFO1         100.6228293 MHz
NUC1                 13C
P1                  9.91 usec
PLW1        41.59999847 W

======== CHANNEL f2 ========
SFO2         400.1316005 MHz
NUC2                 1H
CPDPRG[2         waltz16
PCPD2              90.00 usec
PLW2          6.80000019 W
PLW12         0.22578999 W
PLW13         0.11357000 W

F2 - Processing parameters
SI                  32768
SF           100.6128275 MHz
WDW                  EM
SSB       0
LB                1.00 Hz
GB        0
PC                1.40
```

manishkumarmanu1986@gmail.com

4-Amino-2-(2-(2-methoxy-10*H*-phenothiazin-10-yl)-2-oxoethylthio)pyrimidine-5-carbonitrile (23)

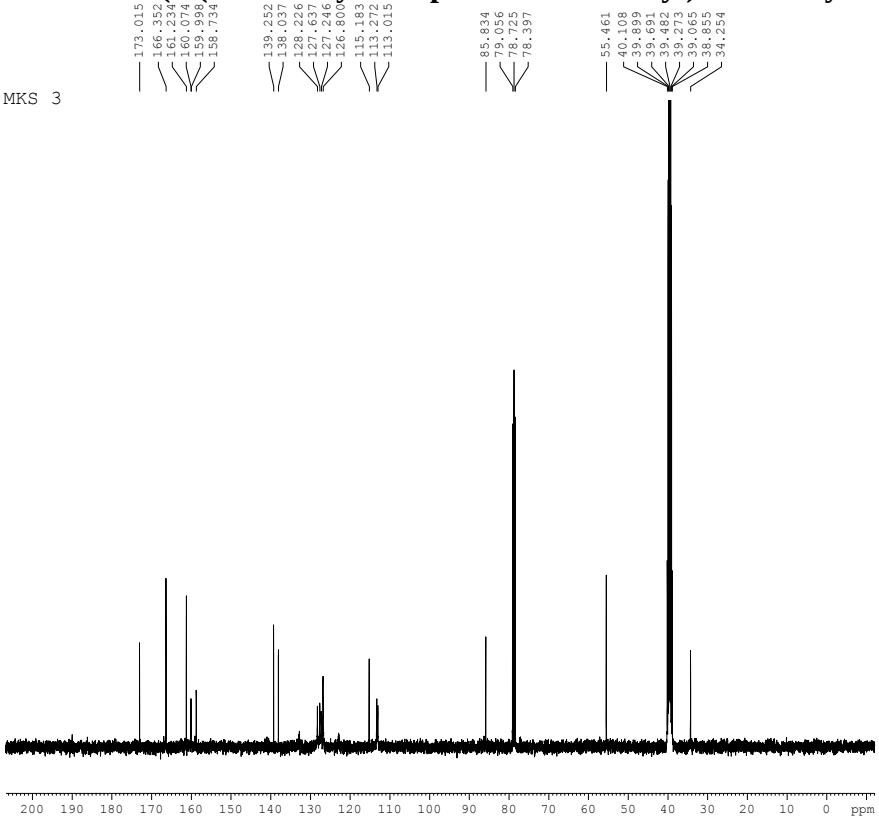


```
   BRUKER
   AVANCE II 400 NMR
   Spectrometer
   SAIF
   Panjab University
   Chandigarh
Current Data Parameters
NAME          Jul04-2017
EXPNO                251
PROCNO                 1

F2 - Acquisition Parameters
Date_           20170705
Time                7.53
INSTRUM            spect
PROBHD   5 mm PABBO BB-
PULPROG           zgpg30
TD                 65536
SOLVENT             CDCl3
NS                   512
DS                     4
SWH            29761.904 Hz
FIDRES          0.454131 Hz
AQ             1.1010048 sec
RG                  1290
DW                16.800 usec
DE                 6.50 usec
TE                296.0 K
D1            2.00000000 sec
D11           0.03000000 sec
TD0                    1

======== CHANNEL f1 ========
SFO1         100.6228293 MHz
NUC1                 13C
P1                  9.91 usec
PLW1        41.59999847 W

======== CHANNEL f2 ========
SFO2         400.1316005 MHz
NUC2                 1H
CPDPRG[2         waltz16
PCPD2              90.00 usec
PLW2          6.80000019 W
PLW12         0.22578999 W
PLW13         0.11357000 W

F2 - Processing parameters
SI                  32768
SF           100.6133014 MHz
WDW                  EM
SSB       0
LB                1.00 Hz
GB        0
PC                1.40
```

manishkumarmanu1986@gmail.com

Ethyl 4-amino-2-(2-(2-chloro-10*H*-phenothiazin-10-yl)-2-oxoethylthio)pyrimidine-5-carboxylate (26)

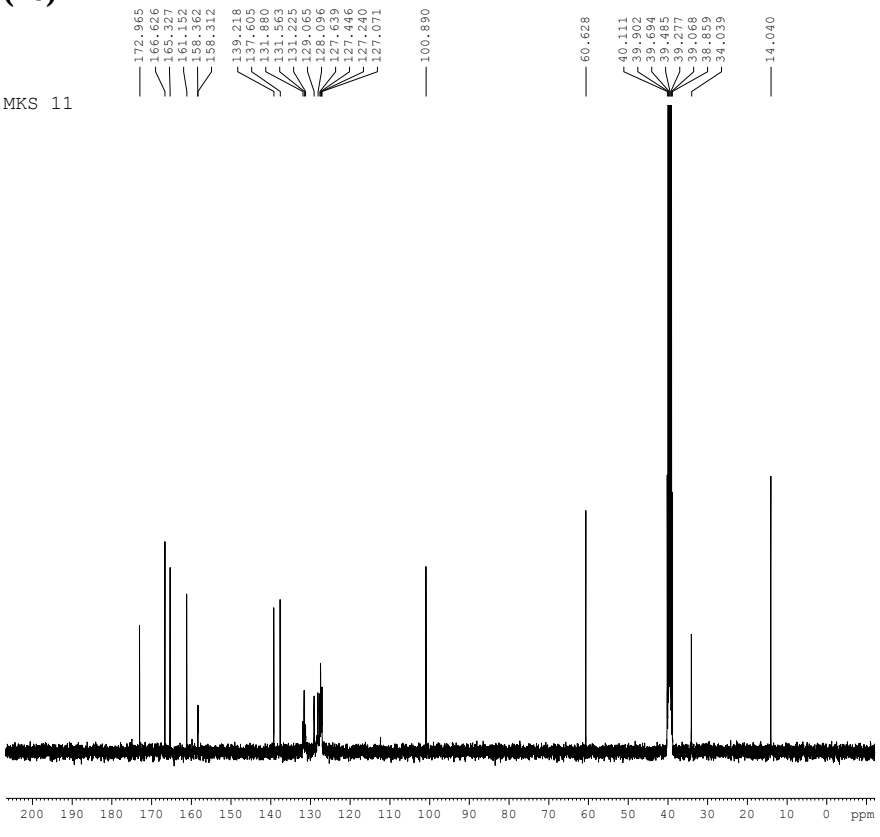


```
   BRUKER
   AVANCE II 400 NMR
   Spectrometer
   SAIF
   Panjab University
   Chandigarh
Current Data Parameters
NAME         Jul04-2017
EXPNO               261
PROCNO                1

F2 - Acquisition Parameters
Date_           20170705
Time                8.24
INSTRUM            spect
PROBHD   5 mm PABBO BB-
PULPROG          zgpg30
TD                65536
SOLVENT           CDCl3
NS                  512
DS                    4
SWH           29761.904 Hz
FIDRES         0.454131 Hz
AQ             1.1010048 sec
RG                 1440
DW                16.800 usec
DE                 6.50 usec
TE                295.9 K
D1            2.00000000 sec
D11           0.03000000 sec
TD0                   1

======== CHANNEL f1 ========
SFO1         100.6228293 MHz
NUC1                 13C
P1                  9.91 usec
PLW1         41.59999847 W

======== CHANNEL f2 ========
SFO2         400.1316005 MHz
NUC2                 1H
CPDPRG[2         waltz16
PCPD2              90.00 usec
PLW2          6.80000019 W
PLW12         0.22578999 W
PLW13         0.11357000 W

F2 - Processing parameters
SI                  32768
SF           100.6132956 MHz
WDW                    EM
SSB       0
LB                  1.00 Hz
GB        0
PC                  1.40
```

manishkumarmanu1986@gmail.com

2-(5-(4-pyridyl)-1,3,4-oxadiazol-2-ylthio)-1-(2-trifluoromethyl-10*H*-phenothiazin-10-yl)ethanone (29)

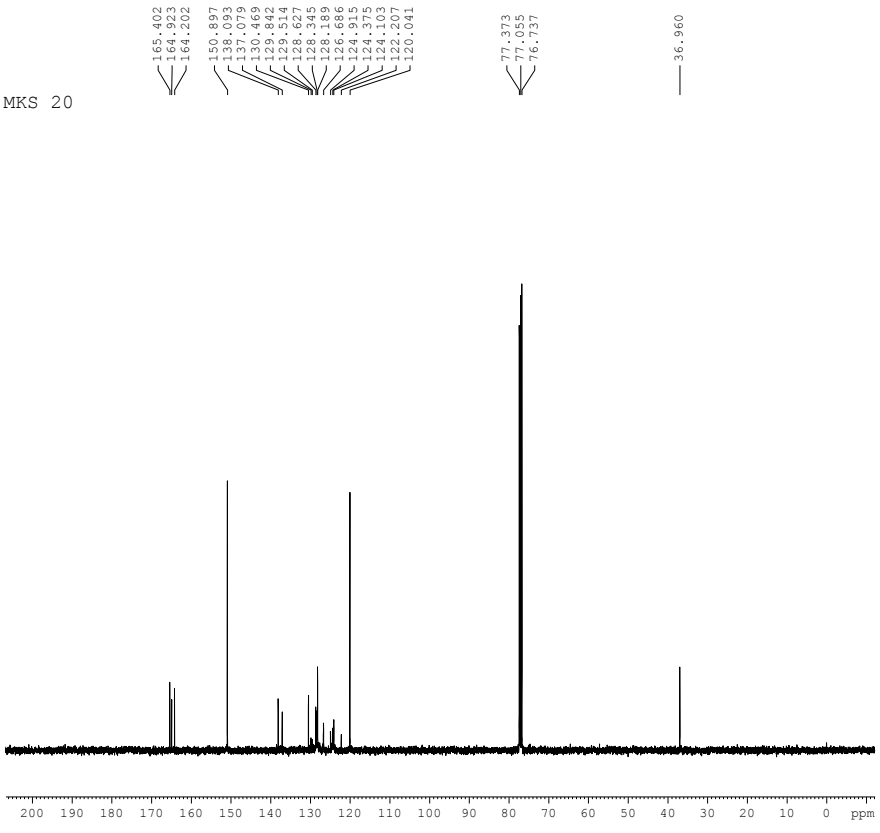


```
   BRUKER
   AVANCE II 400 NMR
   Spectrometer
   SAIF
   Panjab University
   Chandigarh
Current Data Parameters
NAME         Jul04-2017
EXPNO               311
PROCNO                1

F2 - Acquisition Parameters
Date_           20170705
Time               10.58
INSTRUM            spect
PROBHD   5 mm PABBO BB-
PULPROG          zgpg30
TD                65536
SOLVENT           CDCl3
NS                  469
DS                    4
SWH           29761.904 Hz
FIDRES         0.454131 Hz
AQ             1.1010048 sec
RG                 1440
DW                16.800 usec
DE                 6.50 usec
TE                296.9 K
D1            2.00000000 sec
D11           0.03000000 sec
TD0                   1

======== CHANNEL f1 ========
SFO1         100.6228293 MHz
NUC1                 13C
P1                  9.91 usec
PLW1         41.59999847 W

======== CHANNEL f2 ========
SFO2         400.1316005 MHz
NUC2                 1H
CPDPRG[2         waltz16
PCPD2              90.00 usec
PLW2          6.80000019 W
PLW12         0.22578999 W
PLW13         0.11357000 W

F2 - Processing parameters
SI                  32768
SF           100.6127685 MHz
WDW                    EM
SSB       0
LB                  1.00 Hz
GB        0
PC                  1.40
```

manishkumarmanu1986@gmail.com

4-Amino-2-(3-(2-methoxy-10*H*-phenothiazin-10-yl)propylthio)pyrimidine-5-carbonitrile (44)

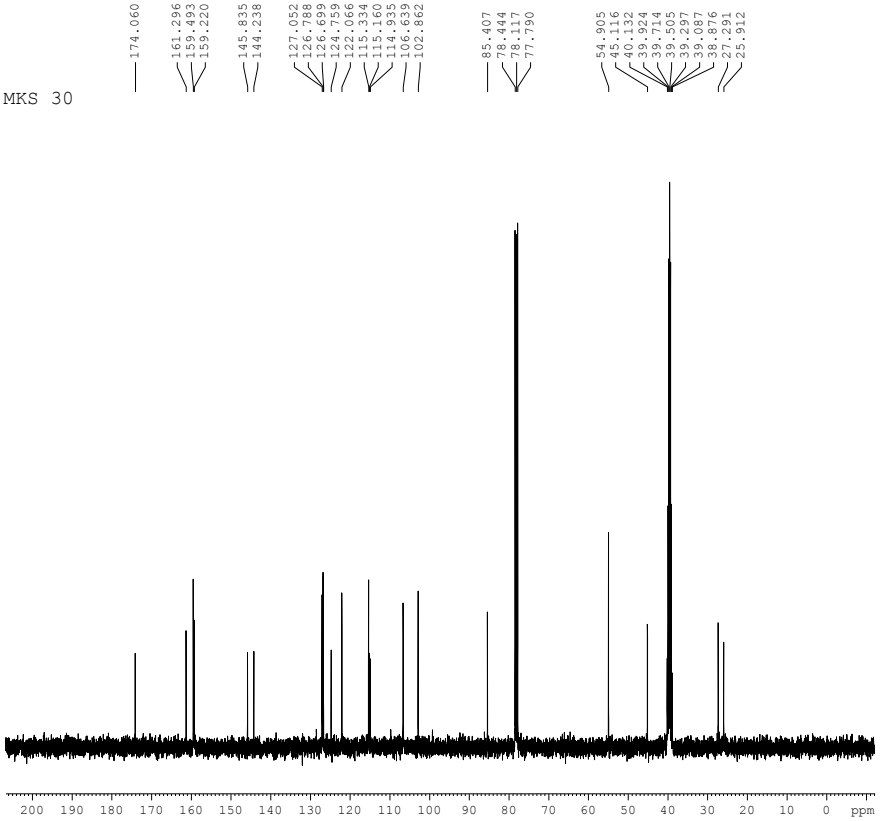


```
   BRUKER
   AVANCE II 400 NMR
   Spectrometer
   SAIF
   Panjab University
   Chandigarh
Current Data Parameters
NAME          Jul04-2017
EXPNO                351
PROCNO                 1

F2 - Acquisition Parameters
Date_           20170705
Time_              15.42
INSTRUM             spect
PROBHD   5 mm PABBO BB-
PULPROG           zgpg30
TD                 65536
SOLVENT             DMSO
NS                   218
DS                     4
SWH            29761.904 Hz
FIDRES          0.454131 Hz
AQ             1.1010048 sec
RG                  1440
DW                16.800 usec
DE                 6.50 usec
TE                297.1 K
D1            2.00000000 sec
D11           0.03000000 sec
TD0                    1

======== CHANNEL f1 ========
SFO1         100.6228293 MHz
NUC1               13C
P1                9.91 usec
PLW1         41.59999847 W

======== CHANNEL f2 ========
SFO2         400.1316005 MHz
NUC2               1H
CPDPRG[2         waltz16
PCPD2             90.00 usec
PLW2          6.80000019 W
PLW12         0.22578999 W
PLW13         0.11357000 W

F2 - Processing parameters
SI                 32768
SF           100.6122895 MHz
WDW                  EM
SSB       0
LB                1.00 Hz
GB        0
PC                1.40
```

manishkumarmanu1986@gmail.com

4-Amino-2-(3-(10*H*-phenothiazin-10-yl)propylthio)pyrimidine-5-carbonitrile (45)

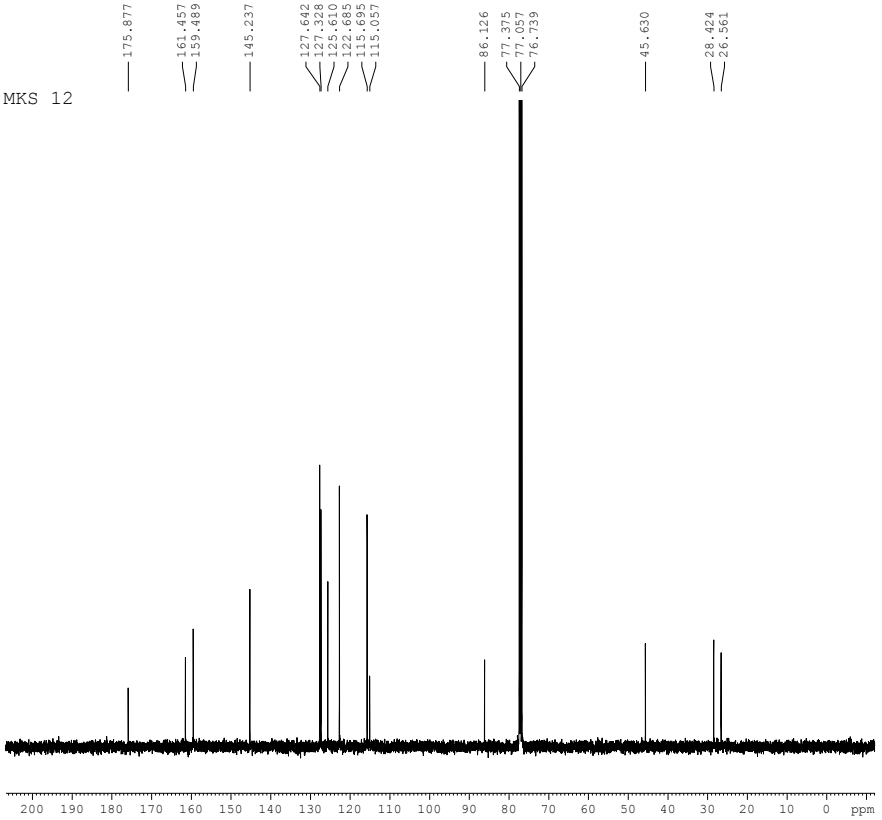


```
   BRUKER
   AVANCE II 400 NMR
   Spectrometer
   SAIF
   Panjab University
   Chandigarh
Current Data Parameters
NAME          Jul04-2017
EXPNO                271
PROCNO                 1

F2 - Acquisition Parameters
Date_           20170705
Time_               8.56
INSTRUM             spect
PROBHD   5 mm PABBO BB-
PULPROG           zgpg30
TD                 65536
SOLVENT            CDCl3
NS                   512
DS                     4
SWH            29761.904 Hz
FIDRES          0.454131 Hz
AQ             1.1010048 sec
RG                  1290
DW                16.800 usec
DE                 6.50 usec
TE                296.0 K
D1            2.00000000 sec
D11           0.03000000 sec
TD0                    1

======== CHANNEL f1 ========
SFO1         100.6228293 MHz
NUC1               13C
P1                9.91 usec
PLW1         41.59999847 W

======== CHANNEL f2 ========
SFO2         400.1316005 MHz
NUC2               1H
CPDPRG[2         waltz16
PCPD2             90.00 usec
PLW2          6.80000019 W
PLW12         0.22578999 W
PLW13         0.11357000 W

F2 - Processing parameters
SI                 32768
SF           100.6127685 MHz
WDW                  EM
SSB       0
LB                1.00 Hz
GB        0
PC                1.40
```

manishkumarmanu1986@gmail.com

Ethyl 4-amino-2-(3-(2-methoxy-10*H*-phenothiazin-10-yl)propylthio)pyrimidine-5-carboxylate (48)

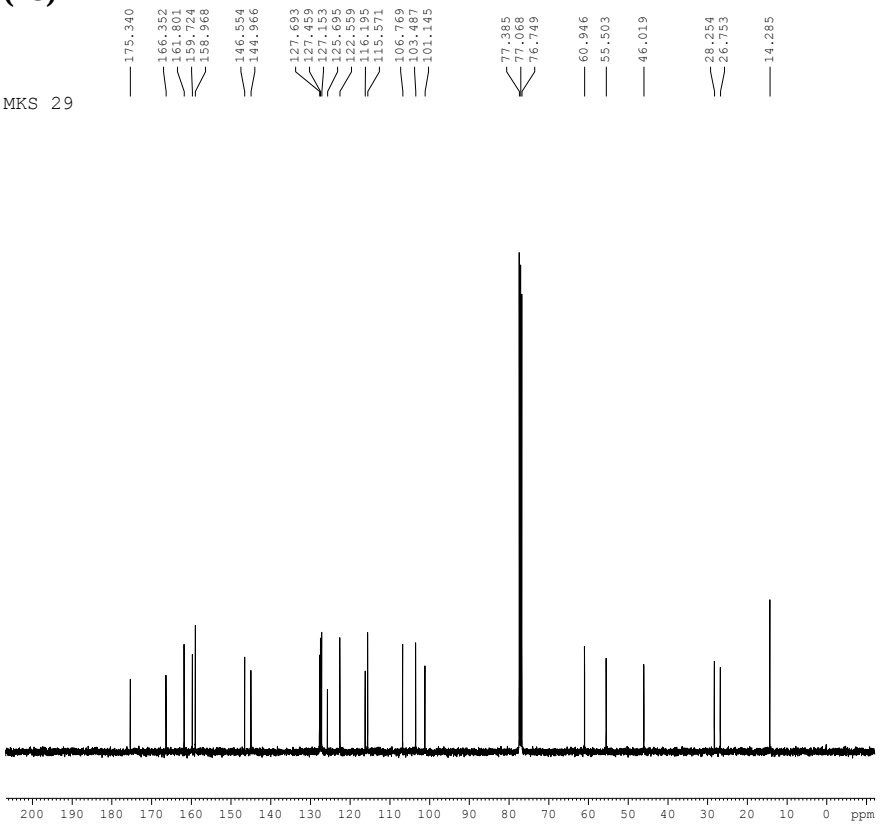


```
   BRUKER
   AVANCE II 400 NMR
   Spectrometer
   SAIF
   Panjab University
   Chandigarh
Current Data Parameters
NAME         Jul04-2017
EXPNO               341
PROCNO                1

F2 - Acquisition Parameters
Date_           20170705
Time               15.24
INSTRUM            spect
PROBHD   5 mm PABBO BB-
PULPROG          zgpg30
TD                65536
SOLVENT           CDCl3
NS                  512
DS                    4
SWH           29761.904 Hz
FIDRES         0.454131 Hz
AQ            1.1010048 sec
RG                 1440
DW               16.800 usec
DE                6.50 usec
TE                296.8 K
D1           2.00000000 sec
D11          0.03000000 sec
TD0                   1

======== CHANNEL f1 ========
SFO1         100.6228293 MHz
NUC1                 13C
P1                  9.91 usec
PLW1        41.59999847 W

======== CHANNEL f2 ========
SFO2         400.1316005 MHz
NUC2                 1H
CPDPRG[2         waltz16
PCPD2              90.00 usec
PLW2          6.80000019 W
PLW12         0.22578999 W
PLW13         0.11357000 W

F2 - Processing parameters
SI                 32768
SF           100.6127685 MHz
WDW                  EM
SSB      0
LB                1.00 Hz
GB       0
PC                1.40
```

manishkumarmanu1986@gmail.com
